# Symbiosis of the millipede parasitic nematodes Rhigonematoidea and Thelastomatoidea with evolutionary different origins

**DOI:** 10.1186/s12862-021-01851-4

**Published:** 2021-06-12

**Authors:** Seiya Nagae, Kazuki Sato, Tsutomu Tanabe, Koichi Hasegawa

**Affiliations:** 1 Department of Environmental Biology, College of Bioscience and Biotechnology, Chubu University, 1200 Matsumoto, Kasugai, Aichi 487-8501 Japan; 2 RIKEN Center for Sustainable Resource Science, 1-7-22 Suehiro-cho, Tsurumi-ku, Yokohama, Kanagawa 230-0045 Japan; 3 Faculty of Advanced Science and Technology, Kumamoto University, 2-40-1 Kurokami, Kumamoto, 860-8555 Japan

**Keywords:** Parasitic nematode, Millipede, Co-infection, Symbiosis

## Abstract

**Background:**

How various host–parasite combinations have been established is an important question in evolutionary biology. We have previously described two nematode species, *Rhigonema naylae* and *Travassosinema claudiae*, which are parasites of the xystodesmid millipede *Parafontaria laminata* in Aichi Prefecture, Japan. *Rhigonema naylae* belongs to the superfamily Rhigonematoidea, which exclusively consists of parasites of millipedes. *T. claudiae* belongs to the superfamily Thelastomatoidea, which includes a wide variety of species that parasitize many invertebrates. These nematodes were isolated together with a high prevalence; however, the phylogenetic, evolutionary, and ecological relationships between these two parasitic nematodes and between hosts and parasites are not well known.

**Results:**

We collected nine species (11 isolates) of xystodesmid millipedes from seven locations in Japan, and found that all species were co-infected with the parasitic nematodes Rhigonematoidea spp. and Thelastomatoidea spp. We found that the infection prevalence and population densities of Rhigonematoidea spp. were higher than those of Thelastomatoidea spp. However, the population densities of Rhigonematoidea spp. were not negatively affected by co-infection with Thelastomatoidea spp., suggesting that these parasites are not competitive. We also found a positive correlation between the prevalence of parasitic nematodes and host body size. In Rhigonematoidea spp., combinations of parasitic nematode groups and host genera seem to be fixed, suggesting the evolution of a more specialized interaction between Rhigonematoidea spp. and their host. On the other hand, host preference of Thelastomatoidea spp. was not specific to any millipede species, indicating a non-intimate interaction between these parasites and their hosts.

**Conclusions:**

The two nematode superfamilies, Rhigonematoidea and Thelastomatoidea, have phylogenetically distinct origins, and might have acquired xystodesmid millipede parasitism independently. Currently, the two nematodes co-parasitize millipedes without any clear negative impact on each other or the host millipedes. Our study provides an example of balanced complex symbioses among parasitic nematodes and between parasitic nematodes and host millipedes, which have been established over a long evolutionary history.

**Supplementary Information:**

The online version contains supplementary material available at 10.1186/s12862-021-01851-4.

## Background

How parasite diversity has evolved is an important question to understand host–parasite interactions, which form the basis of sustainable development. Nematoda is a large and ancient phylum that exhibits significant ecological variety. Free-living nematodes occupy almost all terrestrial, marine, and freshwater habitats, ranging from tropical to polar environments, with the number of individuals exceeding all other organisms [20, 38, 69]. Parasitic or phoretic nematodes live on or inside other organisms, and their diversity represents the fact that each host is associated with at least one unique nematode [5, 57]. Some parasitic nematodes live together with hosts as commensals [12, 24, 25, 54] or mutualistic partners [39], but others are harmful to the hosts [4, 7, 17, 56]. These four interactions—phoresis, commensalism, parasitism, and mutualism—are all recognized as subcategories of symbiosis [15], and are commonly observed in the phylum Nematoda. Their original symbiotic interactions with native hosts are sometimes disrupted by human activities, which can trigger devastating epidemics. Examples include pine wilt disease in Eastern Asia and Europe caused by the invasive pine-wood nematode *Bursaphelenchus xylophilus* [22], and crop diseases caused by a variety of plant parasitic/pathogenic nematodes in agricultural fields globally [61].

According to phylogenetic analysis, the phylum Nematoda is divided into five clades [5]. Like all living organisms, the Nematoda are believed to be of marine origin. The most primitive subclass is Enoplia (Clade II), most of which consists of free-living marine species. However, some nematode groups in the subclass Enoplia have evolved in terrestrial ecosystems and have developed plant and animal parasitism [6]. The next subclass to branch from Enoplia was Dorylaimia (Clade I), which contains free-living freshwater and terrestrial nematodes, and plant- and animal-parasitic nematodes. For example, known animal and plant pests, such as *Trichinella* and *Dorylaimida*, occur in the subclass Dorylaimia [6]. Another subclass that branched off, Chromadoria, is divided into three suborders: Rhabditina (Clade V), Tylenchina (Clade IV), and Spirurina (Clade III) [5, 6, 70]. Animal and plant parasitism have evolved independently in these five clades, and all nematodes belonging to the suborder Spirurina are animal parasites [5, 6, 70].

Since millipedes (class Diplopoda) are believed to be the first animals to inhabit terrestrial environments during the Silurian period (ca. 420 mya) [23, 41], it is a good model for understanding the evolutionary and ecological relationships between these parasitic nematodes and between hosts and parasites. We previously described the two nematode species, *Rhigonema naylae* (Rhigonematoidea) and *Travassosinema claudiae* (Thelastomatoidea), which are parasites of the xystodesmid millipede species *Parafontaria laminata* (Polydesmida: Xystodesmidae) in Aichi Prefecture, Japan [45, 46]. Both nematode species are in the suborder Spirurina, but in different infraorders. *Rhigonema naylae* belongs to the infraorder Rhigonematomorpha, which includes the millipede parasites Ransomnematoidea [40, 42] and Rhigonematidae [32, 45, 46], and the amphibian and reptile parasite Cosmocercoidea [8, 9]. *Travassosinema claudiae* is a member of the Oxyuridomorpha, which includes a wide variety of parasites, such as vertebrate-parasitic Oxyuroidea [49], invertebrate-parasite Coronostomatoidea [55], and Thelastomatoidea [53, 47, 48]. The family Thelastomatidae mainly includes cockroach parasites and is a highly diversified group characterized by low host specificity [2, 35, 54].

Here, we reveal that the two parasitic nematodes, Rhigonematoidea spp. and Thelastomatoidea spp., have different evolutionary origins but co-infect the xystodesmid millipedes without clear negative impacts between each parasitic nematode and host millipedes.

## Results

### Two parasitic nematodes *R. naylae* and *T. claudiae* isolated from the two millipedes

#### Parafontaria laminata CU and Parafontaria tonominea species complex CU

**Fig. 1:**
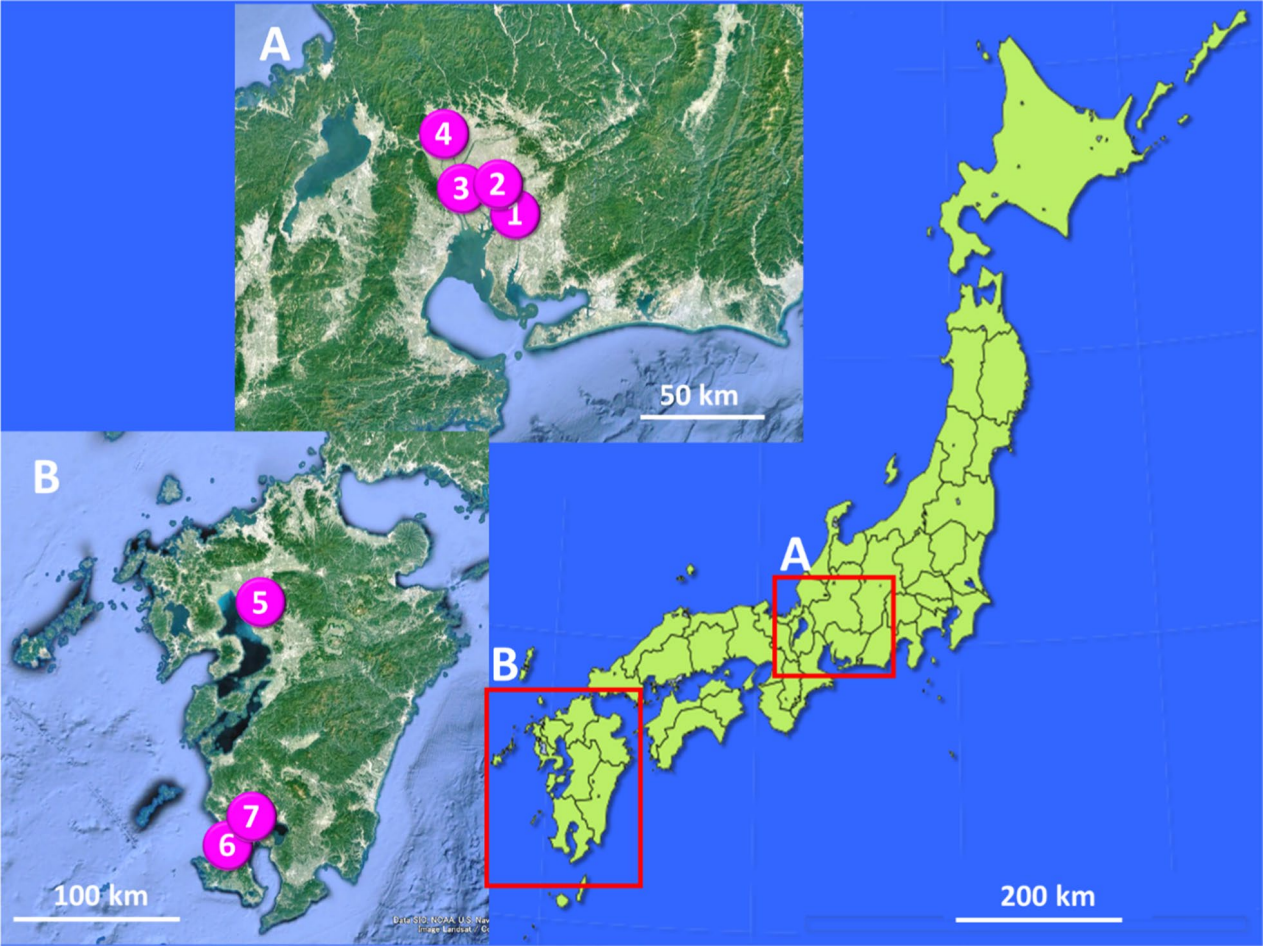
Geographical location of millipede collection sites. The zoomed maps A and B from the red squared on the map of Japan are created using Google Earth. Location numbers are, 1 Chubu University, Kasugai City, Aichi Prefecture. 2 Mt. Kinka, Gifu City, Gifu Prefecture. 3 Hyakunen Park, Seki City, Gifu Prefecture. 4 Embara, Yamagata City, Gifu Prefecture. 5 Mt. Shimono, Yamaga City, Kumamoto Prefecture. 6 Miyanoura, Kagoshima City, Kagoshima Prefecture. 7 Shiroyama, Kagoshima City, Kagoshima Prefecture

**Fig. 2:**
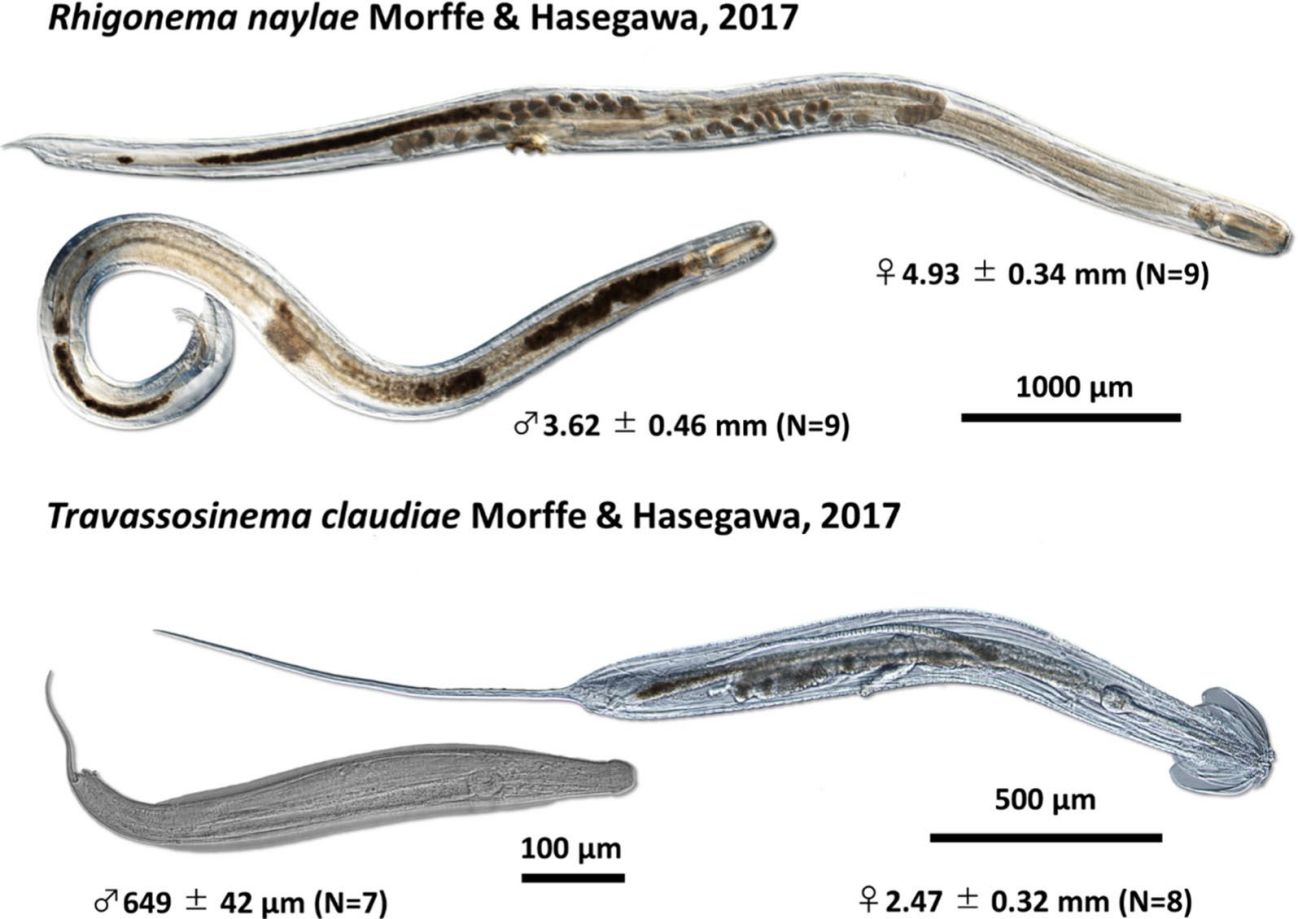
Two parasitic nematodes, *Rhigonema naylae* and *Travassosinema claudiae* parasites of the xystodesmid millipede *Parafontaria laminata* CU and *P. tonominea* species complex CU. Nomarski differential interference construct images of adult female and male. Body sizes (Average ± S.D.) are adapted from [45, 46]

We isolated two parasitic nematodes*, R. naylae* and *T. claudiae*, from the xystodesmid millipede, *P. laminata*, in the Chubu University campus in Aichi Prefecture, Japan (Table 1, Fig. 1). Hereafter, this millipede species is referred to as *P. laminata* CU. *Rhigonema naylae* has a large body size, short pharynx, round head, and short tail. The body size of *T. claudiae* is smaller than that of *R. naylae*; the females have a typical umbrella-like head and long tail, and the males have a tiny body with a spicule (Fig. 2) [45, 46]. No clear sexual dimorphism was detected in either nematode species during the juvenile stage; however, it was possible to distinguish the two species at any developmental stage.

**Table 1:** Summary of collection data of millipedes and parasitic nematodes

Location	Host millipede (number)	Nematode (Accession No.)
1. Chubu University, Kasugai City Aichi Prefecture 35°16′21.6″N 137°00′50.3″E	*P. laminata* CU (N = 113)	*R. naylae* (MT988356–MT988360) *T. claudiae* (MT988324, MT988325, MT988326)
	*P. tonominea* species complex CU (N = 82)	*R. naylae* (MT988354, MT988355) *T. claudiae* (MT988321, MT988322, MT988323)
2. Mt. Kinka, Gifu City Gifu Prefecture 35°25′55.9″N 136°47′31.0″E	*P. laminata* Kinka (N = 35)	*R. naylae* (MT988366, MT988367) *T. claudiae* (MT988332, MT988333)
	*P. tonominea* species complex Kinka (N = 43)	*R. naylae* (MT988364, MT988365) *T. claudiae* (MT988330, MT988331)
3. Hyakunen Park, Seki City Gifu Prefecture 35°28′32.2″N 136°52′27.8″E	*P. tonominea* species complex Hyaku (N = 22)	*R. naylae* (MT988361, MT988362, MT988363) *T. claudiae* (MT988327, MT988328, MT988329)
4. Embara, Yamagata City Gifu Prefecture 35°39′42.1″N 136°44′02.0″E	*P. tonominea* species complex Embara (N = 20)	*R. naylae* (MT988368, MT988369, MT988370) *T. claudiae* (MT988334, MT988335, MT988336) *Cephalobellus* sp. 1 (MT988351-MT988353)
	*P. longa* Embara (N = 7)	*R. naylae* (MT988371) *T. claudiae* (MT988337 MT988338)
5. Mt. Shimono, Yamaga City Kumamoto Prefecture 32°56′27.6″N 130°38′58.4″E	*R. cornuta* Yamaga (N = 31)	Rhigonematoidea sp. 1 (MT988372, MT988373) *T. claudiae* (MT988339, MT988340, MT988341) Thelastomatidae sp. 1 (MT988313, MT98314)
6. Miyanoura, Kagoshima City Kagoshima Prefecture 31°26′00.4″N 130°28′05.0″E	*R. anachoreta* Miya (N = 20)	Rhigonematoidea sp. 1 (MT988374) *T. claudiae* (MT988342, MT988343, MT988344) Thelastomatidae sp. 2 (MT988315)
	*R. semicircularis semicircularis* Miya (N = 9)	Rhigonematoidea sp. 1 (MT988375, MT988376) *T. claudiae* (MT988345, MT988346, MT988347) Thelastomatidae sp. 2 (MT988316, MT98317, MT98318)
7. Shiroyama, Kagoshima City Kagoshima Prefecture 31°35′54.0″N 130°33′00.1″E	*R. semicircularis semicircularis* Shiroyama (N = 38)	Rhigonematoidea sp. 1 (MT988377, MT988378) *T. claudiae* (MT988348, MT988349, MT988350) Thelastomatidae sp.2 (MT988319, MT988320)

A total of 113 *P. laminata* CU millipedes were captured from April 2018 to July 2019, and parasitic nematode infections were examined. The total infection prevalence (counted as present when at least one individual was detected from any developmental stage) of *R. naylae* and *T. claudiae* was 31.0% and 27.4%, respectively, and co-infection prevalence (infected by the two nematode species together) was 10.6% (Table 2). The density (number of males, females, and juveniles of nematodes in individual hosts) and infection percentage at each stage are also presented in Table 2. We found 11.5% of males and 12.4% of females of *R. naylae*, and 1.8% of males and 3.5% of females of *T. claudiae* (Table 2). All adult male and female nematodes appeared healthy, and all females matured, and many eggs were observed in the uteri.

**Table 2:** Population of the two parasitic nematodes in the two *Parafontaria* millipedes

Host	Parasitic nematodes					
		Male densities^1^ and prevalence^2^	Female densities^1^ and prevalence^2^	Juvenile densities^1^ and prevalence^2^	Total prevanence^2^	Co-infection prevalence
*Parafontaria laminata* CU (N = 113)	* R. naylae*	3.38 (2.31–4.38)	3.43 (1.93–6.07)	7.16 (5.00–9.76)	31.0%	10.6%
		11.5% (N = 13)	12.4% (N = 14)	22.1% (N = 25)	N = 35	N = 12
	* T. claudiae*	1.5*	1.75 (1.00–2.55)	8.13 (5.48–12.45)	27.4%	
		1.8% (N = 2)	3.5% (N = 4)	27.4% (N = 31)	N = 31	
*Parafontaria tonominea* species complex CU (N = 82)	* R. naylae*	10.99 (9.55–12.7)	11.47 (9.86–13.07)	24.03 (18.61–33.57)	96%	72%
		89% (N = 73)	90% (N = 74)	92% (N = 75)	N = 79	N = 59
	* T. claudiae*	1.90 (1.40–2.60)	5.93 (4.77–7.32)	4.53 (2.47–8.05)	72%	
		24% (N = 20)	68% (N = 56)	23% (N = 19)	N = 59	

^1^Mean densities and confidence intervals with 95% confidence limit (in brackets) were calculated by Bootstrap Confidence interval method

^2^% Of the infected millipede among all millipede examined

*Confidence intervals were not calculated if the sample size was too small

Another xystodesmid millipede in the same area was also infected with the two parasitic nematodes. From the body shape and male genitalia, this millipede was determined to be a member of the *P. tonominea* species complex (Additional file 1: Figure S1) [67]. Hereafter, this millipede species is referred to as *P. tonominea* species complex CU. A total of 82 *P. tonominea* species complex CU millipedes were captured from May 2018 to July 2019, and parasitic nematode infections were examined. The infection prevalence of both parasitic nematodes in the *P. tonominea* species complex CU was higher than that in *P. laminata*. The total infection prevalence (counted as present when at least one individual was detected from any developmental stage) of *R. naylae* and *T. claudiae* was 96% and 72%, respectively, and the co-infection prevalence was 72% (Table 2). A total of 89% of males and 87% of females of *R. naylae*, and 52% of males and 17% of females of *T. claudiae* were detected (Table 2). All females of both nematode species in the *P. tonominea* species complex CU were mature, and many eggs were present in the uteri. From these results, we deduced that both parasitic nematodes*, R. naylae* and *T. claudiae,* were able to use the two millipedes as hosts; however, the infection prevalence and density of *T. claudiae* were lower than those of *R. naylae*.

### Population of *R. naylae* was not negatively affected by co-infection with *T. claudiae*

**Fig. 3:**
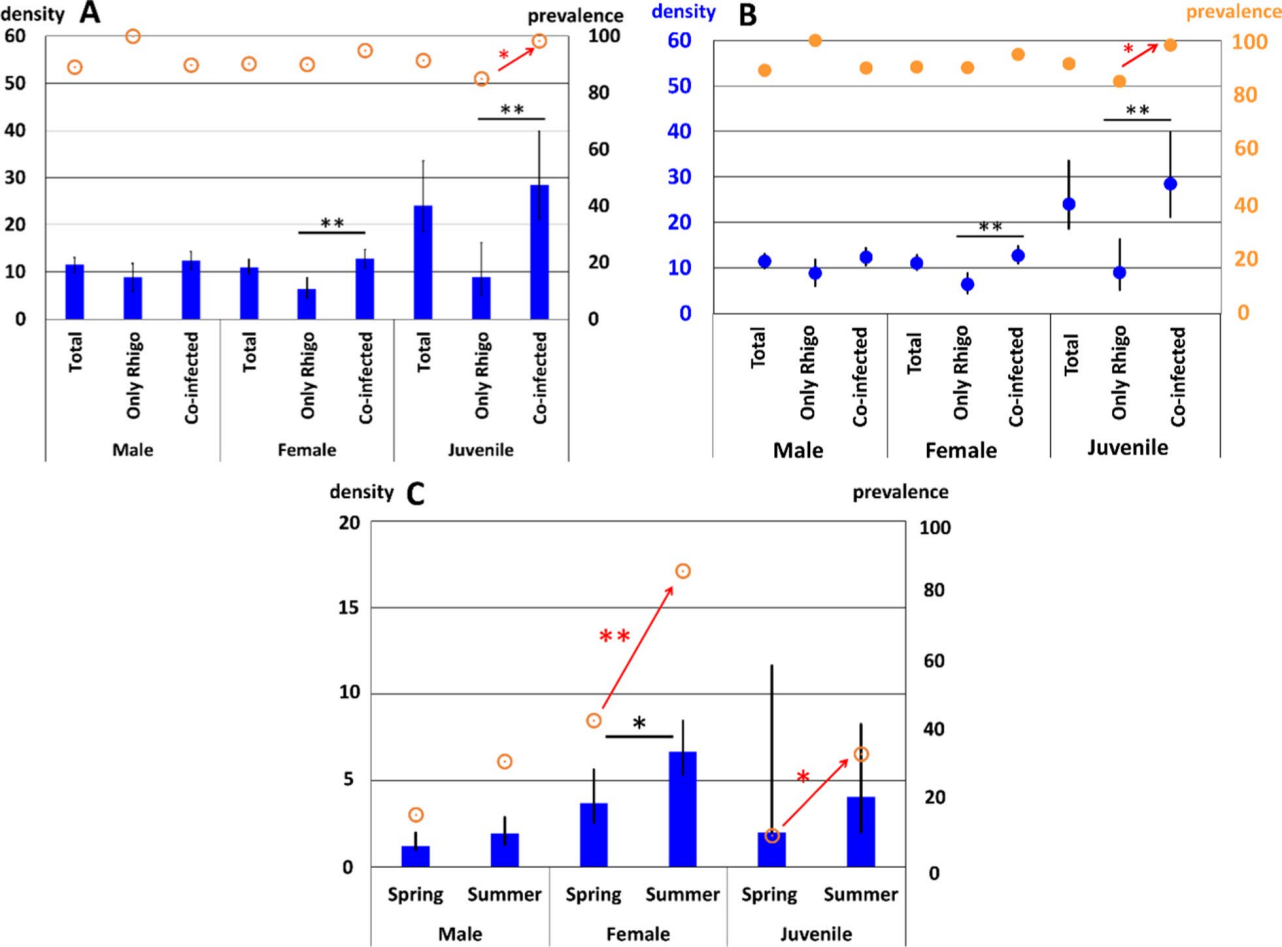
Infection prevalence (%) (orange dot, right Y-axis) and mean density (mean number of nematodes per infected host) (blue dot, left Y-axis) of male, female, and juvenile of the parasitic nematodes in *Parafontaria tonominea* species complex CU. **A** Infection prevalence and density of *R. naylae* in all the hosts (N = 82), hosts infected with only *Rhigonema naylae* (N = 20), and the hosts co-infected with *R. naylae* and *Travassosinema claudiae* (N = 59). **B** Prevalence and density of *R. naylae* in all the hosts collected during spring (N = 33) and summer (N = 49). **C** Infection prevalence and density of *T. claudiae* in all hosts collected during spring (N = 33) and summer (N = 49). Error bars indicate confidence interval with 95% confidence limit. *p < 0.05, **p < 0.005, Fisher’s exact test for comparing prevalence, and Bootstrap 2-sample t-test for comparing mean densities

We analyzed whether the two parasitic nematodes*, R. naylae* and *T. claudiae*, affected each other when they co-infected the host millipede *P. tonominea* species complex CU. There were three infection patterns: (1) co-infection with the two parasitic nematodes (N = 59), (2) infection with only *R. naylae* (N = 20), and (3) no nematode infection (N = 3). We did not observe any millipede host *P. tonominea* species complex CU infected with *T. claudiae* alone. We studied the infection prevalence % and mean density (mean number of nematodes per infected host) of male, female, and juvenile *R. naylae* in: (1) all the hosts (N = 82), (2) hosts infected with only *R. naylae* (N = 20), and (3) hosts co-infected with *R. naylae* and *T. claudiae* (N = 59). The infection prevalence of *R. naylae* juveniles in hosts co-infected with *T. claudiae* was significantly higher than that in hosts infected with only *R. naylae*, but there were no differences in the prevalence of *R. naylae* adults (Fig. 3A). In addition, the mean densities of *R. naylae* females and juveniles were significantly higher when co-infected with *T. claudiae* (Fig. 3A). The effects of host body size on these infection conditions was not significantly different (Additional file 2: Figure S2). Thus, co-infection with *T. claudiae* did not affect the population of *R. naylae* in the alimentary tract of the *P. tonominea* species complex CU.

We also checked the effects of seasonal differences (spring vs. summer) on the prevalence and densities of the two parasitic nematodes in the host millipede *P. tonominea* species complex CU. The infection prevalence of *R. naylae* at all stages was high, and there was no significant difference between spring (91%, N = 33) and summer (96%, N = 49) (Fig. 3B). The density of *R. naylae* juveniles was significantly higher during summer (Fig. 3B). As indicated previously, the infection prevalence of *T. claudiae* was lower than that of *R. naylae*; however, female prevalence in summer was higher (86%, N = 42), and those of the females and juveniles of *T. claudiae* significantly increased during summer (Fig. 3C). The density of *T. claudiae* females was also higher during summer (Fig. 3C). The effects of host body size on these seasonal conditions were not significantly different (Additional file 2: Figure S2). Thus, while the density and prevalence of *R. naylae* remained high year-round, those of *T. claudiae* were strongly affected by seasonal changes.

### Two nematodes commonly co-infected in *Parafontaria* millipedes

**Fig. 4:**
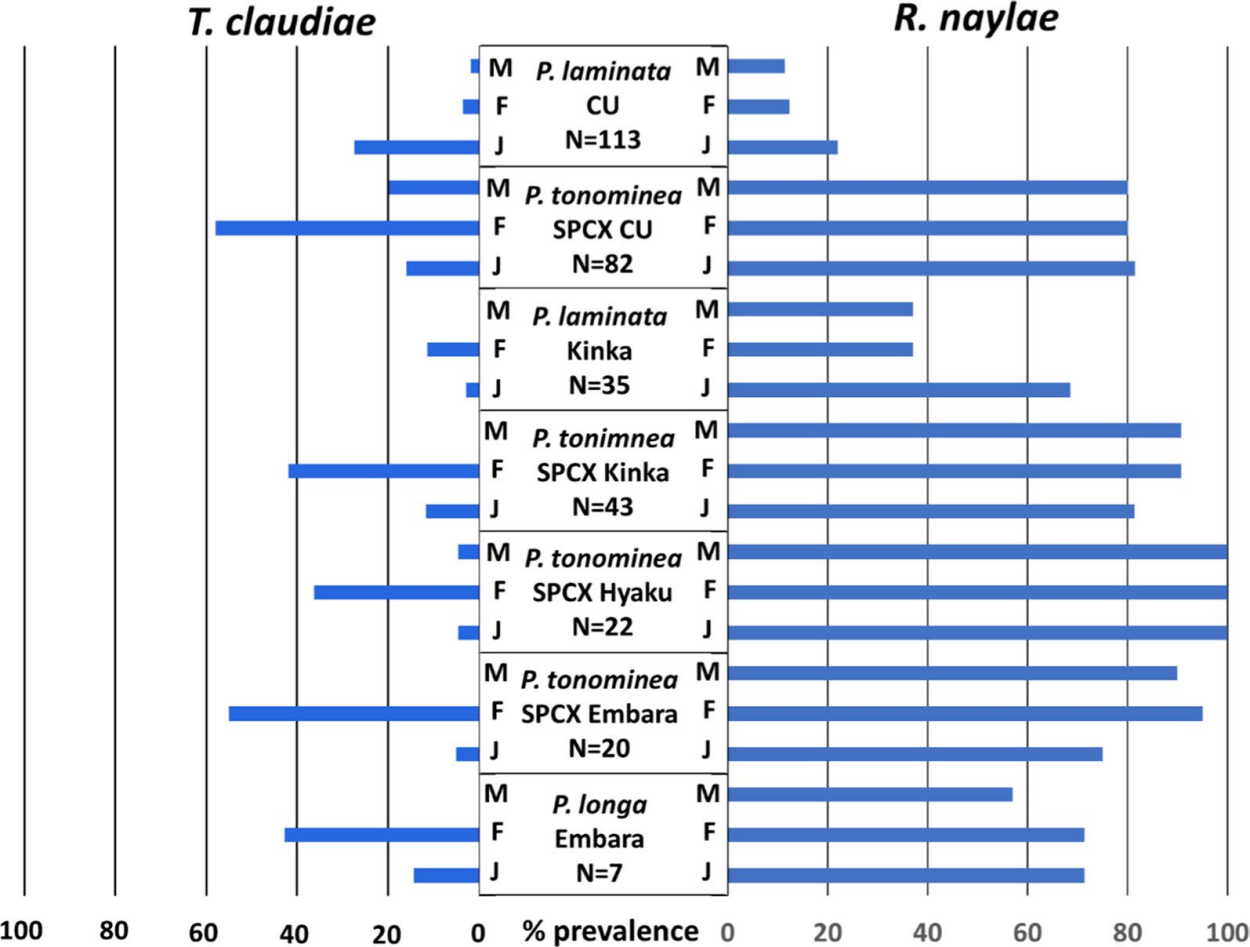
Infection prevalence (%) of the two parasitic nematodes males (M), females (F), and juveniles (J) in each *Parafontaria* millipede. Details of the host information are in Table 1

We also collected five *Parafontaria* species from three other locations in Gifu Prefecture (Fig. 1, Table 1)*.* From morphological observations, one was *P. laminata* Kinka (collected from Mt Kinka, Gifu City), and belonged to the same species as *P. laminata* CU; another was *P. longa* Embara (collected from Embara, Yamagata City); and the three other millipedes were different species that were tentatively classified as members of the *P. tonominea* species complex (Additional file 1: Figure S1). Similar to the *P. tonominea* species complex CU and *P. laminata* CU, these five *Parafontaria* millipedes tended to be infected with the two parasitic nematodes, *R. naylae* and *T. claudiae.* All D2D3 LSU sequences within the species were almost identical (Additional files 3, 4: Tables S1 and S2). Infection prevalence and mean density of parasitic nematode males, females, and juveniles are shown in Fig. 4 and Additional file 5: Table S3. The characteristics of the two nematodes in the *Parafontaria* species were similar, and the infection prevalence of *R. naylae* was higher than that of *T. claudiae*; however, all adult male and female nematodes matured and reproduced.

In addition to the two parasitic nematodes, unknown species were isolated from the four host species. Two individuals of *P. tonominea* species complex Kinka (2/43, 4.5%), two of *P. laminata* Kinka (2/35, 5.7%), eight of *P. tonominea* species complex Embara (8/20, 40%)*,* and one of seven *P. longa* Embara (1/7, 14%) were infected. These nematodes were morphologically distinguishable from *R. naylae* and *T. claudiae*. D2D3 LSU sequencing data from the nematode isolated from the *P. tonominea* species complex Embara showed that it had a similar sequence to *Cephalobellus brevicaudatus* [14], MF668725.1 with 92% identity (671/727 bp). Combined with morphological observations, this nematode is an undescribed species, and we temporarily named it *Cephalobellus* sp.1 (Table 1). We could not identify the other unknown nematodes because of the small number of specimens.

### Parasitic nematodes in *Riukiaria* millipedes

To determine the infection patterns of parasitic nematodes in other xystodesmid millipedes, we collected *Riukiaria* spp. from Kyushu Island. Three species (four isolates) were collected from three locations in Kyushu (Table 1, Additional file 1: Figure S1). All of the species tended to be infected with the three parasitic nematodes, Rhigonematoidea sp. 1, *T. claudiae*, and Thelastomatidae spp. (Table 1). From the D2D3 LSU sequencing data, Thelastomatidae sp. 1 and sp. 2 and Rhigonematoidea sp.1 were undescribed species (Additional files 3, 6: Tables S1 and S4). Surprisingly, *T. claudiae*, the species that was isolated from *Parafontaria* millipedes, was also found in all *Riukiaria* millipedes in our samples (Table 1, Additional file 4: Table S2). Thelastomatidae sp. 1 and Thelastomatidae sp. 2 were combined and referred to as Thelastomatidae spp.

**Fig. 5:**
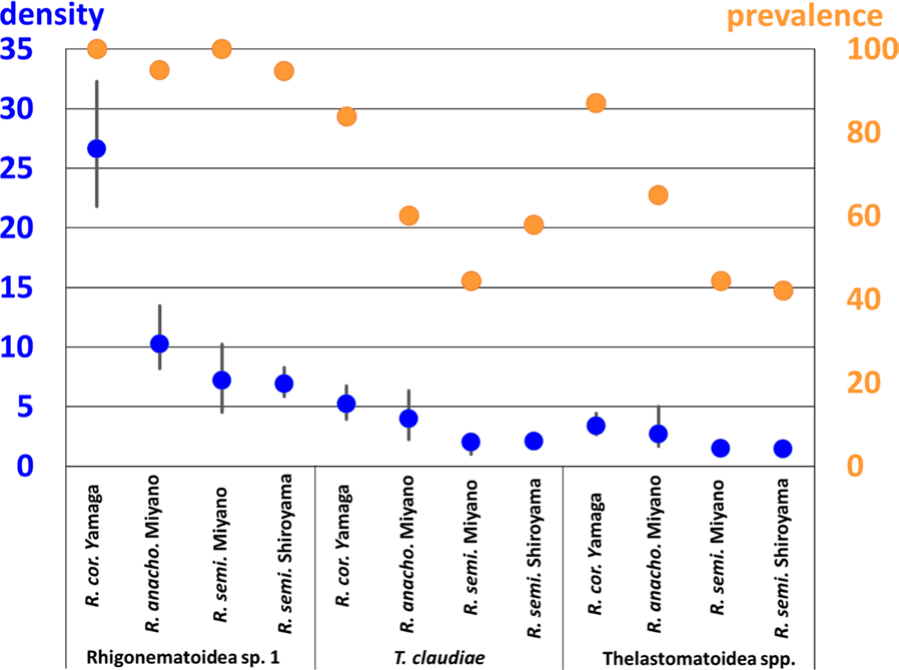
Infection prevalence (%) (orange dot, right Y-axis) and mean density (the mean number of nematodes per infected host) (blue dot, left Y-axis) of the females of the parasitic nematodes. Error bars indicate confidence intervals with 95% confidence limit

Similar to *R. naylae* in *Parafontaria* millipedes, the infection prevalence of male, female, and juvenile Rhigonematoidea sp.1 was high (67–100%), whereas the infection prevalence of *T. claudiae* and Thelastomatidae spp. was lower (0–87%) (Additional file 7: Table S5). In addition, males and juveniles of *T. claudiae* and Thelastomatidae spp. in *Riukiaria* hosts were detected only in a few cases. Infection data of only nematode females were selected and are shown in Fig. 5. Since all of the females were mature, and many eggs in their uteri were visible, we concluded that all of these parasitic nematodes used *Riukiaria* millipedes as hosts and could reproduce.

**Fig. 6:**
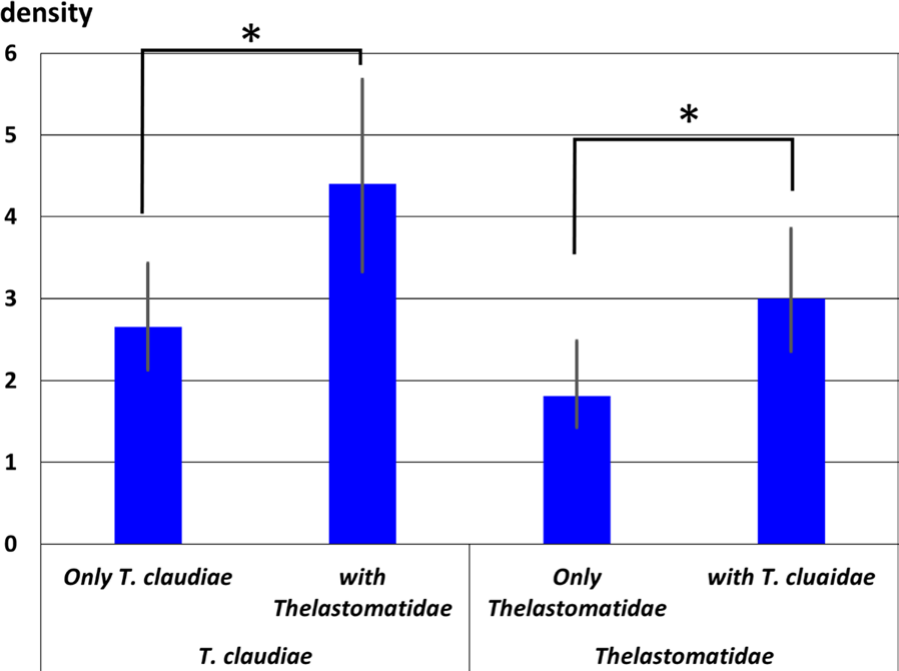
Mean density of *Travassosinema claudiae* adult female in all *Riukiaria* spp. (1) infected with only *T. claudiae* and (2) co-infected with Thelastomatidae spp., and mean density of Thelastomatidae spp. adult female in all *Riukiaria* spp. (3) infected with only Thelastomatidae spp. and (4) co-infected with *T. claudiae.* Error bars indicate confidence interval with 95% confidence limit. *p < 0.05, Bootstrap 2-sample t-test for comparing mean densities

We then analyzed whether the two parasitic nematodes*, T. claudiae* and Thelastomatidae spp., affect each other when co-infecting host millipedes. The mean densities of *T. claudiae* adult females in all *Riukiaria* spp. were combined and calculated for two infection patterns: (1) infected with only *T. claudiae*, and (2) co-infected with *T. claudiae* and Thelastomatidae spp. In addition, the mean densities of Thelastomatidae spp. adult females in *Riukiaria* spp. were calculated for two infection patterns: (3) infected with only Thelastomatidae spp., and (4) co-infected with *T. claudiae* and Thelastomatidae spp. (Fig. 6). Since 99% of the *Riukiaria* millipedes (N = 97 of 98) were infected with Rhigonematoidea sp.1, we could not examine the effect of Rhigonematoidea sp.1 on the densities of Thelastomatidae spp. and *T. claudiae*. The densities of both parasitic nematodes in co-infected hosts were significantly higher than those in single infections (Fig. 6).

**Fig. 7:**
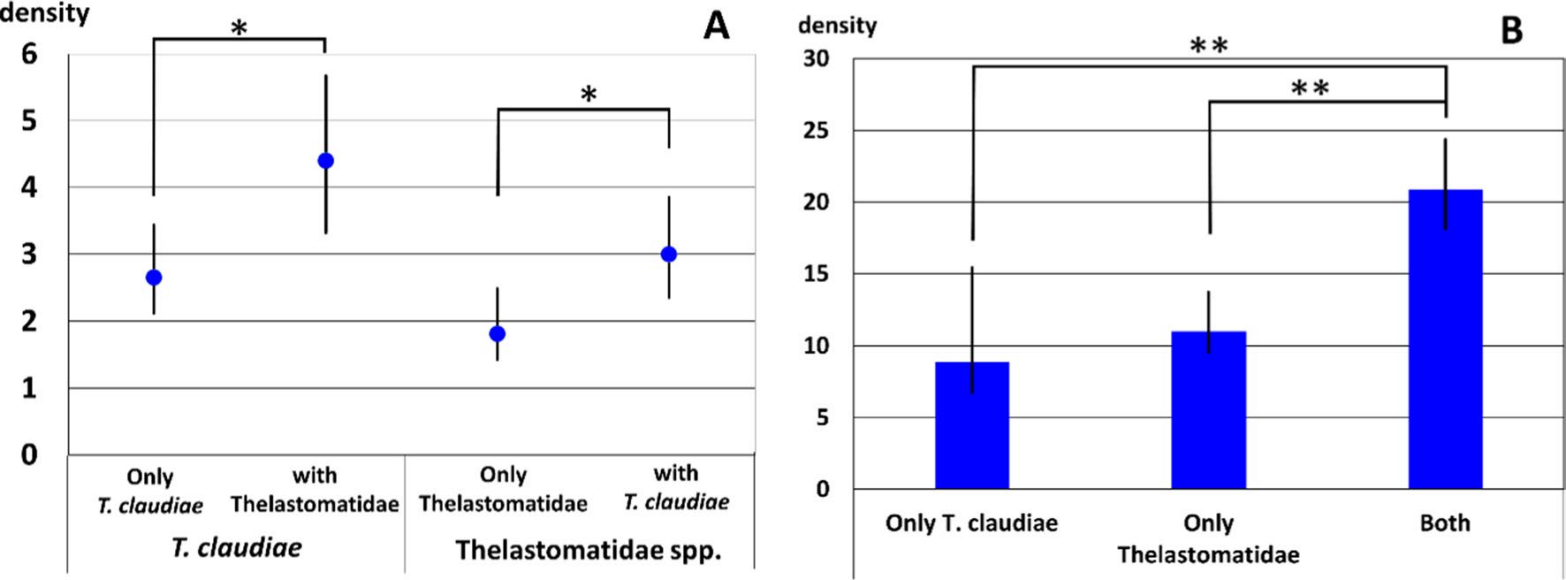
**A** Mean density of Rhigonematoidea sp. 1 adult female, in *Riukiaria* spp. infected with only *Travassosinema claudiae*, only Thelastomatidae spp., and both parasites. Error bars indicate confidence intervals with 95% confidence limit. *p < 0.05, Bootstrap 2-sample t-test for comparing mean densities. **B** Average ± S.D. of body length of host millipede *Riukiaria* spp., infected with only *T. claudiae*, only Thelastomatidae spp., or both parasites. **p < 0.005, Statistical differences were analyzed by Tukey’s multiple comparison test followed by Bonferroni correction

Furthermore, we found that the mean densities of Rhigonematoidea sp.1 adult females were significantly higher when the two parasitic nematodes Thelastomatidae spp. and *T. claudiae* were co-infected in the host at the same time, compared with those with only Thelastomatidae spp. or *T. claudiae* (Fig. 7A). Since the mean body size of *Riukiaria* spp. was larger when co-infected with the two parasitic nematodes Thelastomatidae spp. and *T. claudiae* (Fig. 7B), the prevalence of these two parasites was positively correlated with host body size. Thus, the number of parasites in millipede hosts increased with increasing host size, suggesting that these nematodes did not compete and eliminate each other.

### Parasitic nematodes in xystodesmid millipedes with phylogenetically different origins

**Fig. 8:**
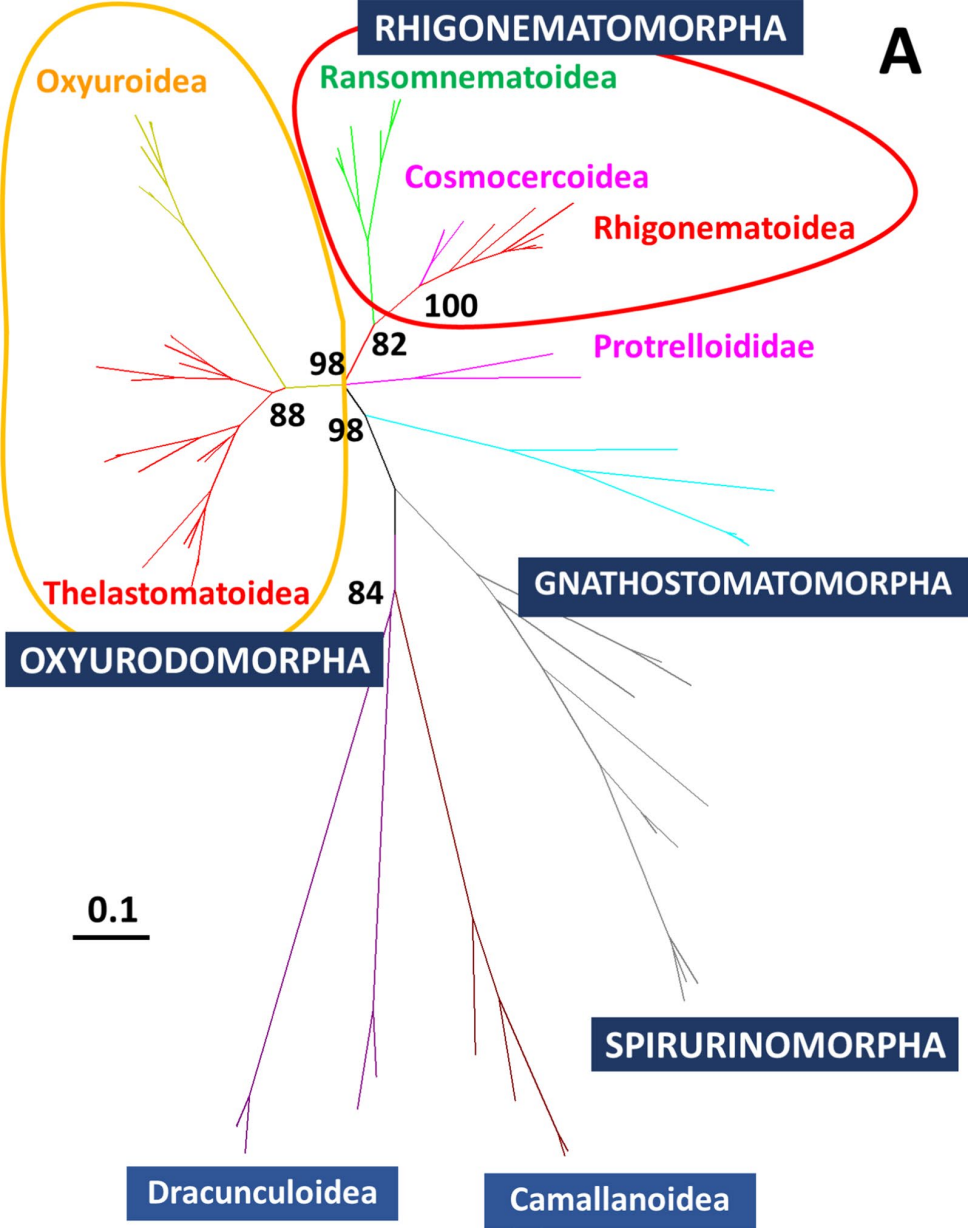

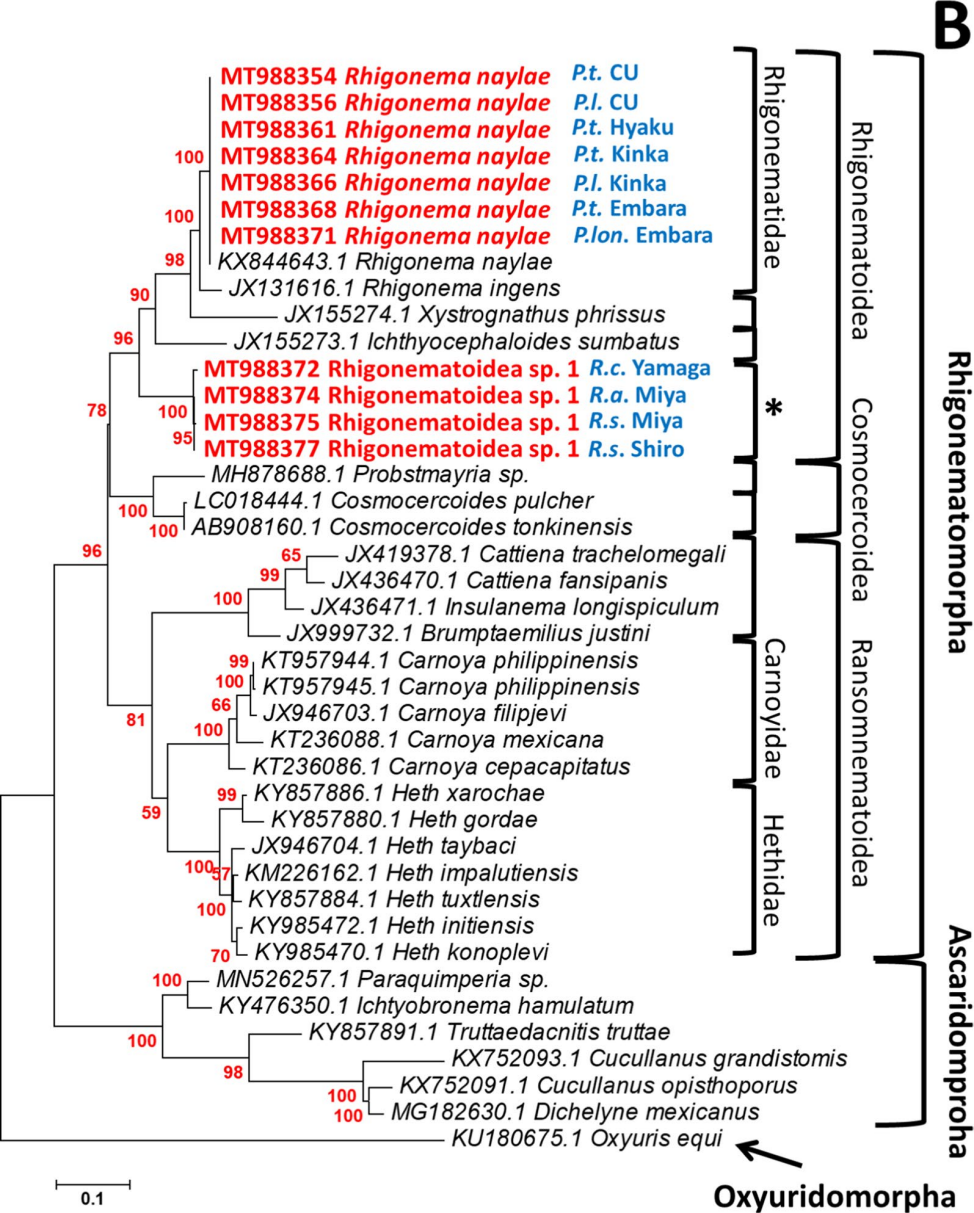

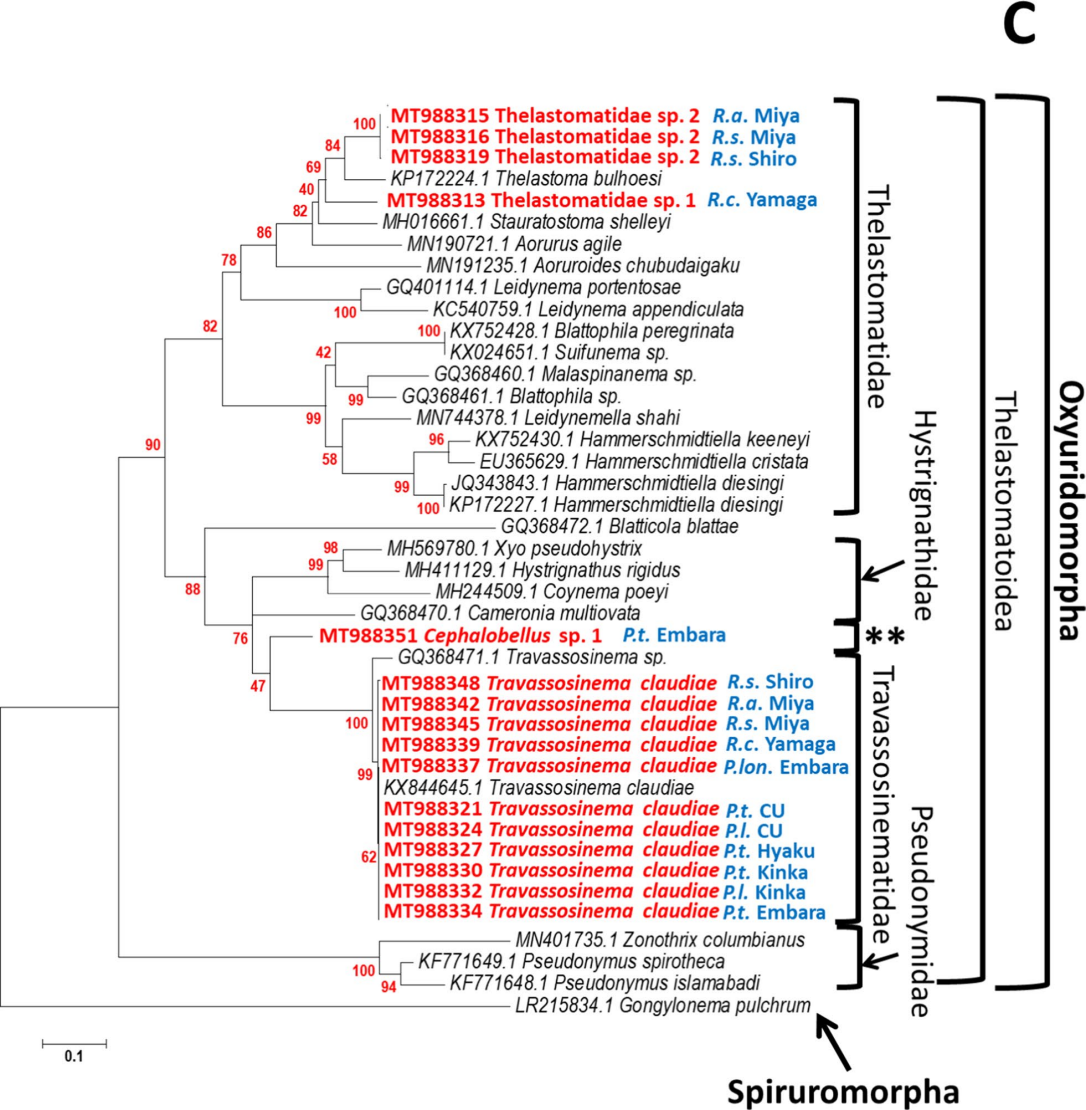
Phylogenetic trees of parasitic nematodes inferred from the D2D3 LSU rRNA. Bootstrap values with 1000 replicates are shown next to the branches. **A** Phylogenetic tree of the suborder Spirurina, constructed using the neighbor-joining (NJ) method. **B** Phylogenetic tree of Rhigonematomorpha, constructed using the maximum likelihood (ML) method. Newly obtained sequences are in red. *The position of Rhigonematoidea sp.1. is emphasized. **C** Phylogenetic tree of the Thelastomatoidea constructed using the ML method. Newly obtained sequences are in red, and their hosts are in blue. **The position of *Cephalobellus* sp.1 is emphasized

We used D2D3 LSU rDNA genes selected from representatives of the four infraorders Oxyuridomorpha, Rhigonematomorpha, Gnathostomatomorpha, Spirurinomorpha, and the two families Dracunculoidea and Camallanoidea (Additional file 8: Table S6). A phylogenetic tree covering the suborder Spirurina was constructed that supported the taxonomic relationship previously reported [37, 43], these infraorders were clearly separated and clustered (Fig. 8A). Rhigonematoidea was clustered with Cosmocercoidea and Ransomnematoidea. As Travassosinematidae appeared to be close to Thelastomatidae, and both are classified in Thelastomatoidea (Fig. 8A), the xystodesmid millipedes examined in this study tended to be infected with parasitic nematodes belonging to these two infraorders.

In the maximum likelihood (ML) tree, Rhigonematoidea sp. 1 was clearly separated from the Rhigonematidae cluster but could be within Rhigonematoidea (Fig. 8B). All parasitic nematodes in Rhigonematomorpha are believed to be millipede parasites, but Cosmocercoidea (reptile and amphibian parasites) were included in Rhigonematomorpha in our phylogenetic analysis (Fig. 8B). The parasitic nematodes *T. claudiae*, *Cephalobellus* sp. 1, Thelastomatidae sp. 1, and Thelastomatidae sp. 2 in our experiments were positioned in Thelastomatoidea (Fig. 8C). The closest group to *Cephalobellus* sp. 1 was Travassosinematidae, and it was clearly separated from Thelastomatidae. Both Thelastomatidae sp. 1 and sp. 2 were positioned close to the cockroach-parasitic nematode *Thelastoma bulhoesi* (Fig. 8C).

## Discussion

Two nematode species, *R. naylae* and Rhigonematoidea sp. 1 in the superfamily Rhigonematoidea, were isolated from millipede hosts *Parafontaria* spp. and *Riukiaria* spp., respectively, with high infection prevalence (Table 1). Phylogenetic analysis with rRNA SSU, rRNA LSU, and mitochondrial DNA indicated that Rhigonematomorpha is not monophyletic, but is nested within Ascaridomorpha [36, 43]. The superfamily Cosmocercoidea was also positioned within the Rhigonematomorpha in our analysis (Fig. 8B). This superfamily contains reptile and amphibian parasitic nematodes, and is classified within Ascaridomorpha [8, 9, 50]. Thus, further studies are required on the taxonomy of these groups; however, these infraorders are clearly closely related phylogenetically. In addition, the infraorder Rhigonematomorpha is composed of Rhigonematoidea and Ransomnematoidea and contains exclusively millipede parasites [19, 33]. We also showed that the combination of parasitic nematode groups and host genera seems to be specifically fixed. For example, *Riukiaria* hosts were exclusively infected with Rhigonematoidea sp. 1, whereas *Parafontaria* hosts were exclusively infected with *R. naylae* (Fig. 8B)*.* Combining these data, we predicted the appearance of Rhigonematoidea, which might have divided from a common ancestor with Ascaridomorpha and acquired millipede parasitism at an earlier period before millipedes began to inhabit terrestrial environments.

The infection cycle of Rhigonematoidea is not well known; however, nematode eggs laid by adult females are probably deposited within their host feces, similar to parasitic nematodes in Oxyuridomorpha. When eggs were collected from the adult females of *R. naylae* and incubated in phosphate buffered saline (PBS) buffer, they began to develop, and larvae hatched (data not shown). These parasites might have a special mechanism that specifically infects only millipedes. For example, larvae are only attracted to specific hosts. In addition, autoinfection might occur similar to that in human parasitic nematode *Strongyloides stercoralis* [73], resulting in repeated generations occurring in the same host individual, leading to high infection prevalence throughout the season (Fig. 3B).

Four nematode species in Thelastomatoidea, *T. claudiae*, *Cephalobellus* sp. 1, Thelastomatidae sp. 1, and Thelastomatidae sp. 2 were isolated from xystodesmid millipedes (Table 1). *Cephalobellus* sp. 1 was only found in the *P. tonominea* species complex Embara, and Thelastomatidae sp. 1 and Thelastomatidae sp. 2 were only found in *Riukiaria* spp. Interestingly, *R. naylae* infected all of the millipedes. *Cephalobellu*s is a member of the family Thelastomatidae and is found in many invertebrate hosts, including mole crickets, crane flies, white grubs, cockroaches, and millipedes [14, 29, 60]. In addition, we isolated nematode genera from the mole cricket *Gryllotalpa orientalis*, white grub *Protaetia orientalis submarumorea*, and pill millipedes *Hyleoglomeris japonica* (data not shown). *Travassosinema* is currently one of three genera (together with *Indiana* and *Pulchrocephala*) in the family Travassosinematidae [1, 28]. Although reported mainly in millipedes, it has also been found in other invertebrate hosts, including the larvae of a scarabaeid beetle, wood-burrowing cockroach, and earthworms [1, 30, 31, 34, 46, 58, 63]. Because almost all studies thus far have been nematode isolation records and taxonomical descriptions, the host–parasite relationship for each species has not yet been clarified, but the broad host range is a clear characteristic of these genera.

The family Thelastomatidae has also been isolated from many invertebrates; however, overwhelmingly, a large number of species has been found in Blattodea hosts [2, 52, 53, 64]. From our field surveys and previous studies, the host specificity of nematodes belonging to the family Thelastomatidae appeared high [13, 47, 48, 52, 53, 64]. Yet, the same nematode species have been isolated from different cockroach species [35, 54, 62]. We showed, experimentally, that *Leidynema appendiculatum*, a thelastomatid parasitic nematode, was capable of infecting five cockroach species from three families in two suborders [54]. While ecological interactions between host and parasitic nematodes determine the host range, a broad host range might still be possible in Thelastomatoidea.

Parasitic nematodes in Thelastomatoidea are thought to share a simple infection cycle; nematode eggs are laid by adult females, deposited within their host’s feces, and then released outside the host’s body; ingestion of the eggs by new host individuals leads to infection [3, 18, 53]. When eggs were collected from adult females of *T. claudiae* and incubated in a PBS buffer, they developed until the infection stage but never hatched (data not shown). Such characteristics of embryogenesis are shared with the cockroach-parasitic nematode family Thelastomatidae. Because it is necessary for the eggs of *T. claudiae* to exit the host body once to generate the next developmental stage, infection prevalence might have been lower (Figs. 3A, 4, 5) and affected by the season (Fig. 3C) when compared with those of Rhigonematoidea. In addition, *T. claudiae* has a population structure similar to that of the cockroach-parasitic nematodes, with one adult male and a few adult females being present in the host [52, 54, 74]. These properties are also frequently observed in Thelastomatoidea.

Since infection of the two parasitic nematodes Rhigonematoidea and Thelastomatoidea in xystodesmid millipedes in Japan is universal, as far as we have investigated, the relationship between host millipedes and parasitic nematodes appears not to be pathogenic; rather, it is commensalism (obligatory for nematode, host is not affected) or parasitism (obligatory for nematode, host is inhibited; [15]. Only a few studies have shown the effects of these parasitic nematodes on their invertebrate hosts (e.g., [71, 72]), and they have generally been found to be harmless [3, 52, 54]. The composition of cockroach gut microbiomes is influenced by parasitic nematode species [71, 72], suggesting direct or indirect effects of nematodes on the host. As the parasitic nematodes in our study are all obligatory parasites, and culturing methods of their host millipedes have not yet been established, laboratory experiments controlling infection conditions are not possible. In addition, Thelastomatoidea was not eliminated by Rhigonematoidea when co-infected (Figs. 3, 7). From our ecological, parasitological, and phylogenetic studies, we hypothesized that some ancestors of cockroach-parasitic nematodes might have changed their host to the Passalidae beetle at an early stage, and become the family Hystrignathidae. Similarly, another ancestor might have changed its host to the millipede and become the family Travassosinematidae and *Cephalobellus* (Fig. 8C). Their properties might allow Thelastomatoidea nematodes to use the millipede as a host and also to tolerate co-infection with Rhigonematoidea. The exchange of parasitic nematode between hosts living in the same ecological niches could occur (for example, from cockroaches to millipedes), and new parasitic nematodes may have evolved in millipedes after reproductive isolation. This parasitic nematode might also have been isolated from other invertebrate hosts with similar ecology. Speciation of nematodes could also result from genetic isolation, which is largely influenced by their associated animals and plants [44].

Interestingly, all of the millipedes studied in this work tended to be infested with the same species, *T. claudiae*. These millipedes were mainly sampled from forests where the planted Japanese cedar *Cryptomeria japonica* was dominant (Table 1). This species might have spread as a result of artificial planting and, because of its low host specificity, *T. claudiae* could have established relationships with local millipedes. This hypothesis could be clarified using molecular markers, such as the whole mitochondrial genome or ITS regions, which reflect intraspecific polymorphisms.

## Conclusions

In this study, we found parasitic nematodes of the two superfamilies Rhigonematoidea and Thelastomatoidea, commonly co-infecting xystodesmid millipedes. Both superfamilies were in the suborder Spirurina, but in apparently different infraorders. We found that the infection prevalence and densitiy of Thelastomatoidea were lower than those of Rhigonematoidea, which reflects the difference in infection mechanism. However, the two nematode superfamilies Rhigonematoidea and Thelastomatoidea were not in a competitive relationship, and co-infected all millipede hosts. Our field study shows an example of the complex symbiosis among parasitic nematodes and between hosts and parasites, established throughout evolution.

## Methods

### Field collection of millipedes

Adult xystodesmid millipedes were collected from seven locations in Japan (Table 1). All millipedes were manually collected and maintained at low temperatures until dissection. Before dissection, all millipedes were first confirmed to be alive, and then the species and sexes were identified following the relevant illustration references [65, 67, 68].

### Millipede dissection and parasitic nematode observation

After body length and sex (male or female) were recorded, millipedes were dissected to determine the presence or absence of parasitic nematodes in the alimentary tract. However, once the host millipede died, the parasitic nematode also died and degraded immediately, which made their identification difficult. All the millipedes used in this experiment were alive when dissected, and the parasitic nematodes were active after being released from the host gut. Nematode species, developmental stages (adult or larvae), sex (male or female), and numbers were recorded (Additional file 9: Table S7). The prevalence % of infected millipedes among all the millipedes examined, mean density (the mean number of nematodes per infected host), and bootstrap confidence interval of each nematode species and stage (adult male, adult female, and larva) were calculated using Quantitative Parasitology 3.0 [10, 59].

Morphological characteristics were captured using Nomarski DIC optics (Eclipse E600, Nikon, Japan) equipped with a CCD camera (VTCH1.4ICE; Visualix, Japan) as described by Ozawa and Hasegawa [54]. Molecular characterization was also performed as described by Ozawa and Hasegawa [54]. Briefly, genomic DNA was extracted from individual female nematode, and the D2D3 expansion segment of the 28S ribosomal RNA gene (D2D3 LSU) was amplified using the universal primers D2A (5-ACA AGT ACC GTG AGG GAA AGT TG-3) and D3B (5-TCG GAA GGA ACC AGC TAC TA-3) [51]. Samples were submitted to Eurofins Genomics (Tokyo, Japan) for sequencing from both strands, using the same PCR primers. Sequences were deposited in GenBank NCBI (http://www.ncbi.nlm.nih.gov/genbank/).

Pairwise comparison of percent differences (D) within each nematode group (Rhigonematoidea spp., 730 bp; Travassosinematidae spp., 704 bp; Thelastomatidae spp., 676 bp) were performed using the formula D = (M/L) × 100 [16], where M is the number of alignment positions at which the two sequences have a base in common, and L is the total number of alignment positions.

### Population analysis of the two parasitic nematodes in *P. tonominea* species complex CU

To study the effect of co-infection with *R. naylae* and *T. claudiae* on the prevalence and densities of the host, *P. tonominea* species complex CU was classified into three infection patterns: (1) all the hosts, (2) the hosts only infected with *R. naylae*, or (3) the hosts co-infected with *R. naylae* and *T. claudiae*. As no host infected with only *T. claudiae* was observed in this experiment, we compared the prevalence and density of *R. naylae* in hosts infected with *R. naylae* and those co-infected with *R. naylae* and *T. claudiae*.

In addition, to study the effects of seasonal differences on the prevalence and density of the two parasitic nematodes in the *P. tonominea* species complex CU, hosts were classified into two seasonal patterns, collected during spring (from March to June) or summer (July–October) and the prevalence and density of *R. naylae* and *T. claudiae* between the two seasons were compared.

Significant differences in nematode infection prevalence and mean densities were calculated using Fisher’s exact test and the bootstrap 2-sample t-test, respectively [59]. Differences in host body size in these infection conditions or seasonal conditions were analyzed using Tukey’s multiple comparison test followed by Bonferroni correction.

### Population analysis of the three parasitic nematodes in *Riukiaria* spp.

To study the effect of co-infection with the three parasitic nematodes, Rhigonematoidea sp. 1, *T. claudiae*, and Thelastomatidae spp. on the prevalence and density, all *Riukiaria* hosts were combined and categorized into the three infection patterns: (1) the host infected with only *T. claudiae*, (2) the host co-infected with *T. claudiae* and Thelastomatidae spp., or (3) the host infected with only Thelastomatidae spp. Instead of the high infection prevalence of all stages and sexes of Rhigonematoidea sp. 1, the prevalence of adult males and juveniles of *T. claudiae* and Thelastomatidae spp. was low, and we used data only from adult females. We compared the densities of *T. claudiae* and Thelastomatidae spp. in hosts infected with a single or two parasite species.

Furthermore, the densities of Rhigonematoidea sp. 1 females were compared between the three host conditions: (1) hosts infected with only *T. claudiae*, (2) hosts co-infected with *T. claudiae* and Thelastomatidae spp., or (3) hosts infected with only Thelastomatidae spp. Significant differences in nematode mean densities were calculated using a bootstrap 2-sample t-test [59]. Differences in host body size under these infection conditions were analyzed using Tukey’s multiple comparison test followed by Bonferroni correction. Differences in host body size under these infection conditions were analyzed using Tukey’s multiple comparison test followed by Bonferroni correction.

### Phylogenetic analysis

For the phylogenetic analysis, D2D3 LSU sequences obtained in this experiment (Table 1) and the data uploaded to the NCBI database and published in the paper were used (Additional file 8: Table S6). ClustalW multiple alignment was conducted using BioEdit version 7.2.6 [26] and sequence alignments were trimmed automatically by trimAI with default settings [11]. A phylogenetic tree of the suborder Spirurina was constructed from evolutionary distances by the neighbor-joining method using Mega 6.0 software and the Tamura-Nei model [66]. Phylogenetic trees of the Rhigonematomorpha and Thelastomatoidea were constructed from evolutionary distances by the maximum likelihood (ML) method using Mega 6.0 software [66] and the Hasegawa-Kishino-Yano model [27]. Phylogenetic robustness was inferred by bootstrap analysis using 1000 iterations [21].

## Supplementary Information

**Additional file 1: Figure S1.** Photographs of xystodesmid millipedes studied in this experiment.

**Additional file 2: Figure S2.** Average ± S.D. of host body size (mm) in infection condition (infected with only R. naylae or co-infected with R. naylae and T. claudiae) or in different seasonal conditions (spring or summer).

**Additional file 3: Table S1.** Pairwise differences % in the D2D3 sequence (730 bp) between 15 samples of Rhigonematoidea spp.

**Additional file 4: Table S2.** Pairwise differences % in the D2D3 sequence (704 bp) between 13 samples of Travassosinematidae spp.

**Additional file 5: Table S3.** Population of the two parasitic nematodes in Parafontaria millipedes.

**Additional file 6: Table S4.** Pairwise differences % in the D2D3 sequence (676 bp) between eight samples of Thelastomatidae spp.

**Additional file 7: Table S5.** Population of the parasitic nematodes in Riukiaria millipedes.

**Additional file 8: Table S6.** List of DNA sequence used for molecular phylogenetic analysis.

**Additional file 9: Table S7.** Datasheet of parasitic nematodes isolated from xystodesmid millipedes.

## Data Availability

Sequence data produced in this study are available in the NCBI (https://www.ncbi.nlm.nih.gov/) with Accession No.: MT988313-MT988378.

## Abbreviations

CU: Chubu University as a collection site
D2D3 LSU: D2D3 expansion segment of the large subunit ribosomal RNA gene
SSU: Small subunit ribosomal RNA gene
