# Supplementary material for: Symbiosis of the millipede parasitic nematodes Rhigonematoidea and Thelastomatoidea with evolutionary different origins
Source: BMC Ecol Evol. 2021 Jun 12;21:120. doi: 10.1186/s12862-021-01851-4 (PMC8199837; doi:10.1186/s12862-021-01851-4)

Parafontaria laminata CU
32.5 ± 2.7 mm (N=108)

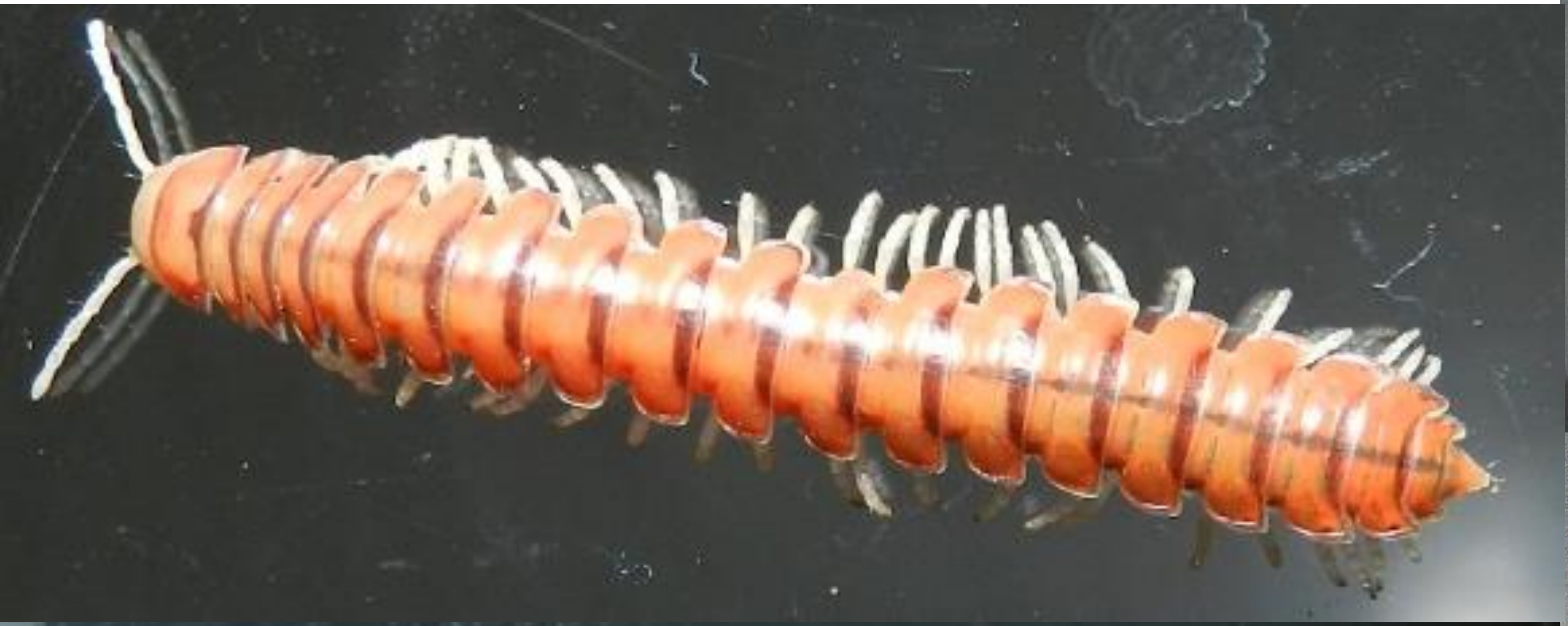

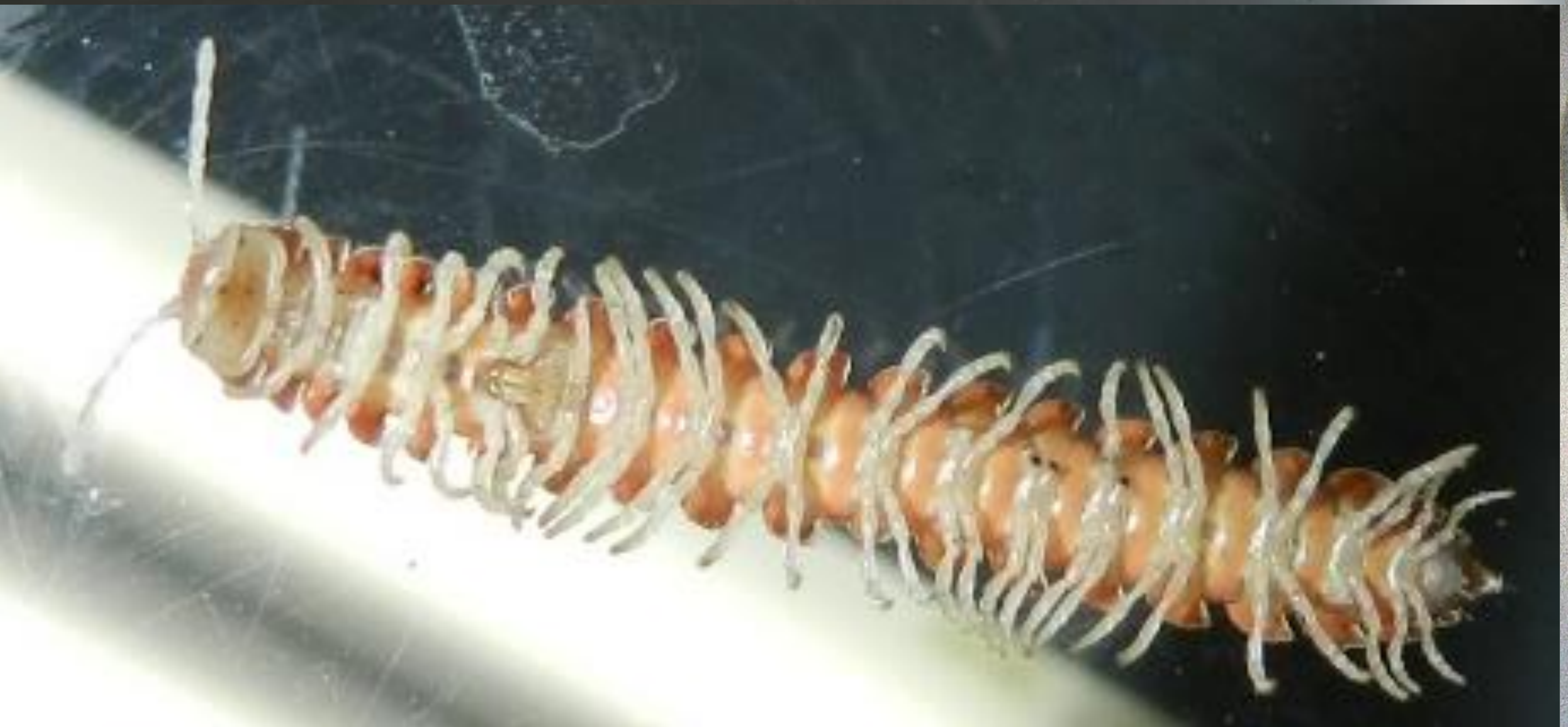

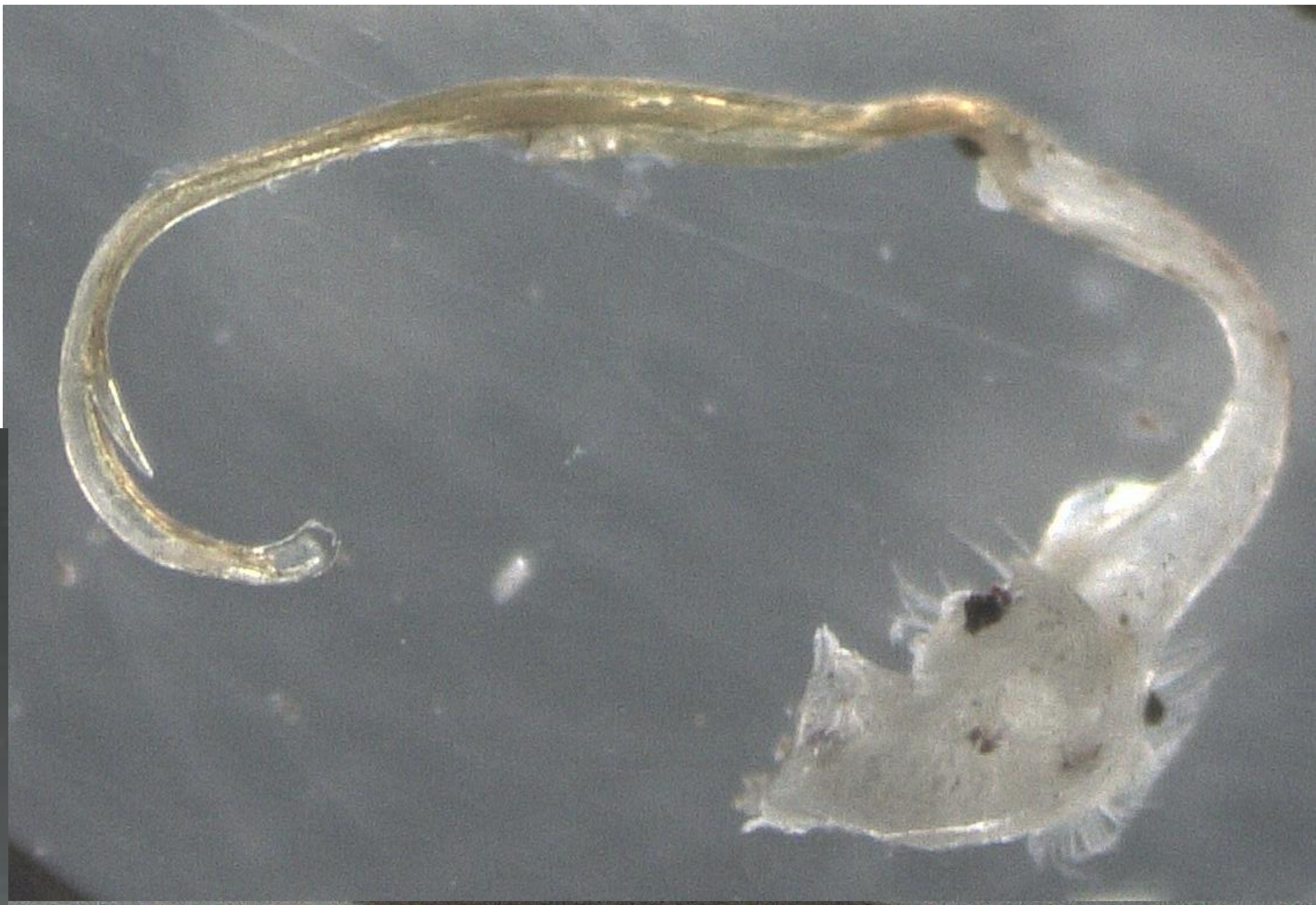

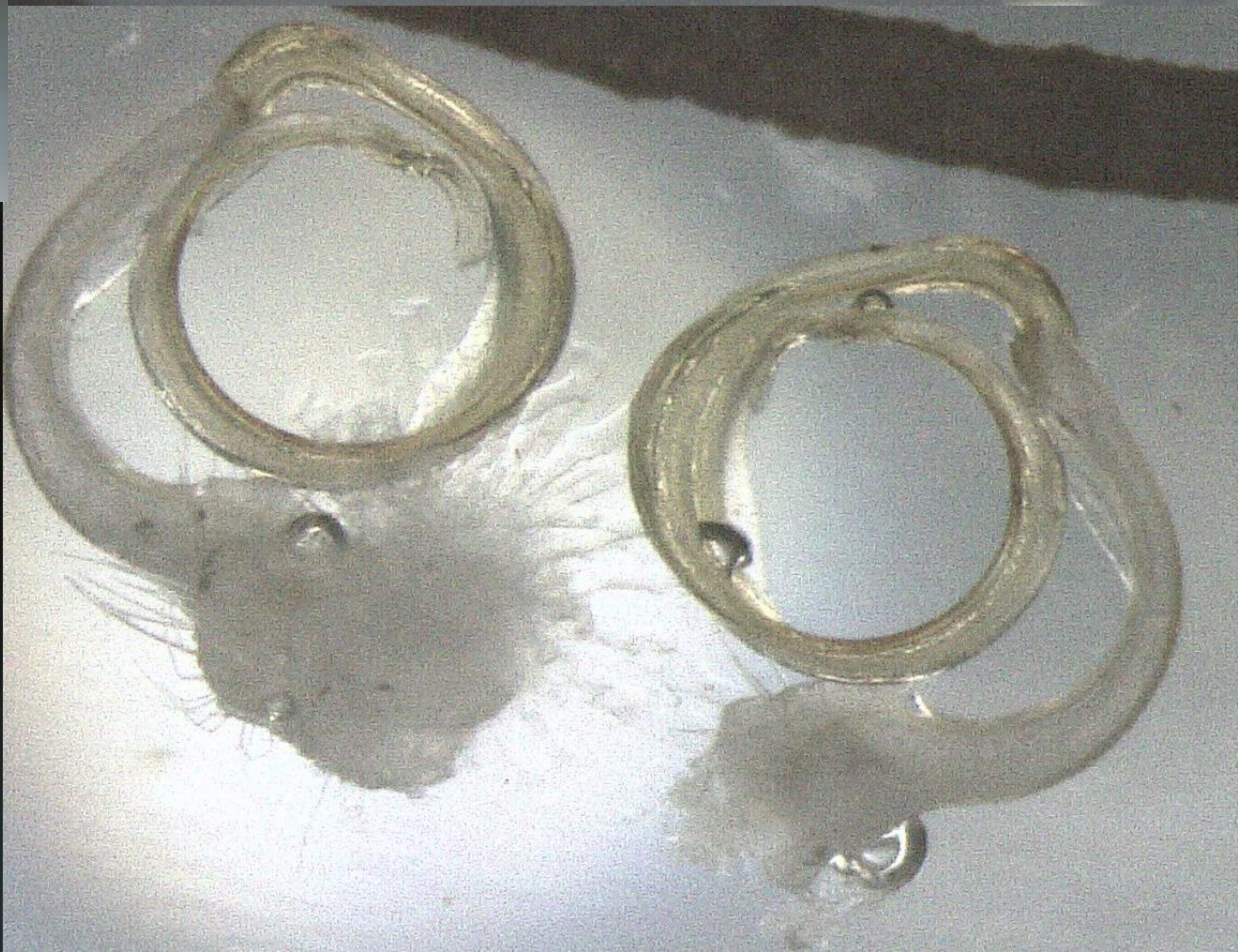

Parafontaria tonominea species complex CU

51.7 ± 3.8 mm (N=80)

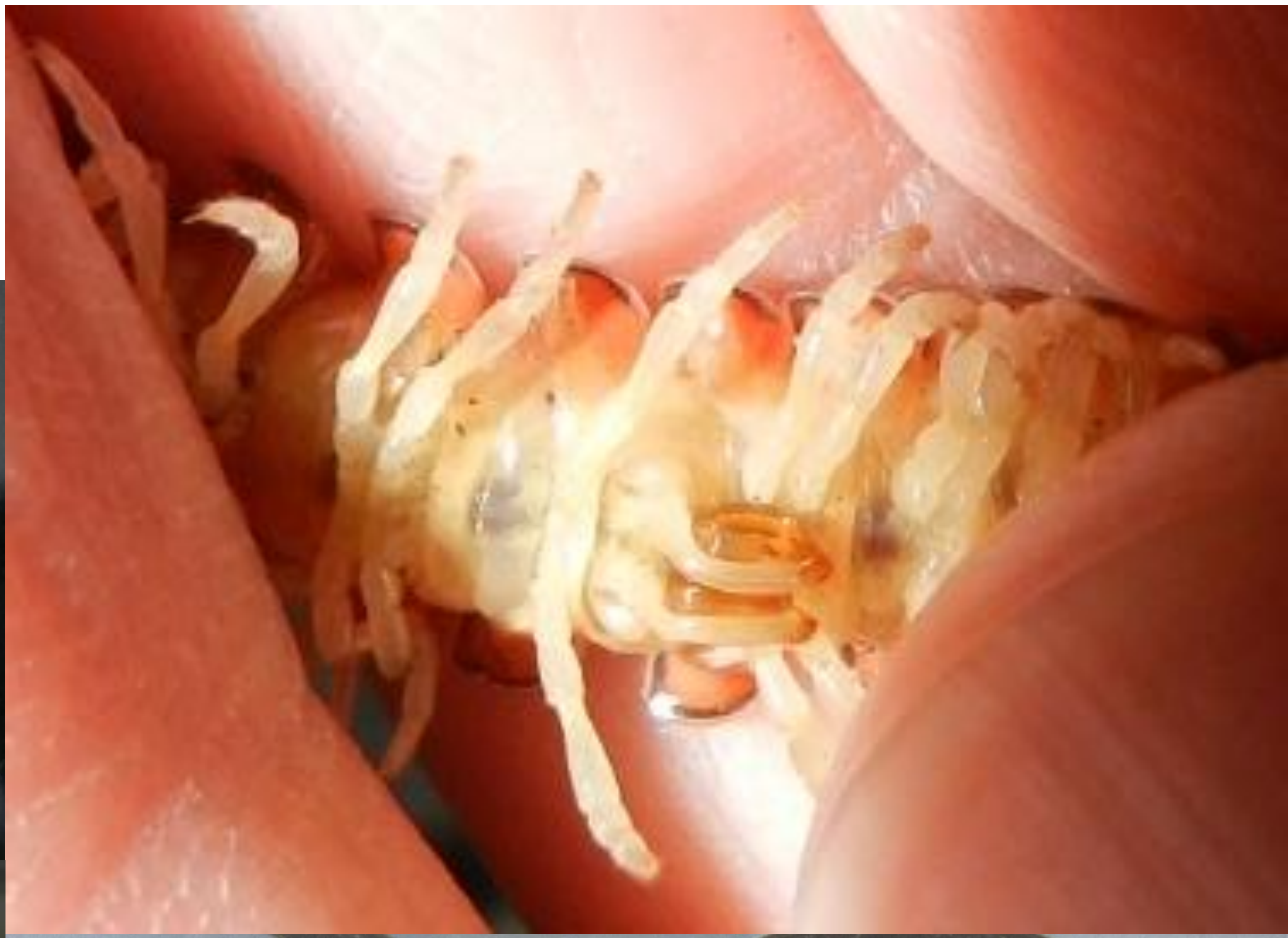

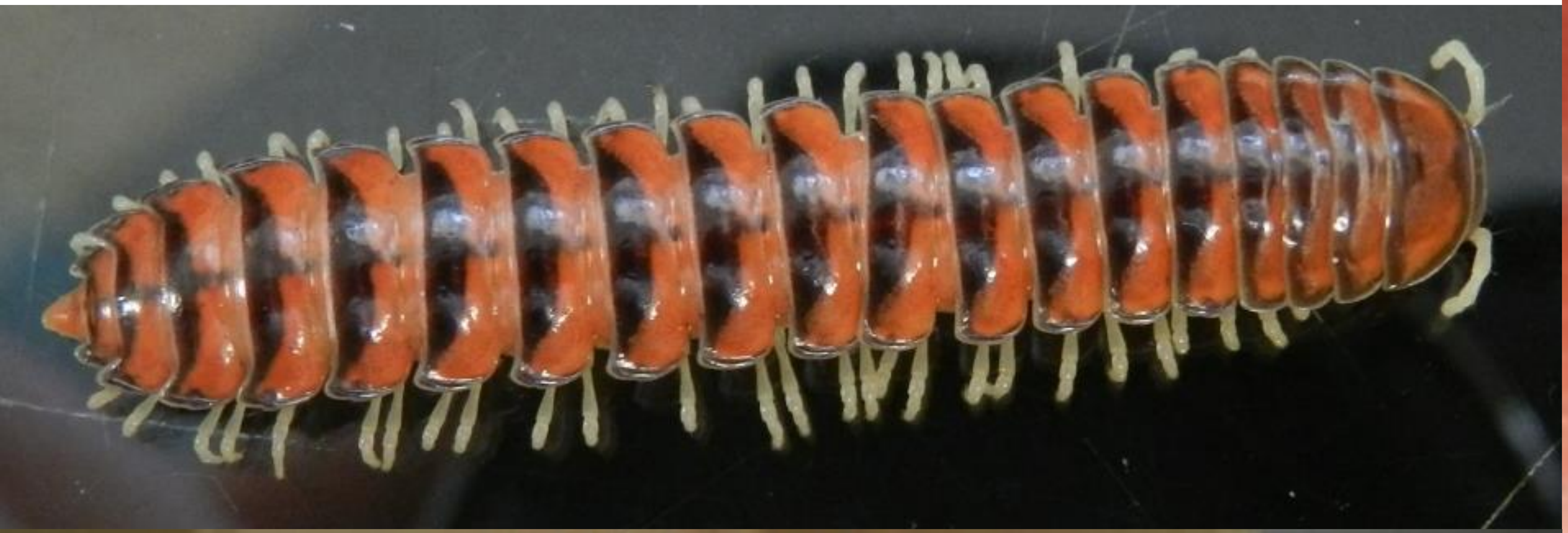

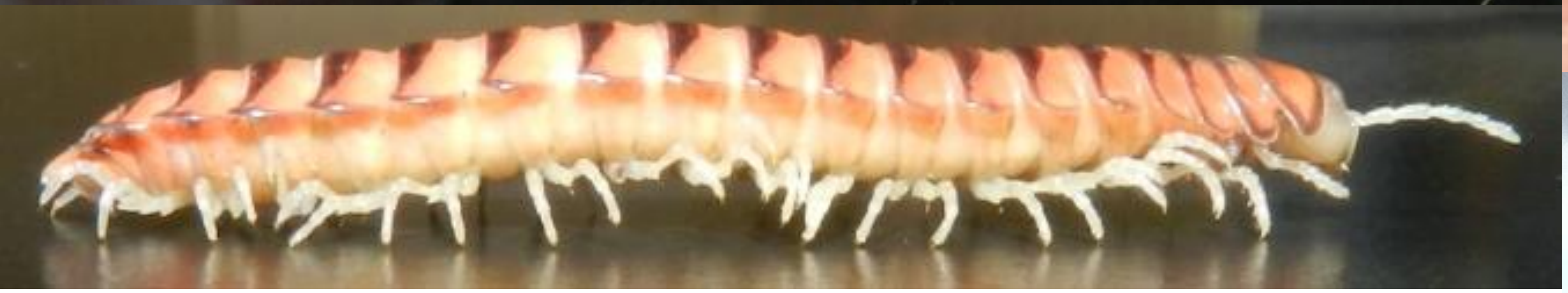

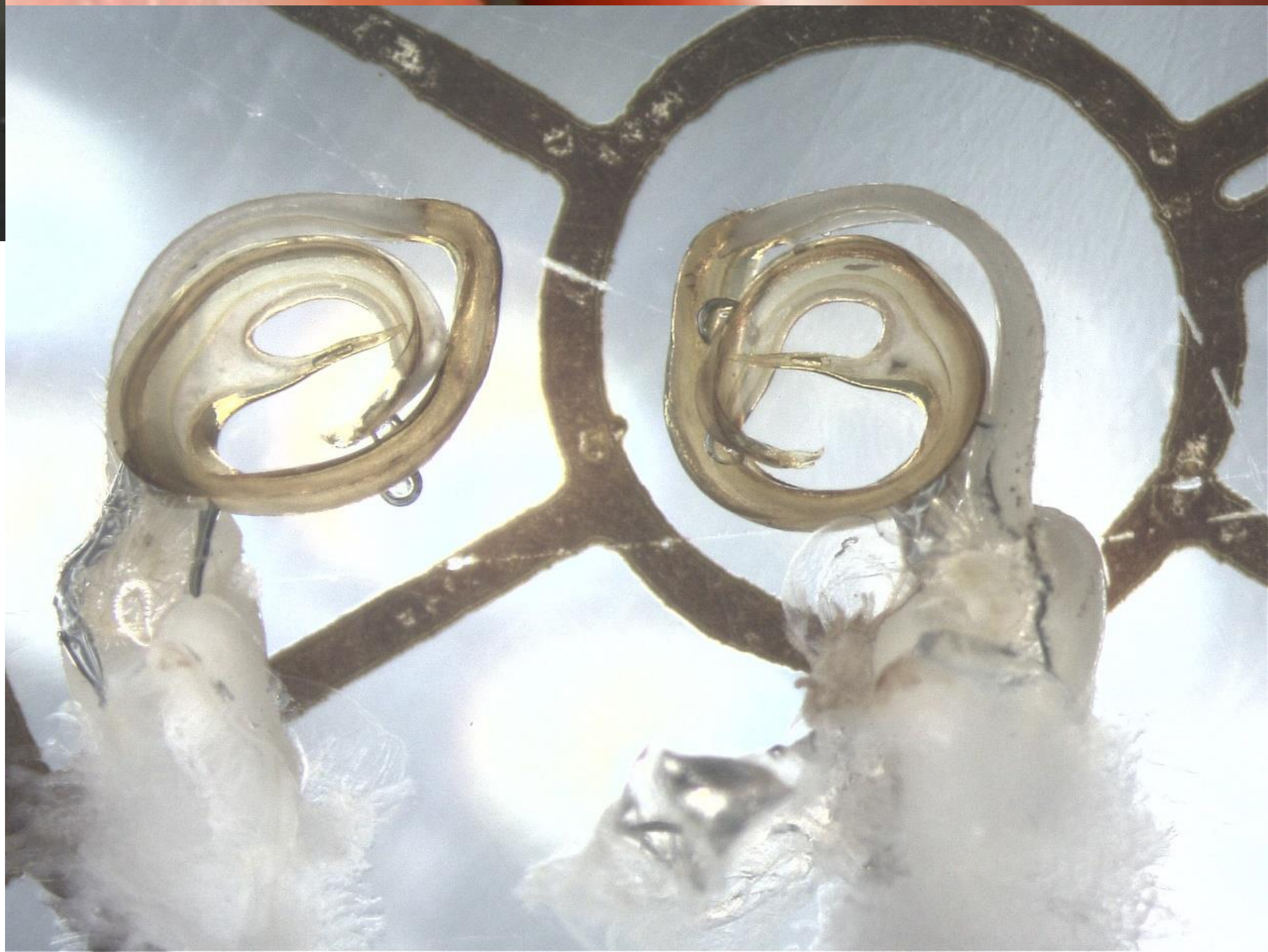

Parafontaria laminata Kinka

45.7 ± 2.8 mm (N=31)

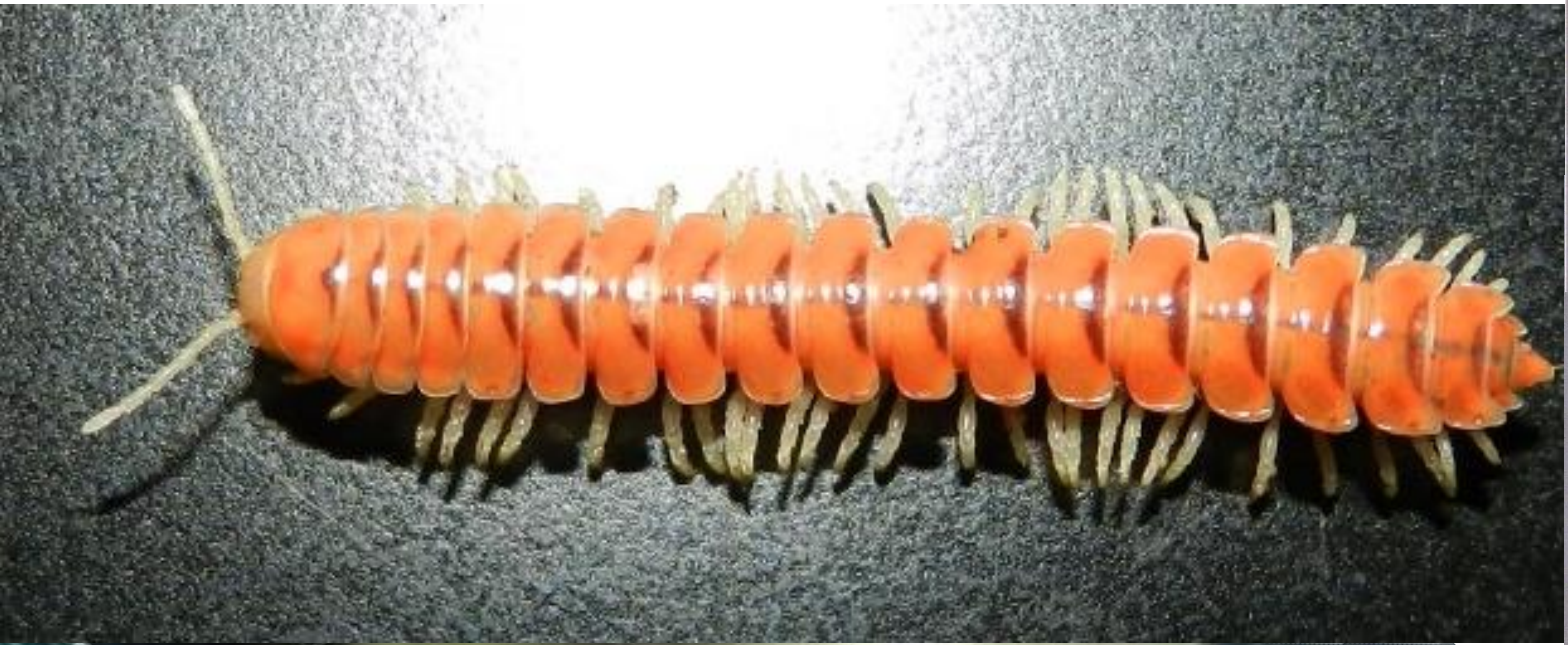

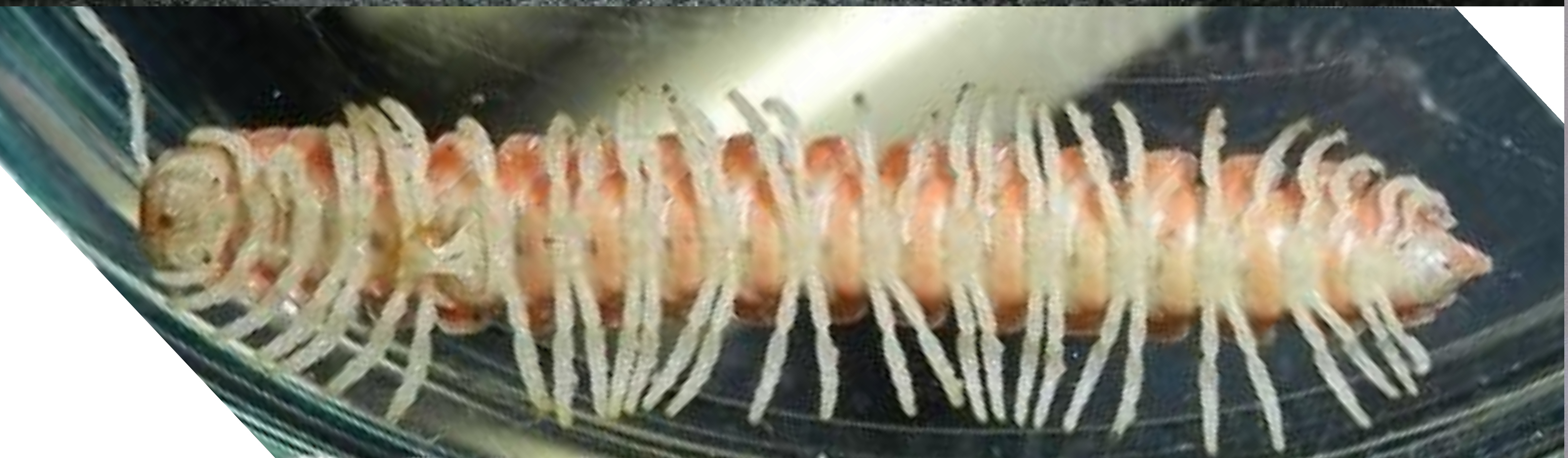

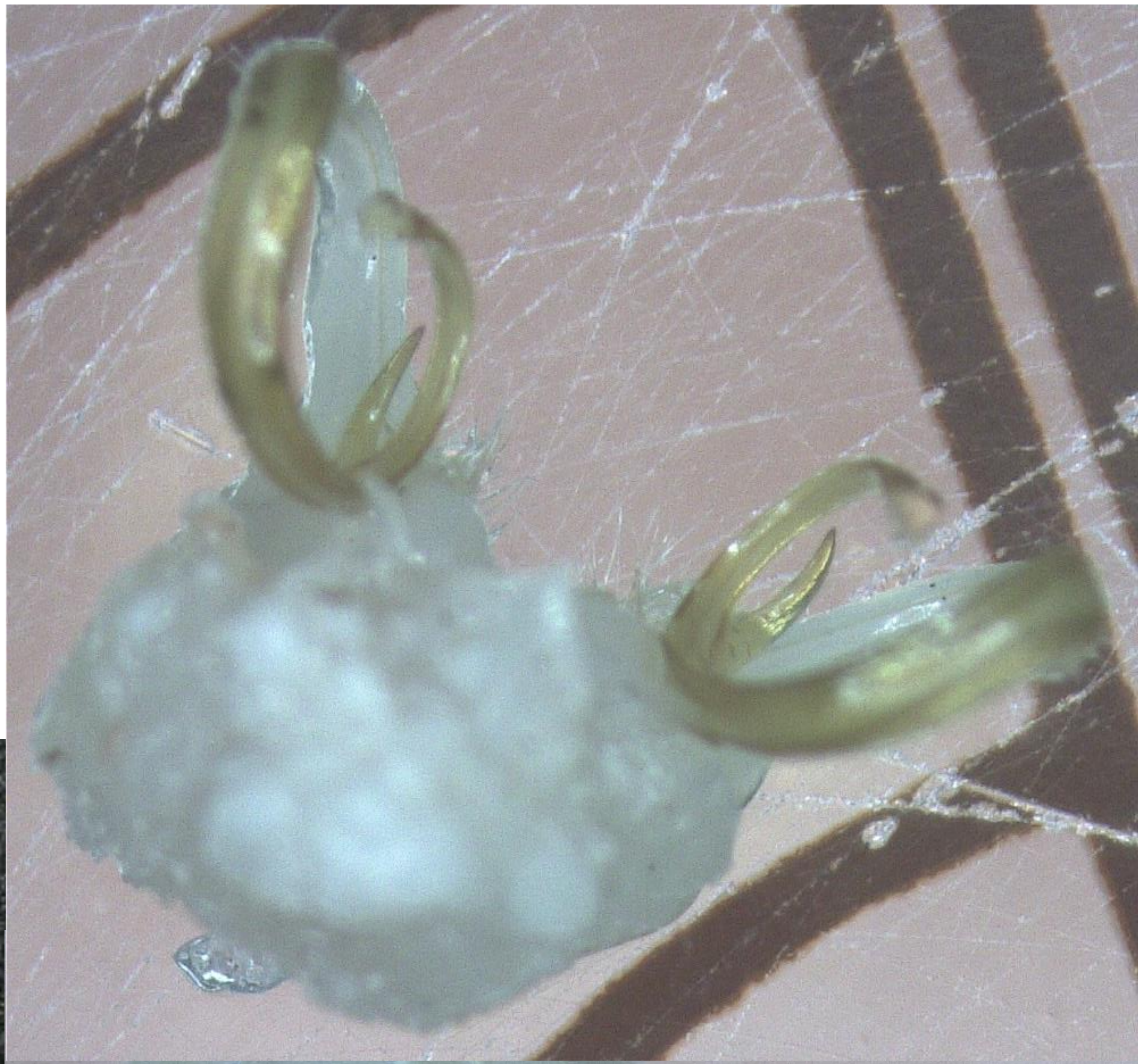

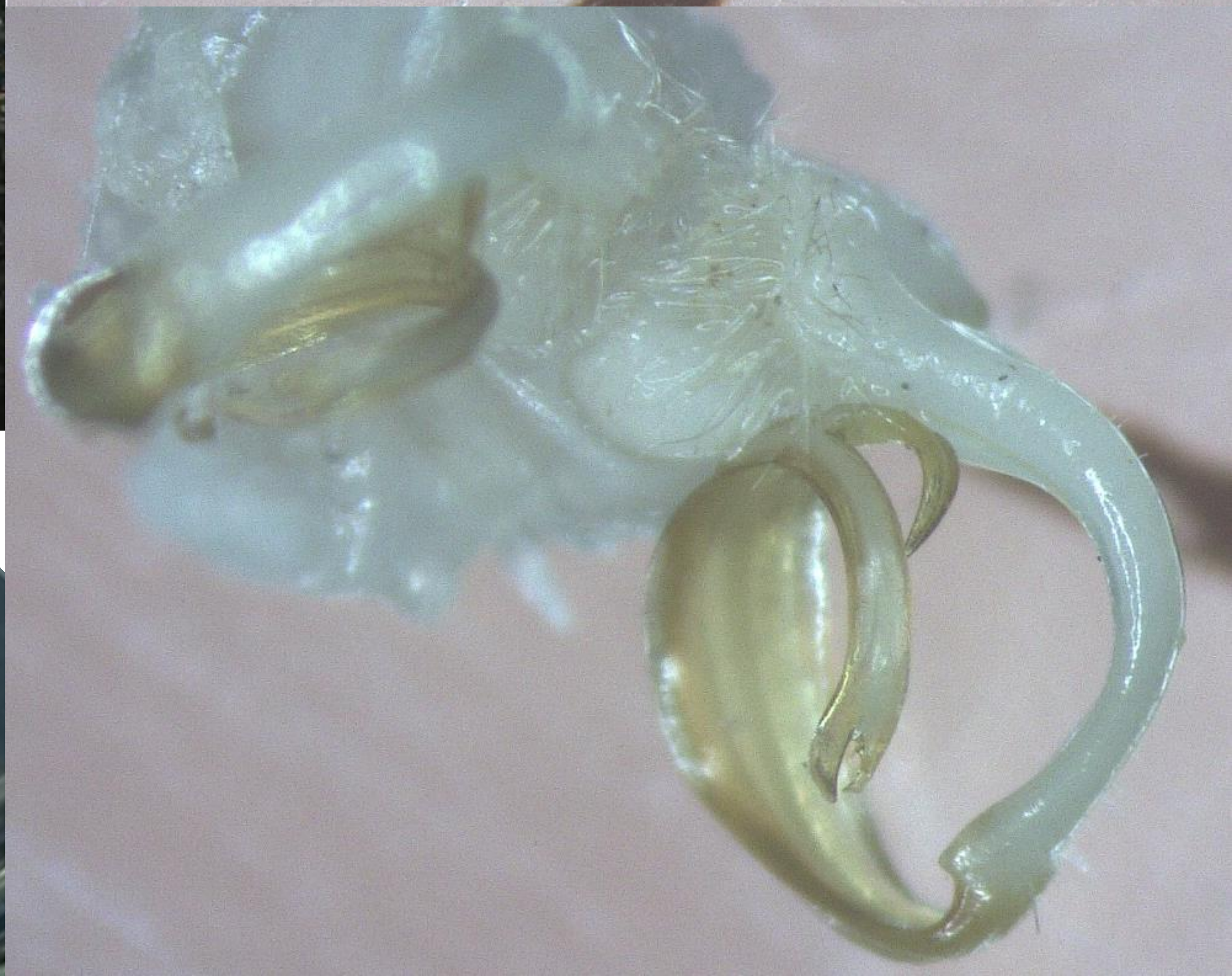

Parafontaria tonominea species complex Kinka
52.0 ± 2.7 mm (N=41)

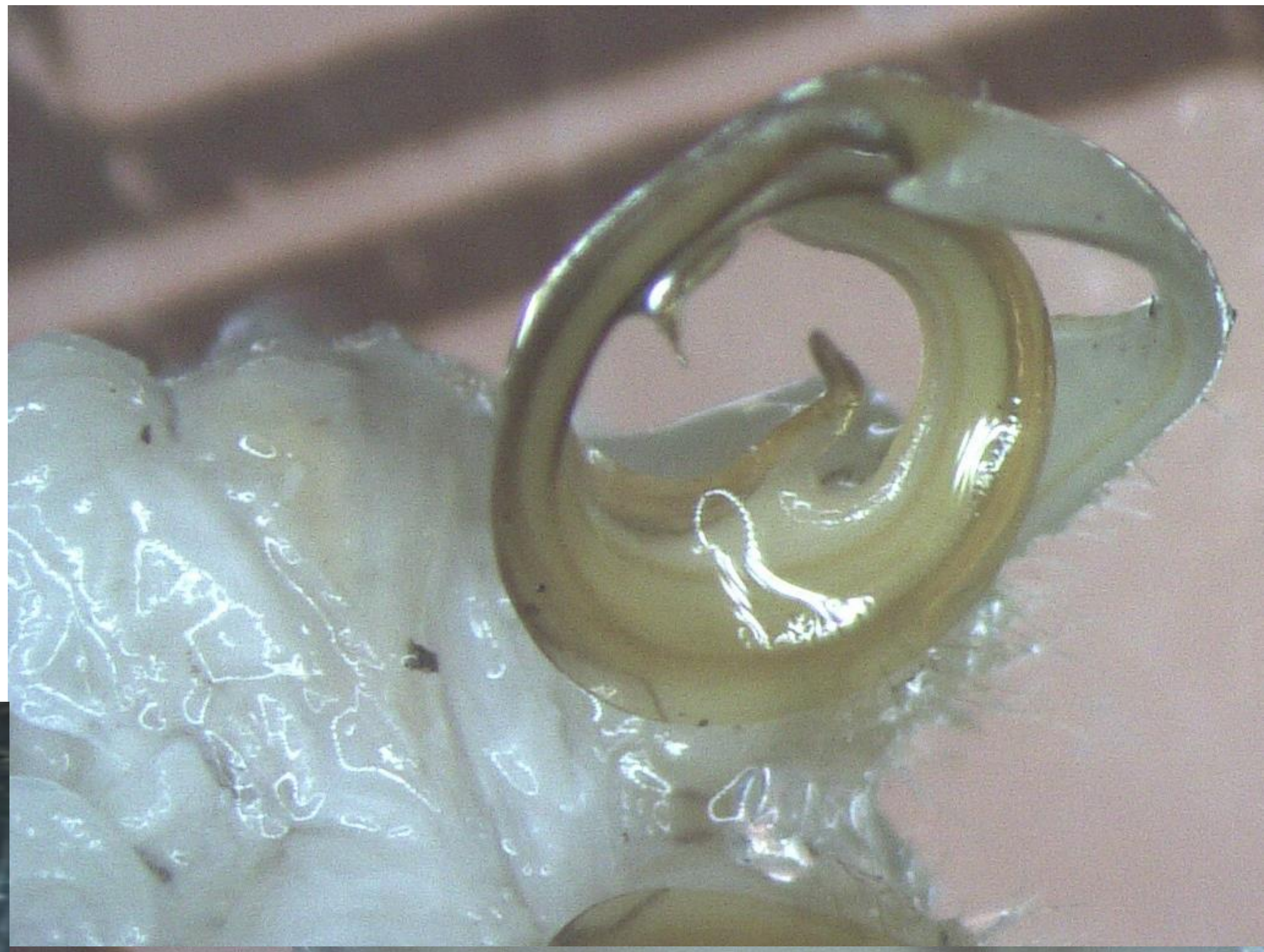

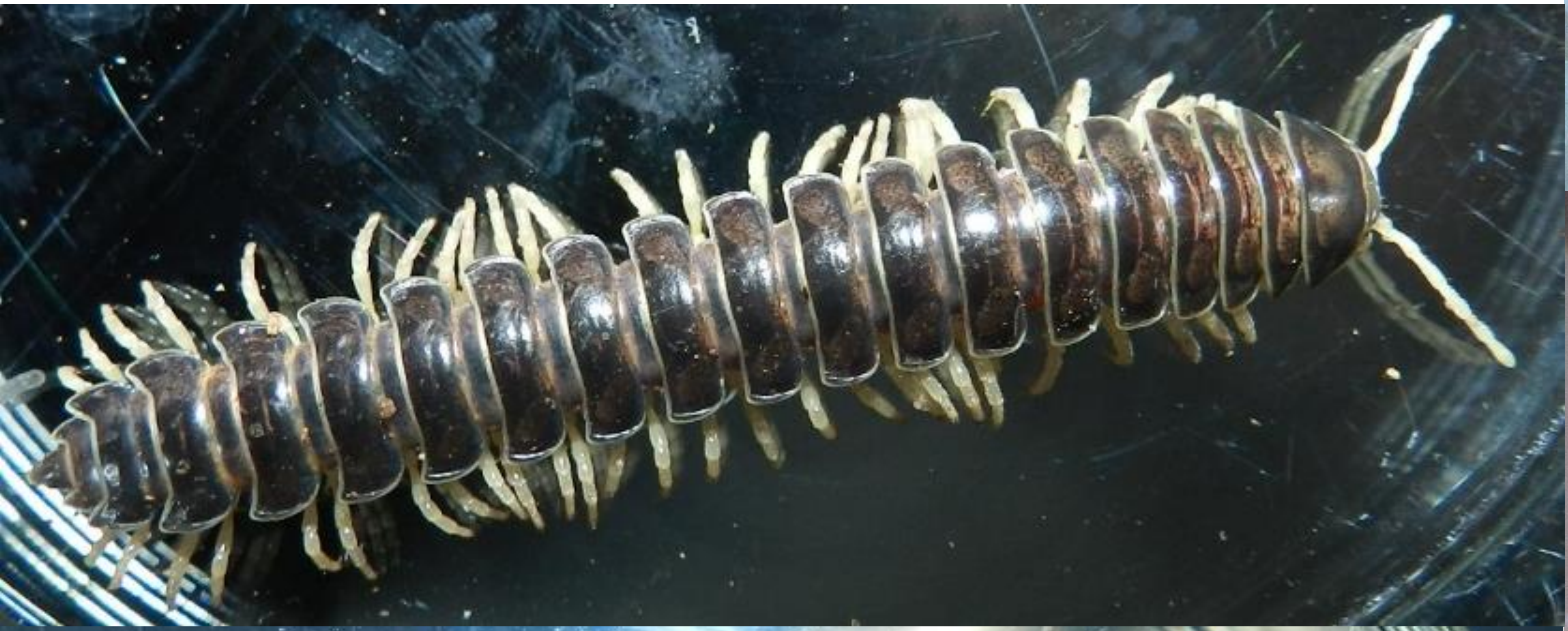

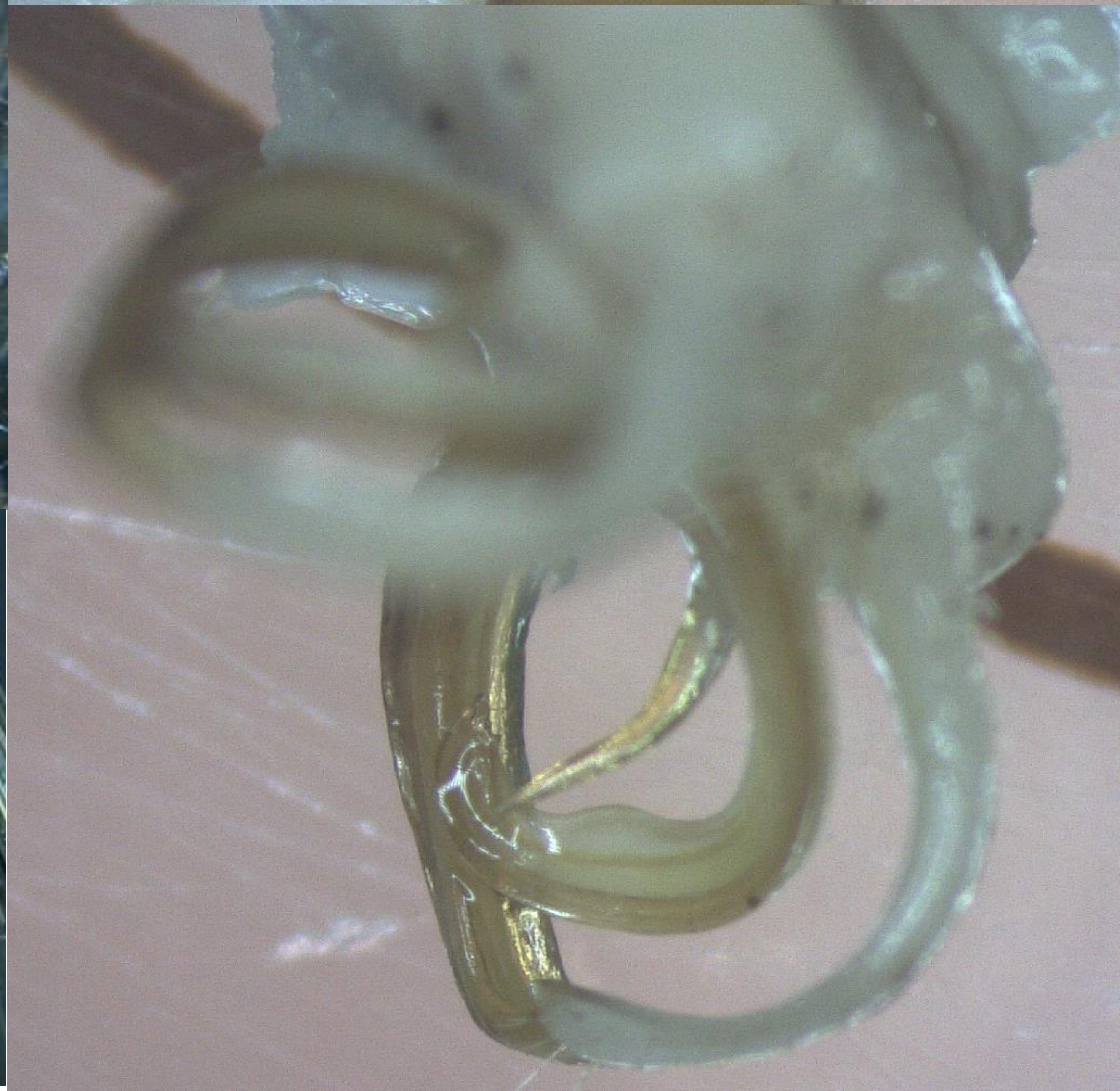

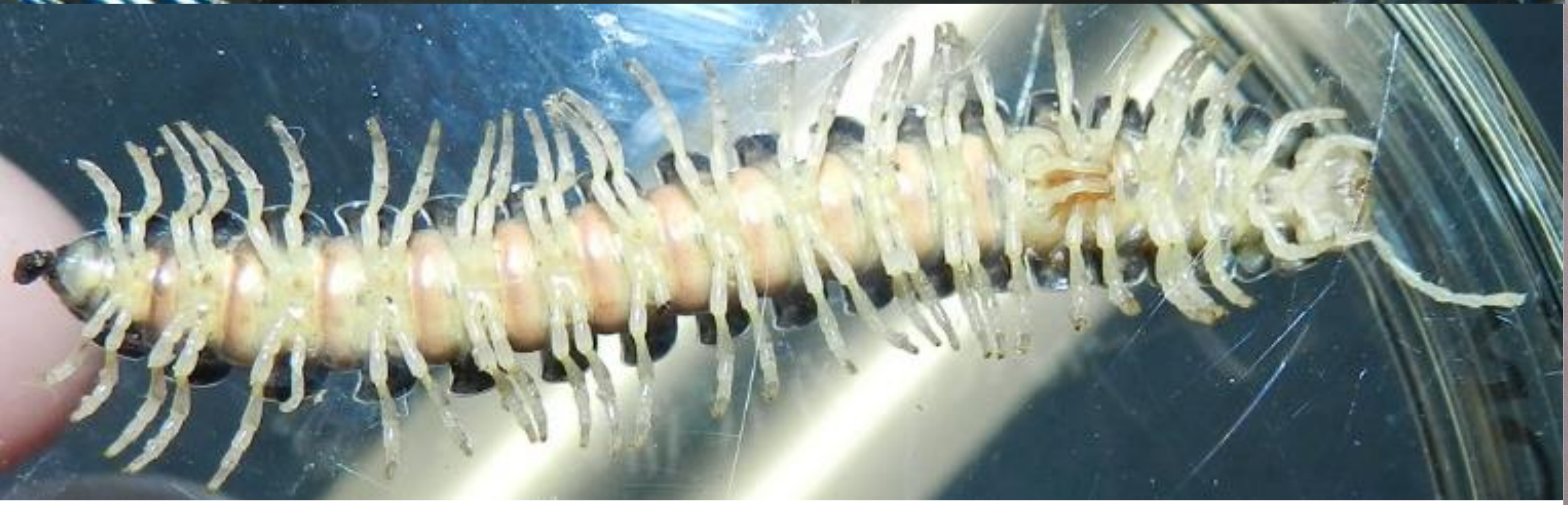

Parafontaria tonominea species complex Hyaku
52.9 ± 2.3 mm (N=22)

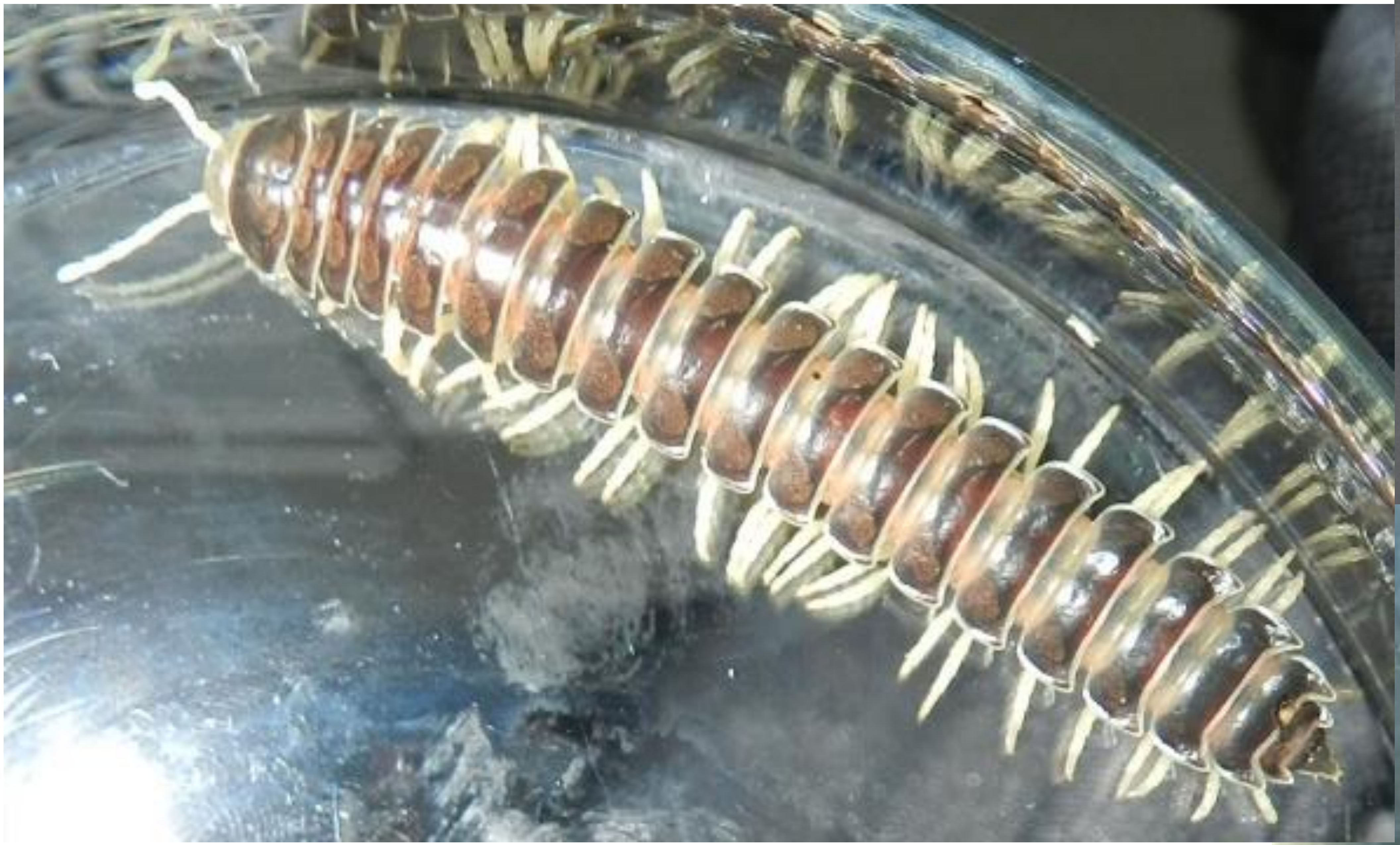

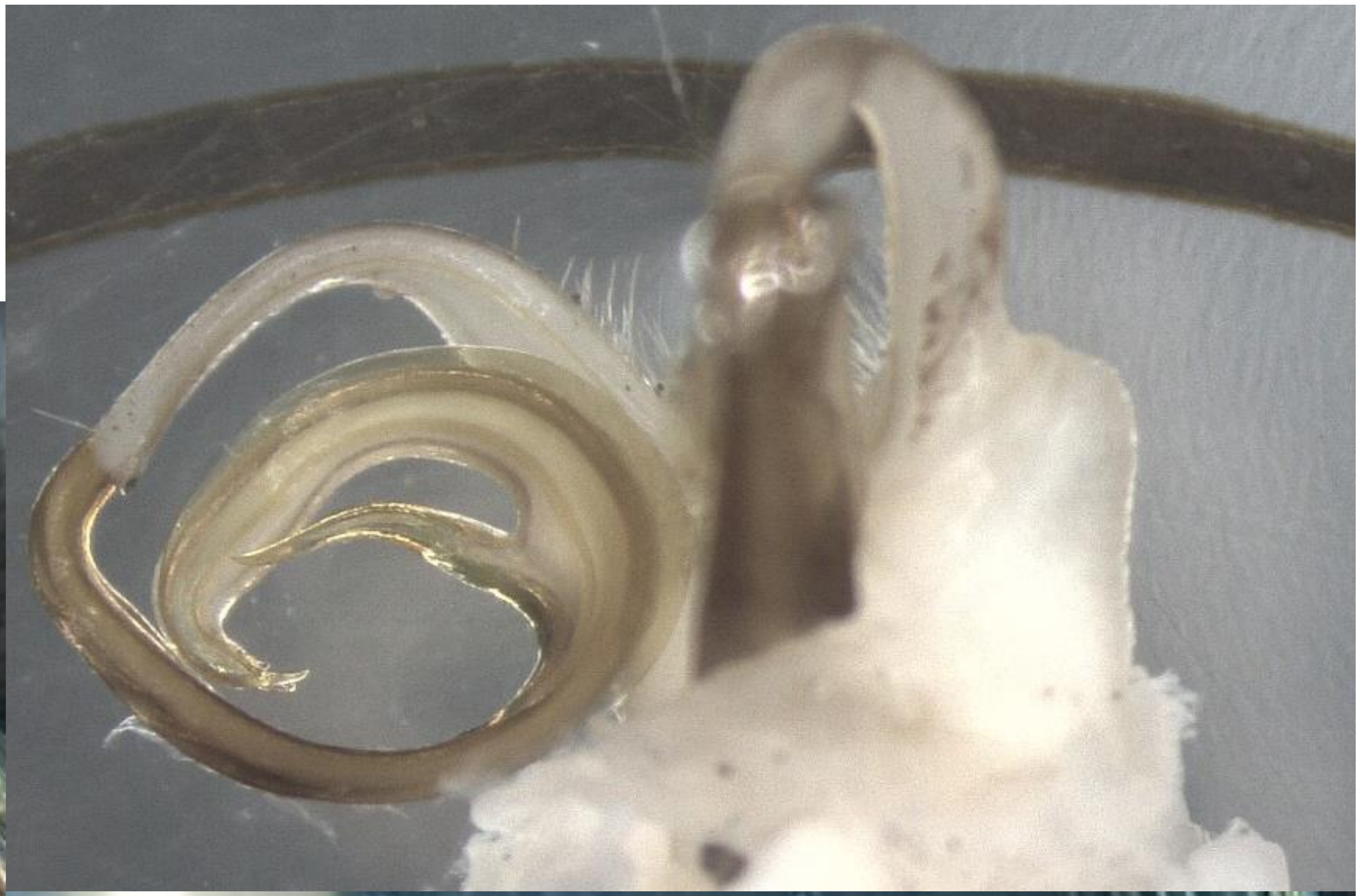

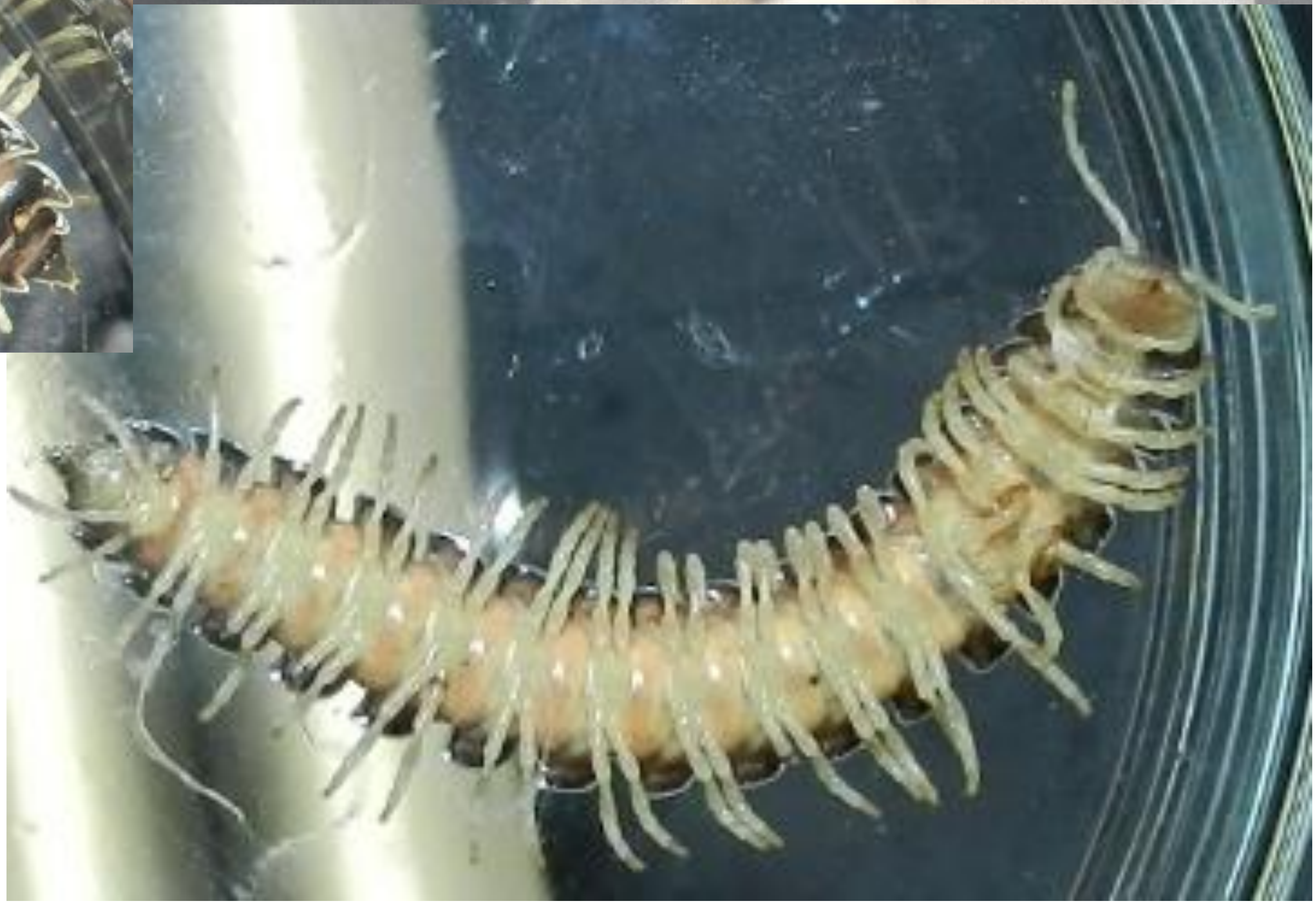

***Parafontaria tonominea* species complex Embara**
61.8 ± 2.4 mm (N=20)

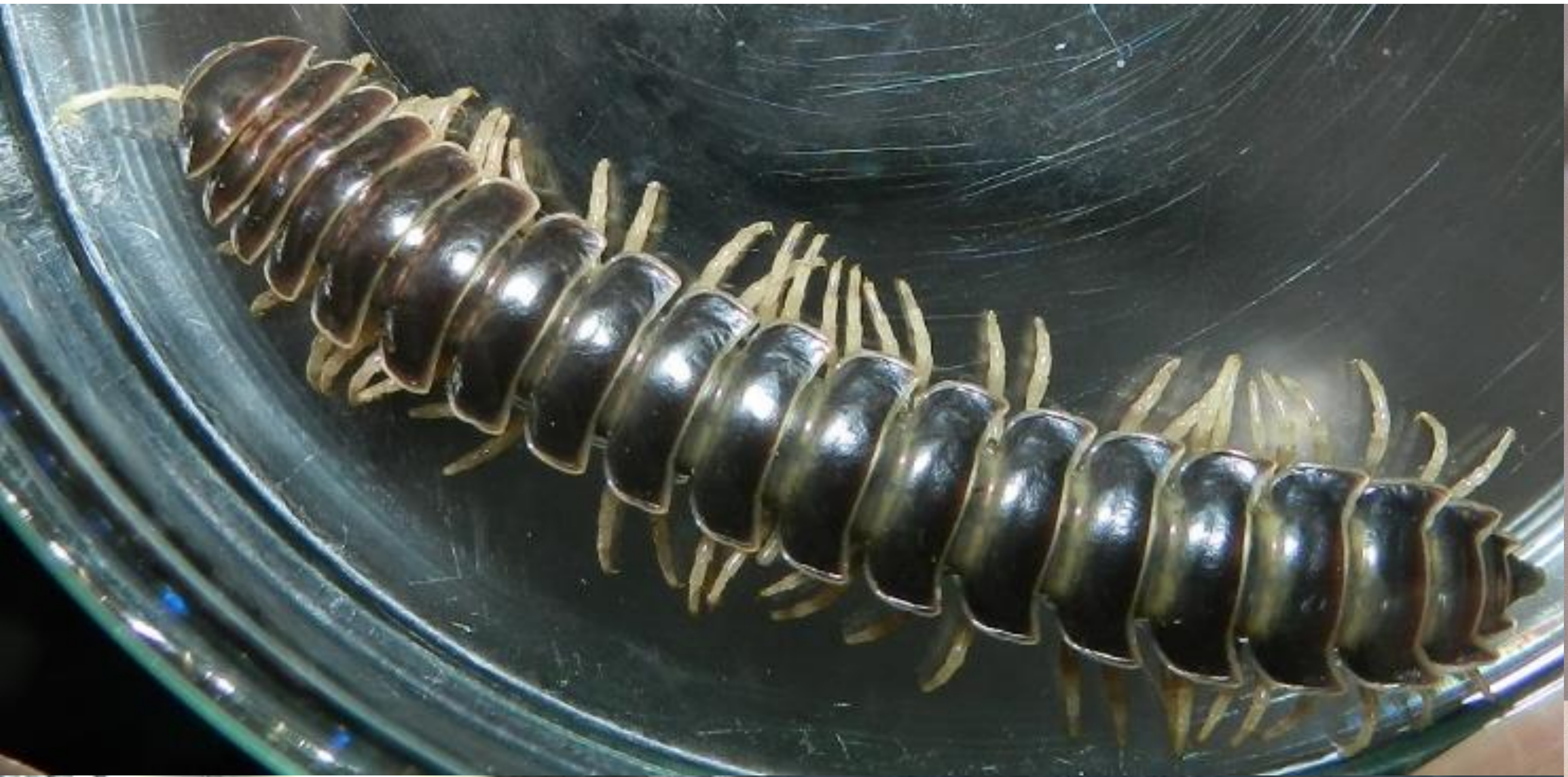

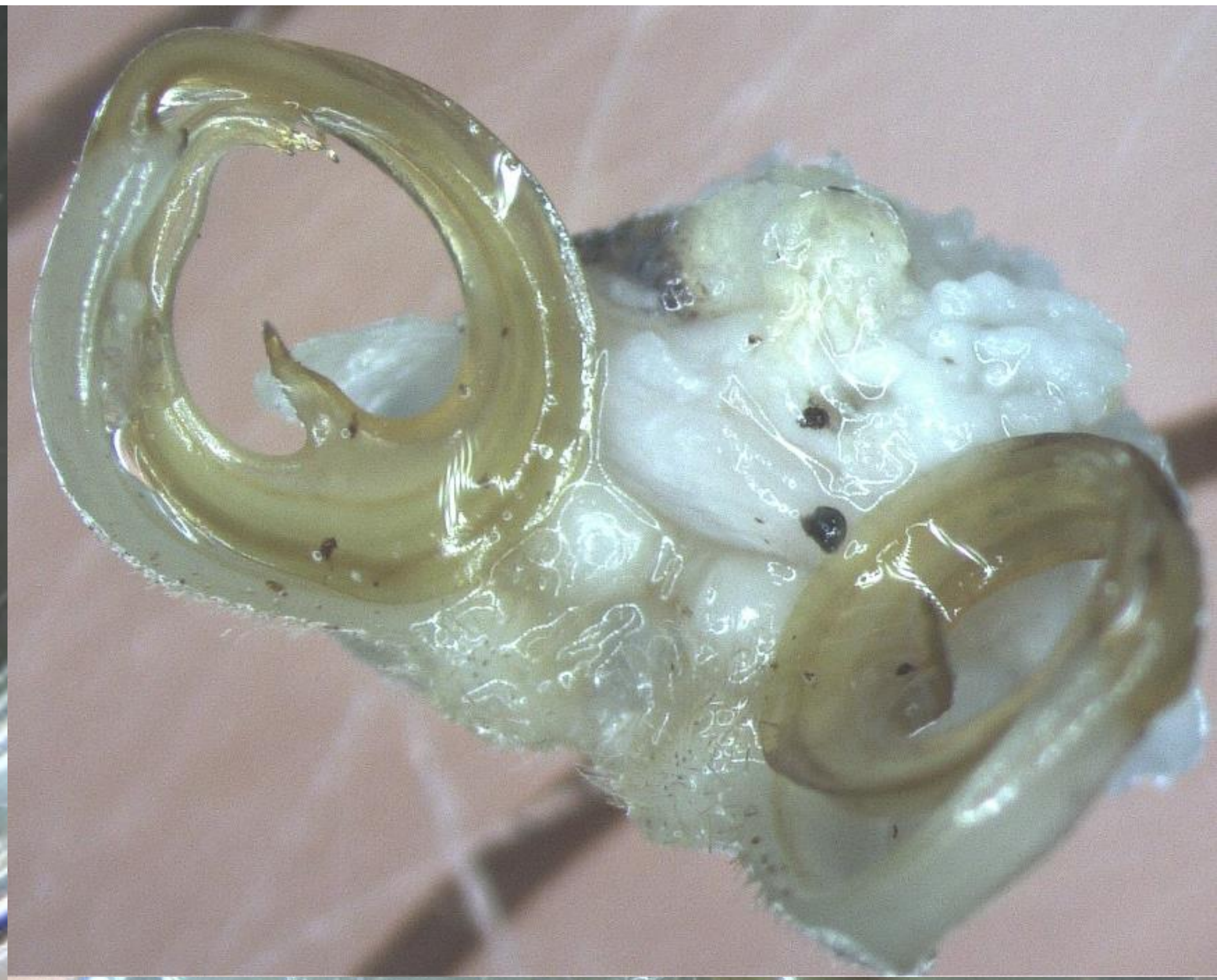

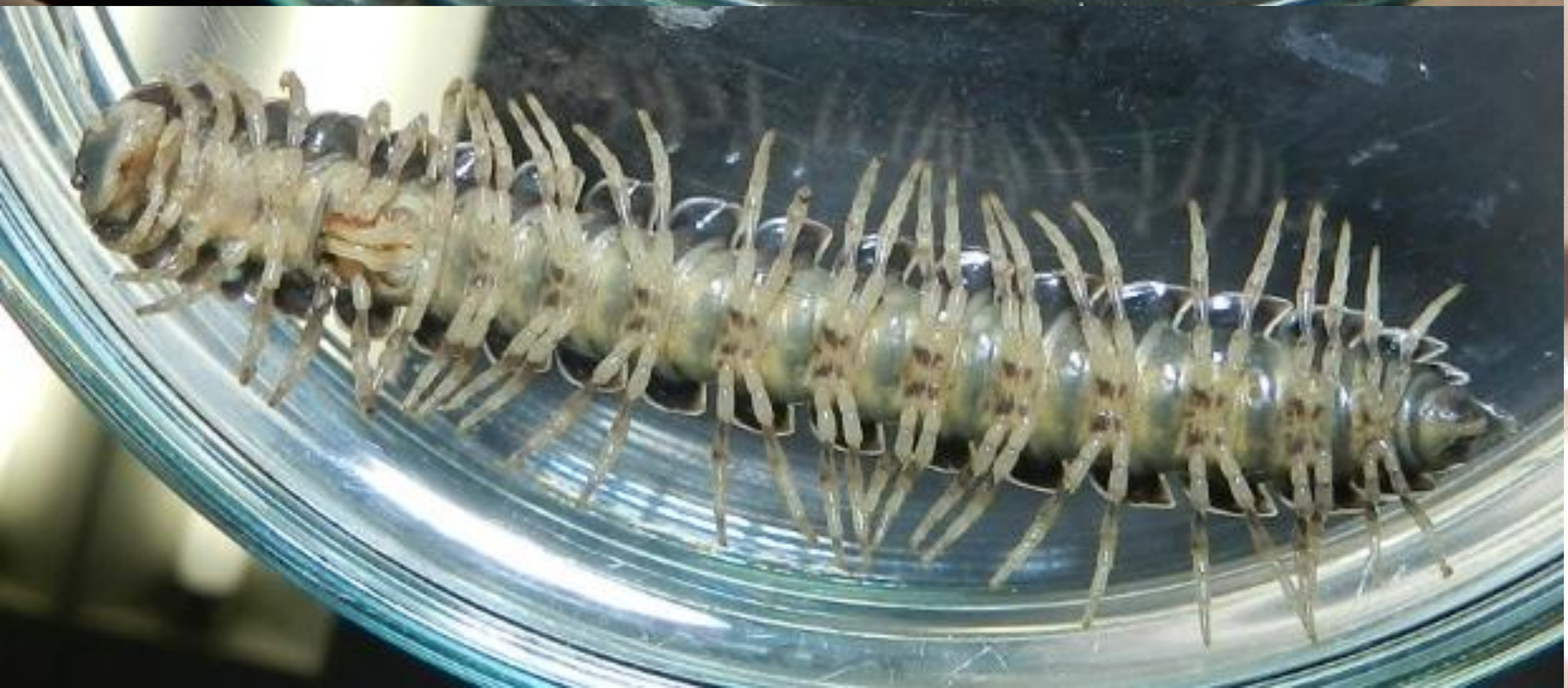

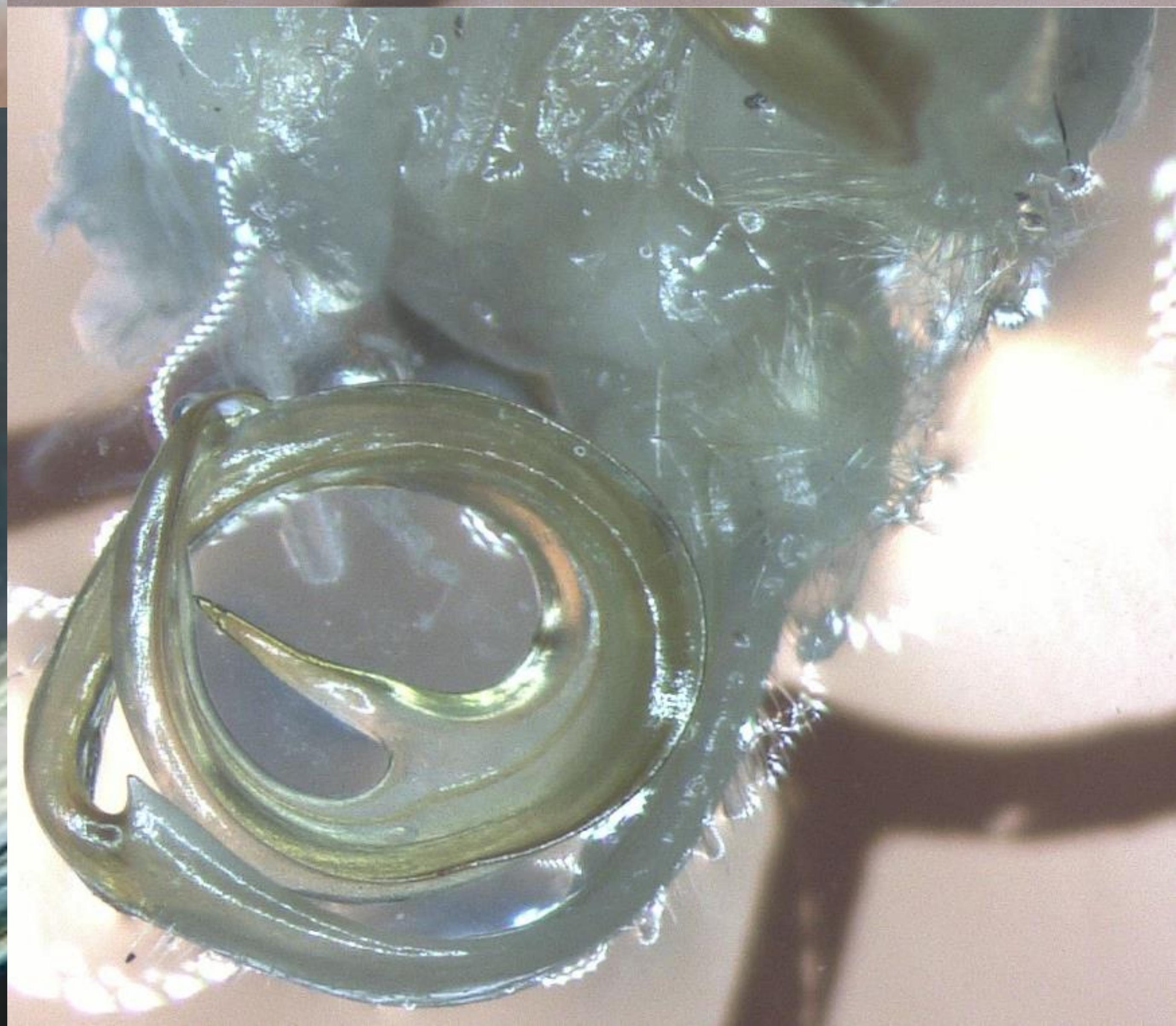

Parafontaria longa **Embara**
55.7 ± 3.4 mm (N=7)

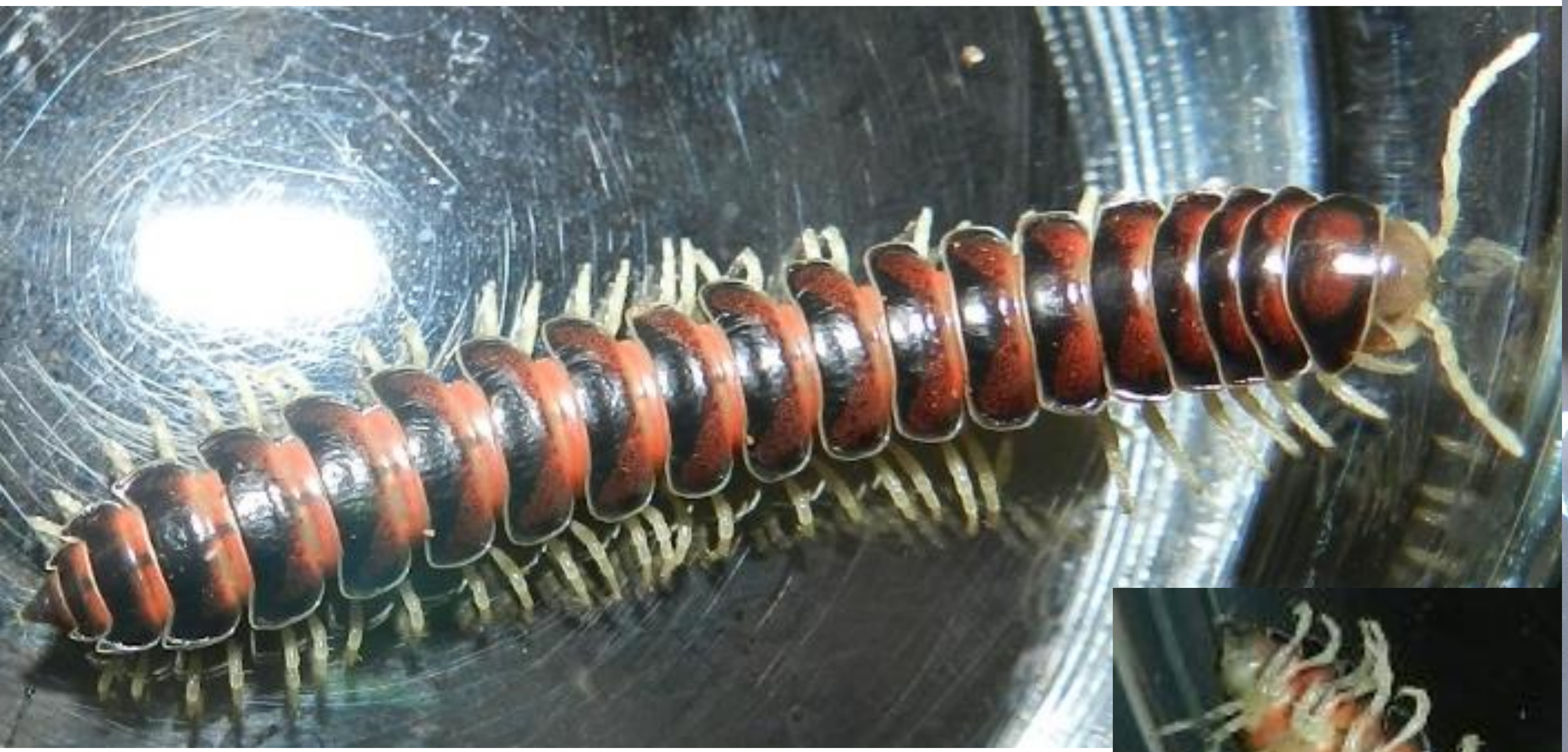

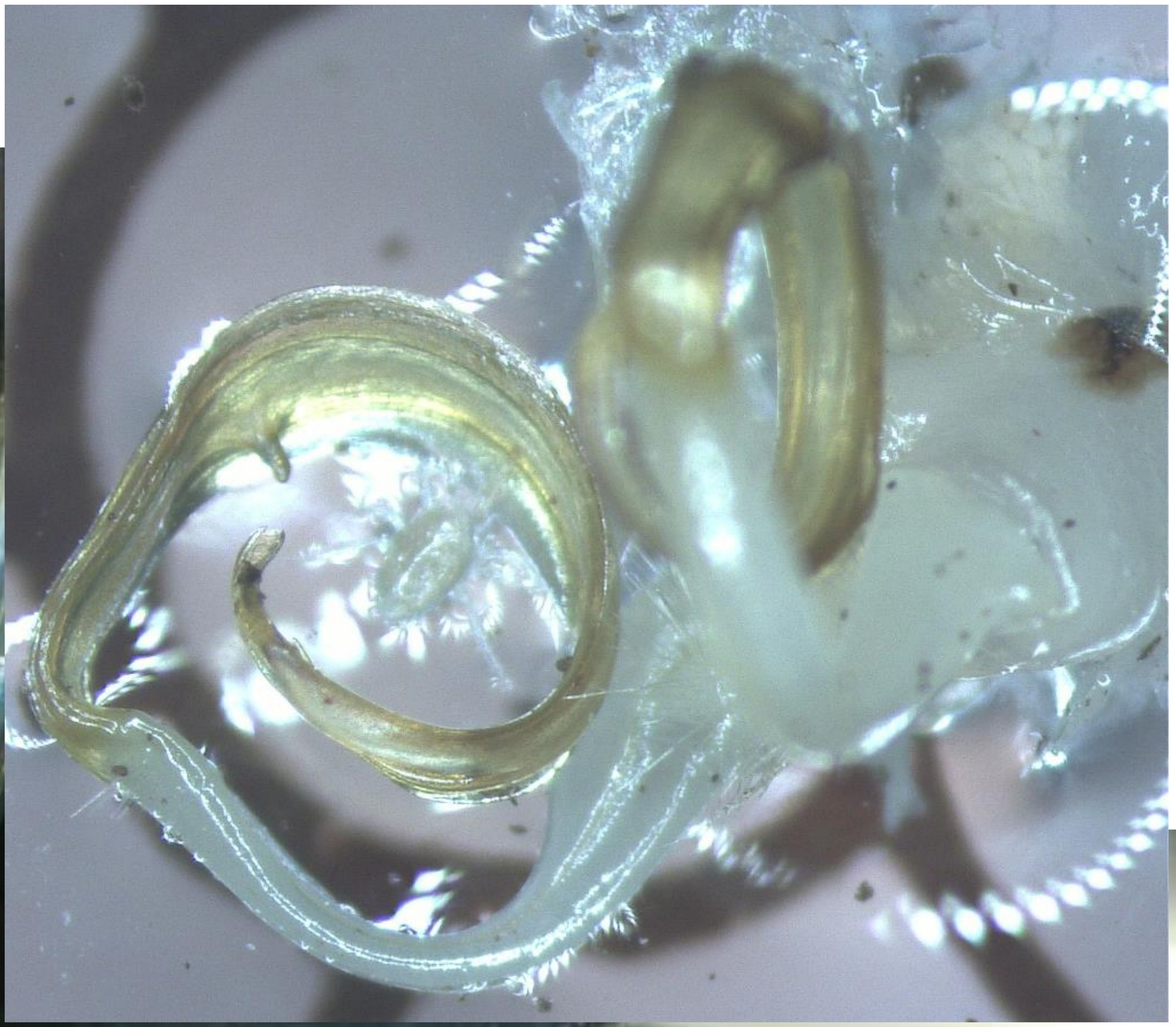

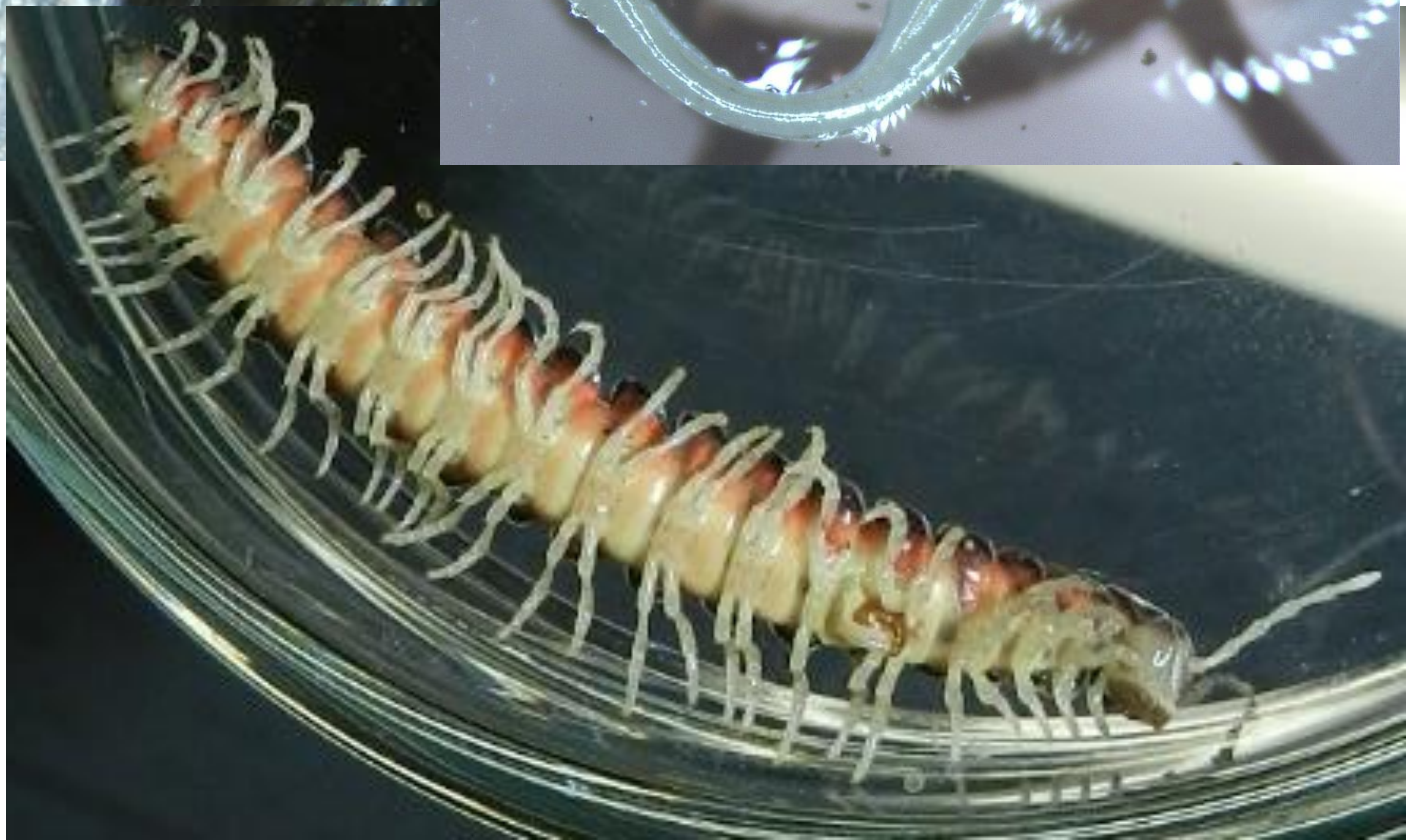

Riukiaria cornuta Yamaga
67.9 ± 3.2 mm (N=29)

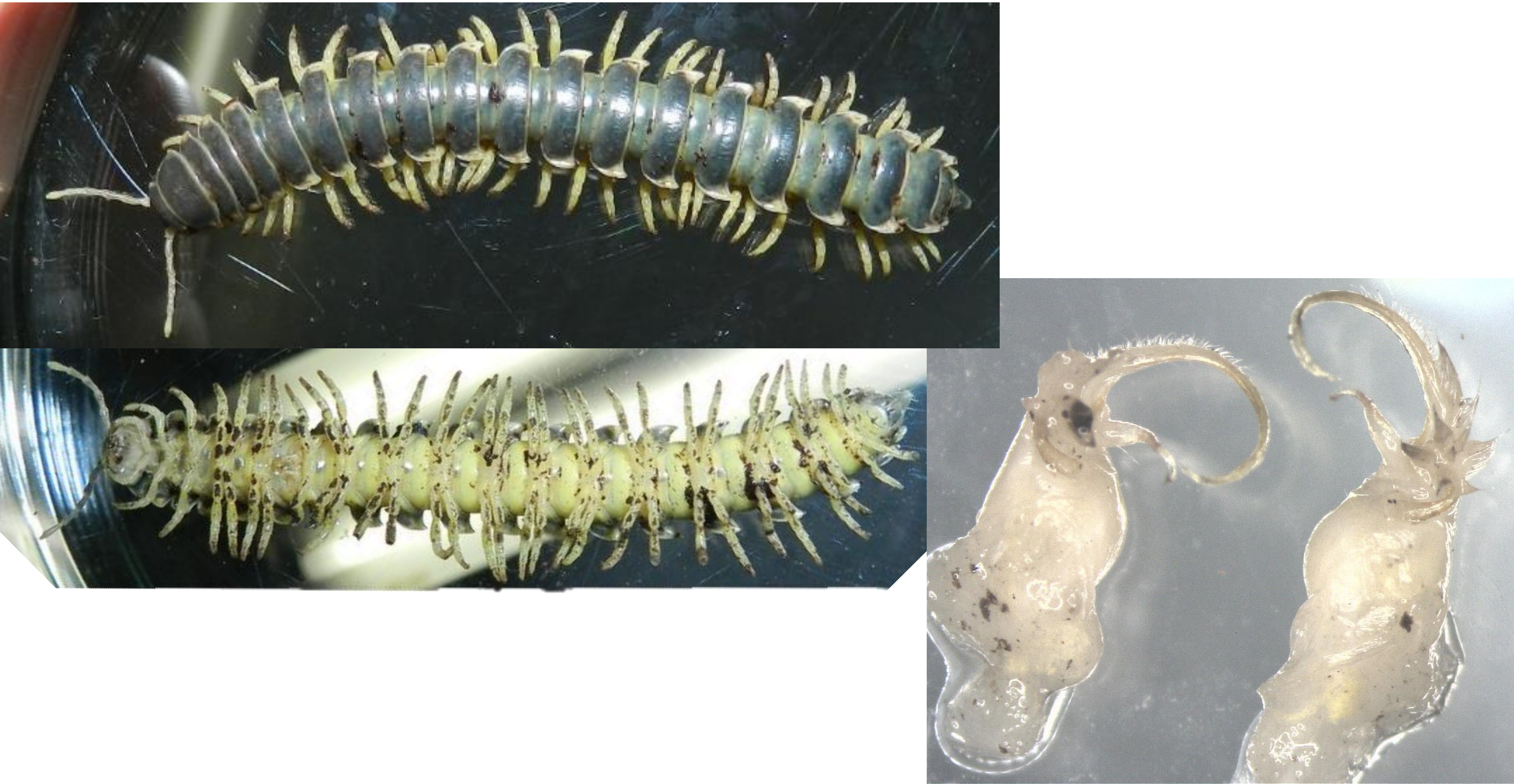

Riukiaria anachoreta Miya
52.3 ± 4.5 mm (N=18)

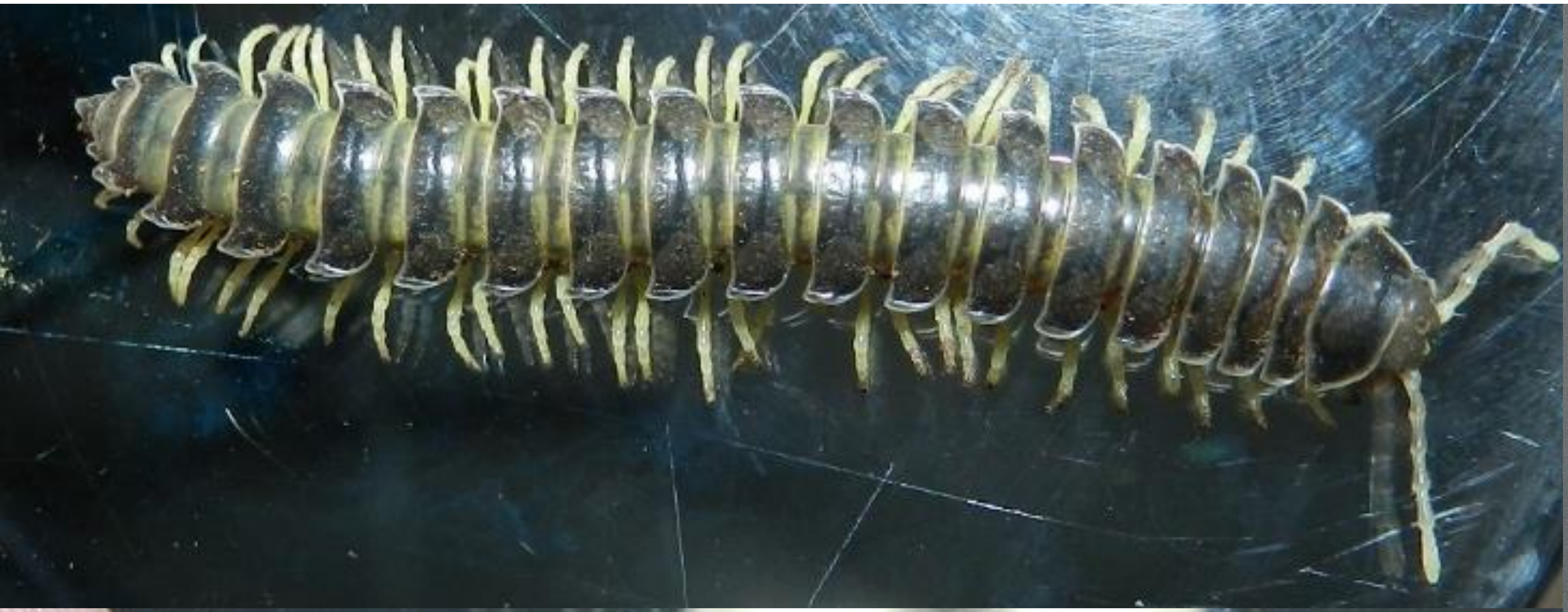
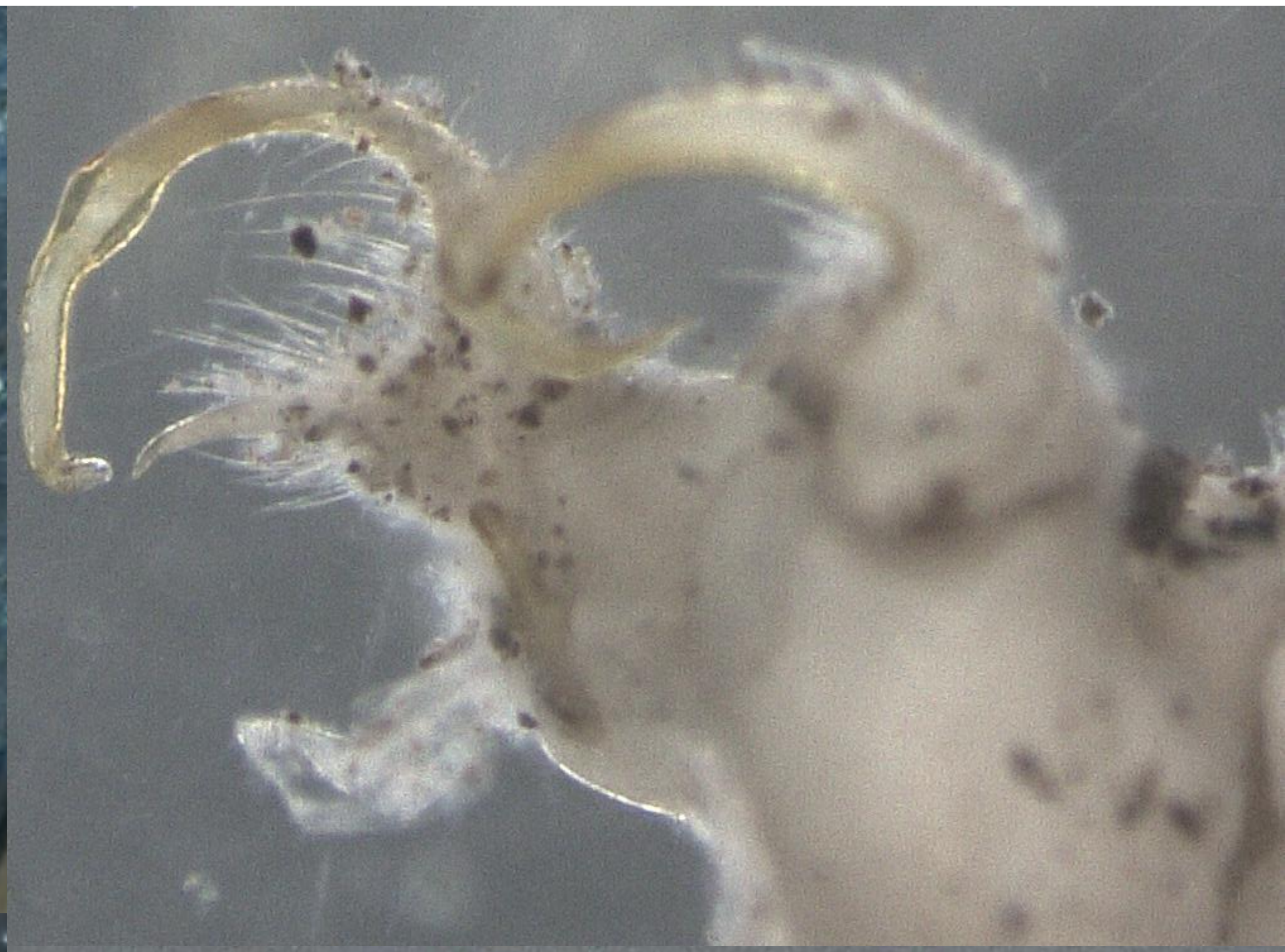
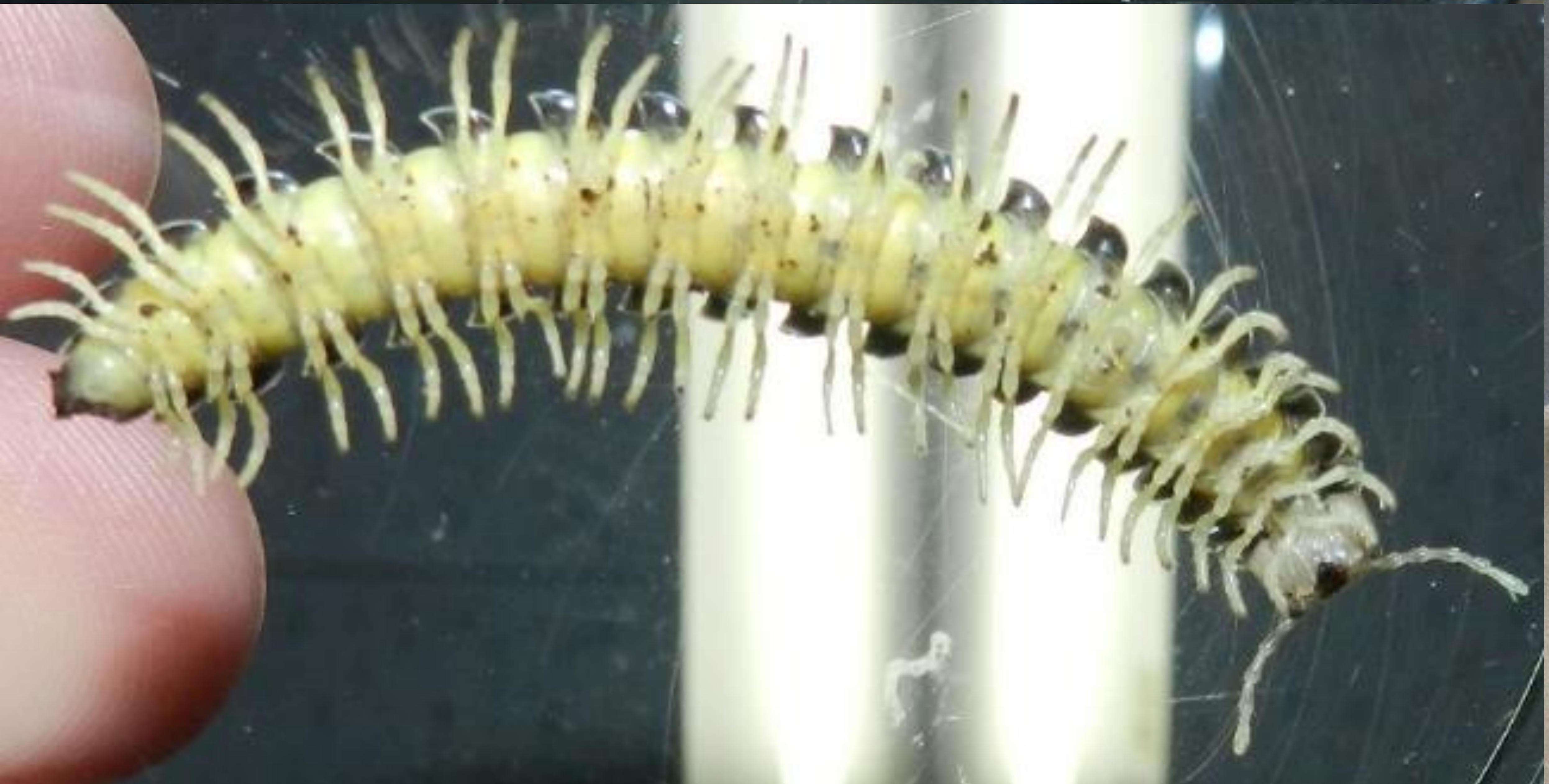
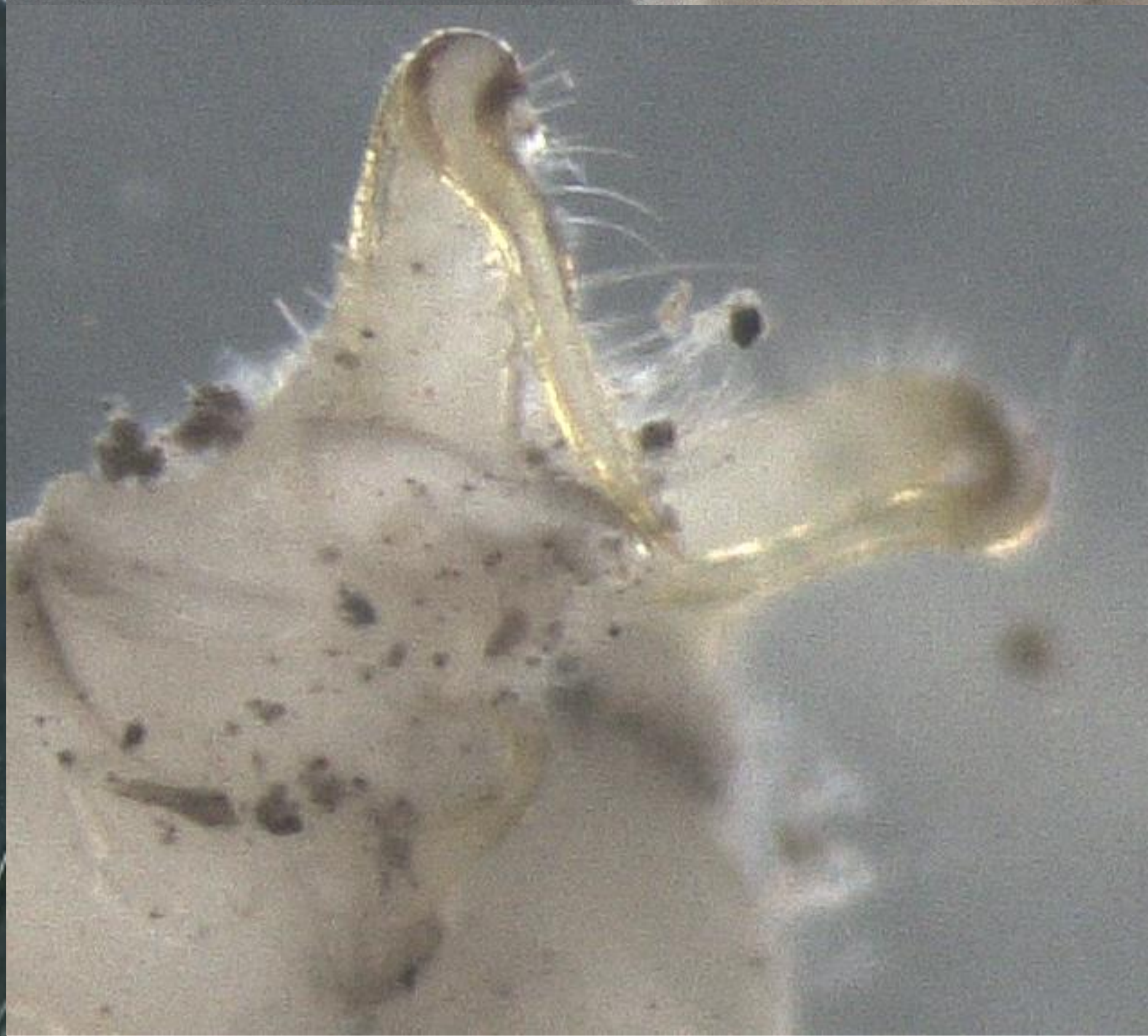

Riukiaria semicircularis semicircularis Miya
43.8 ± 2.5 mm (N=7)

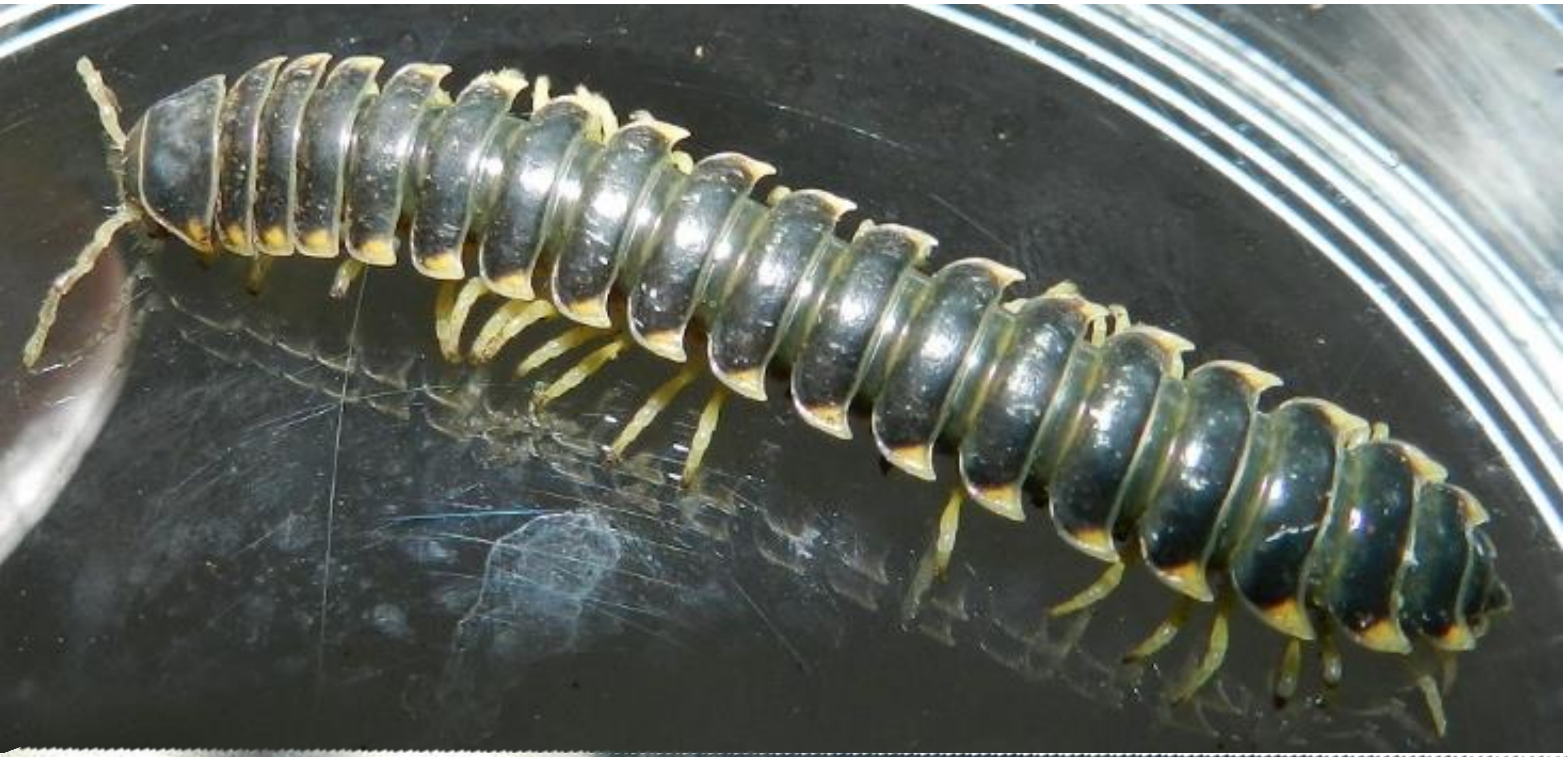

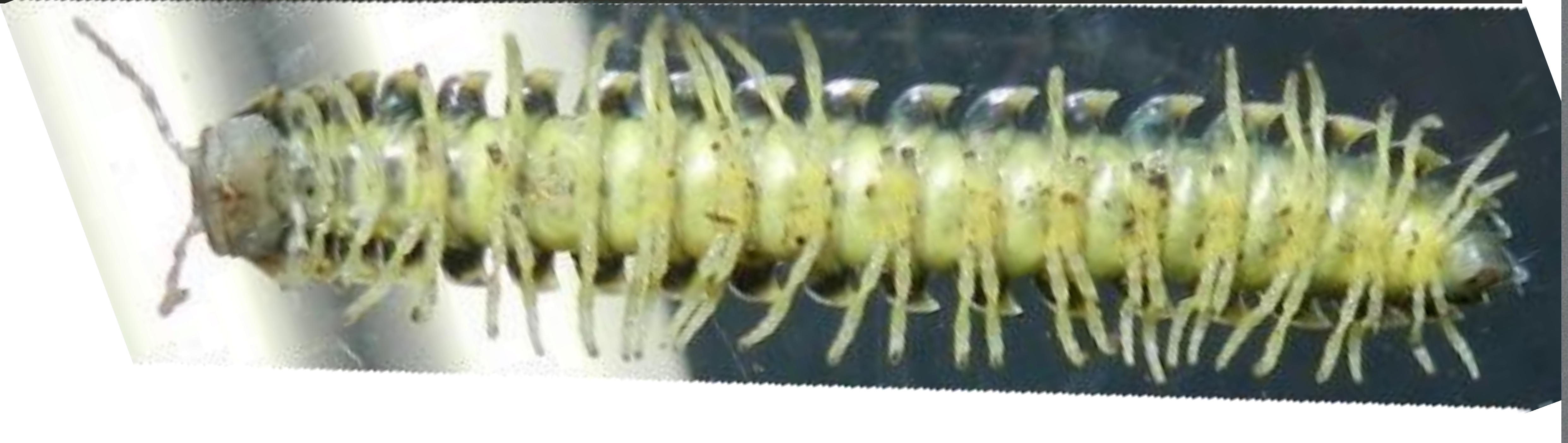

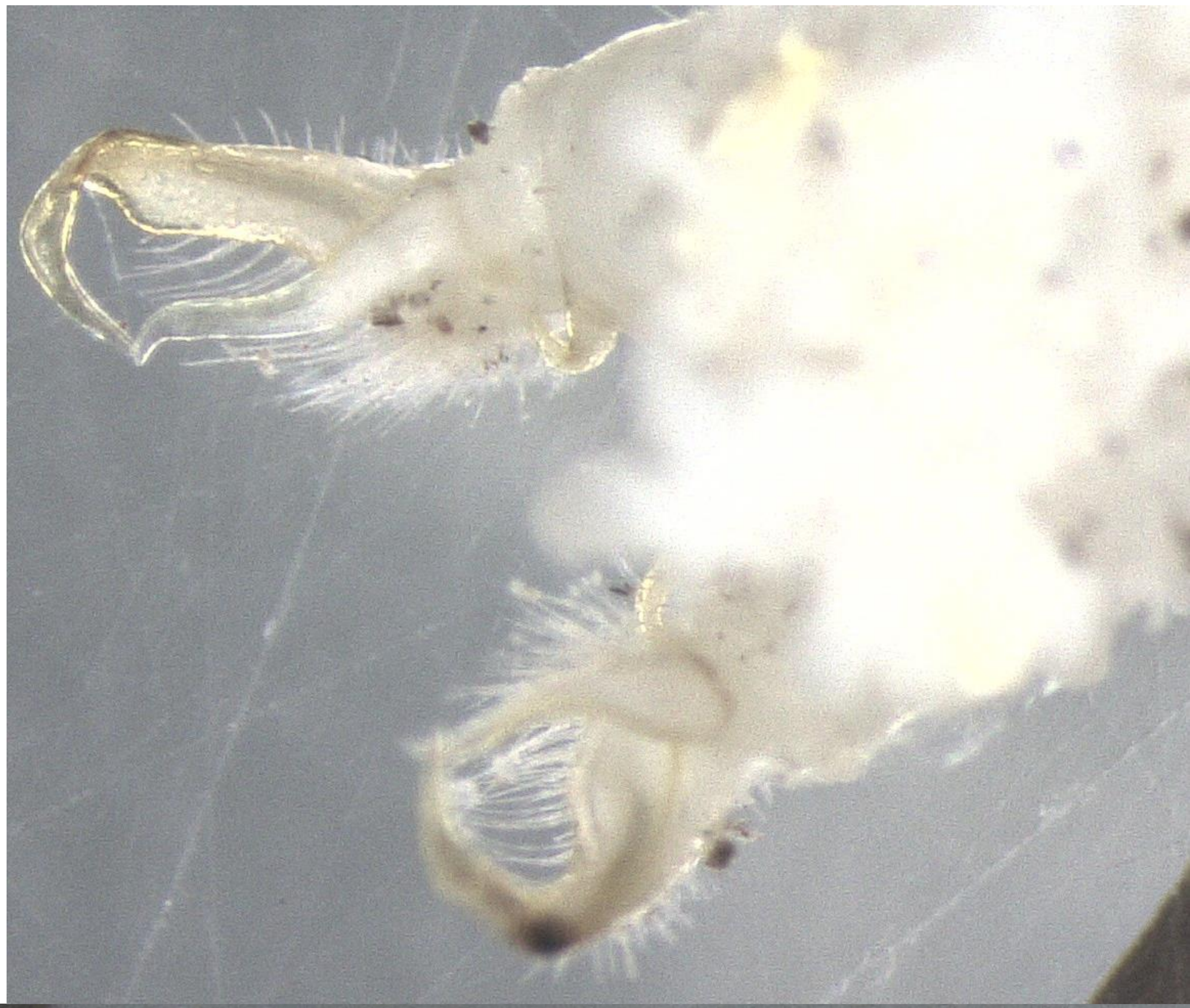

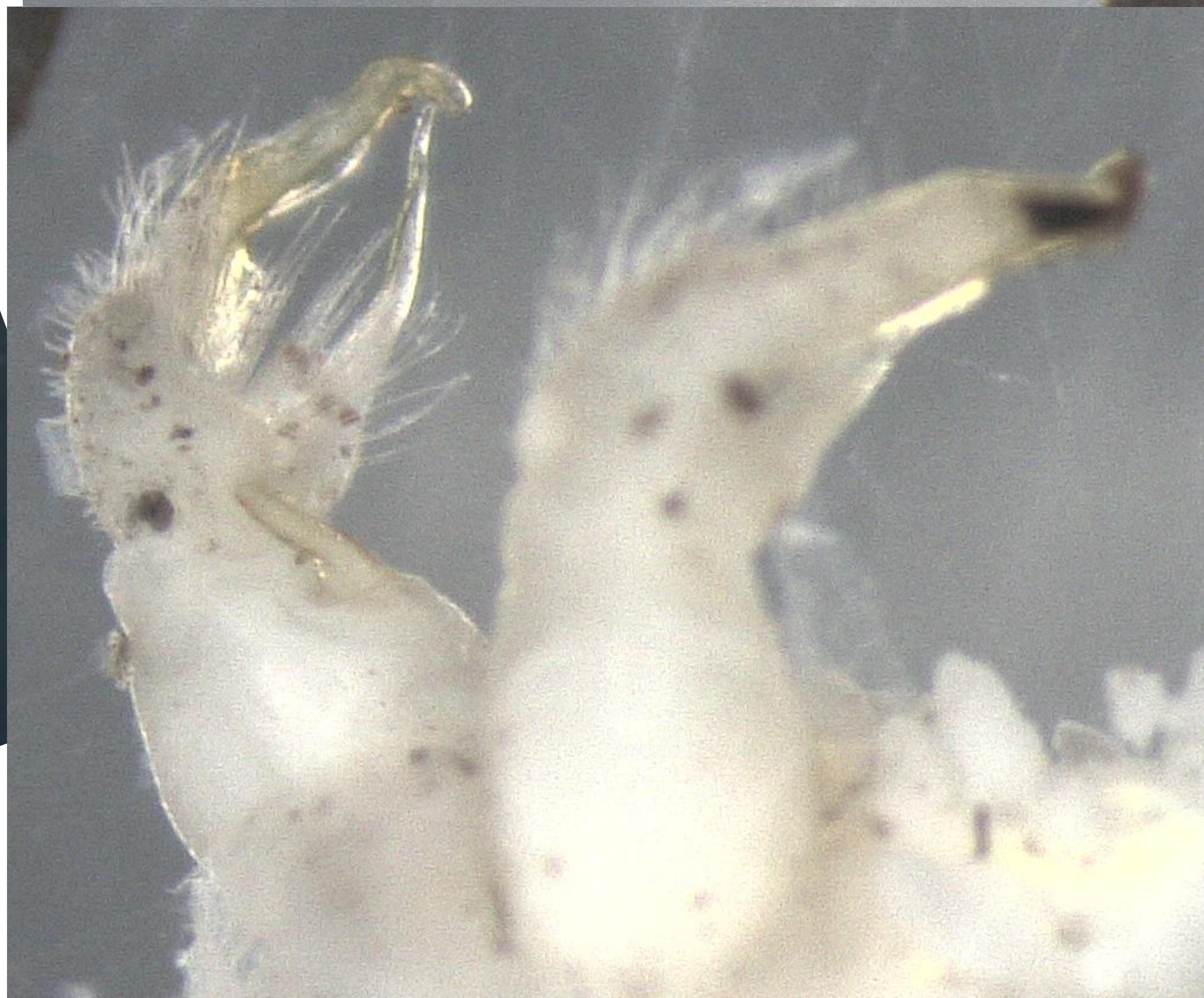

Riukiaria semicircularis semicircularis Shiroyama

39.7±2.4 mm (N=30)

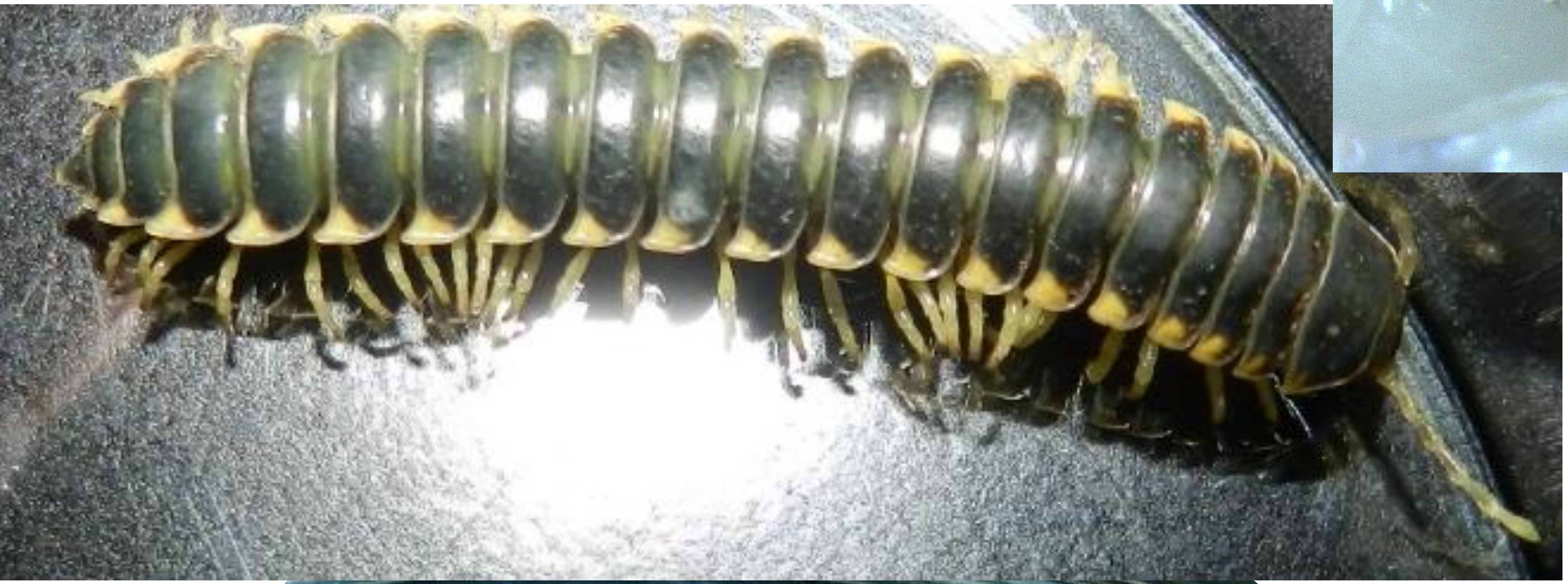

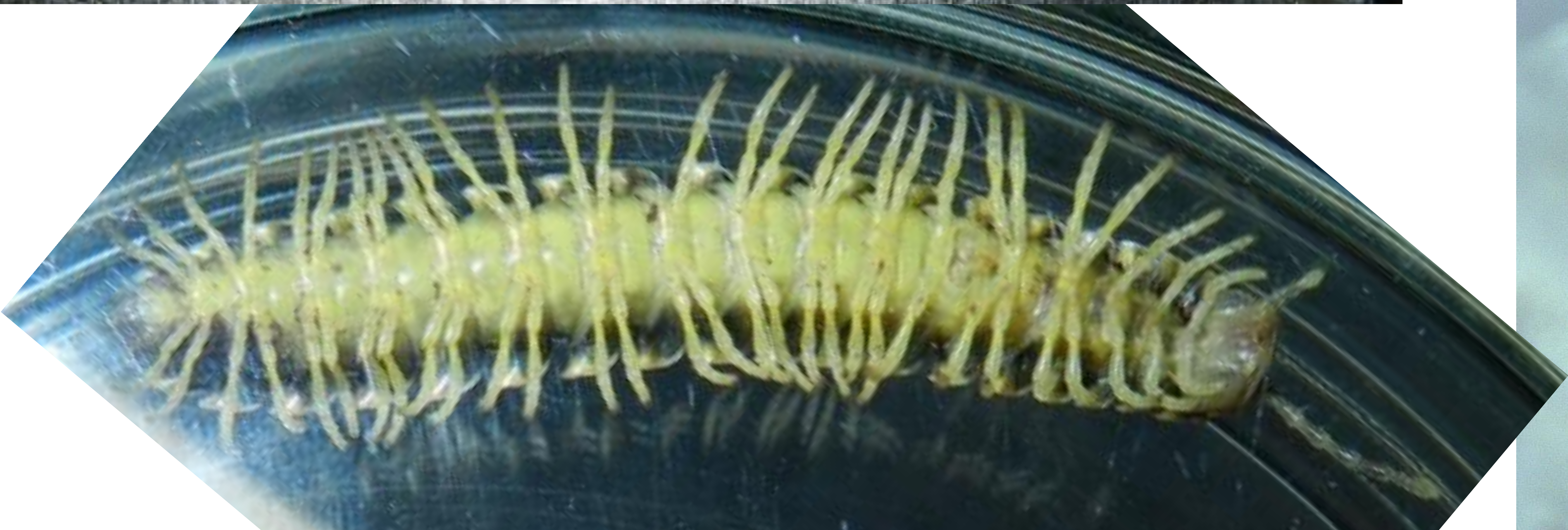

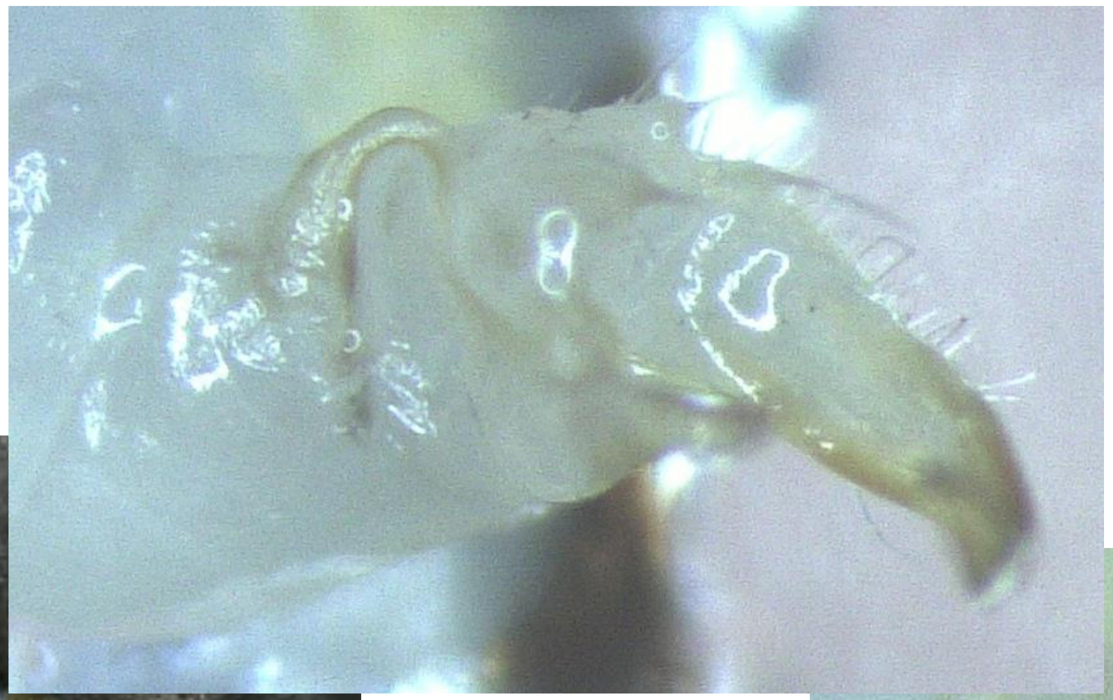

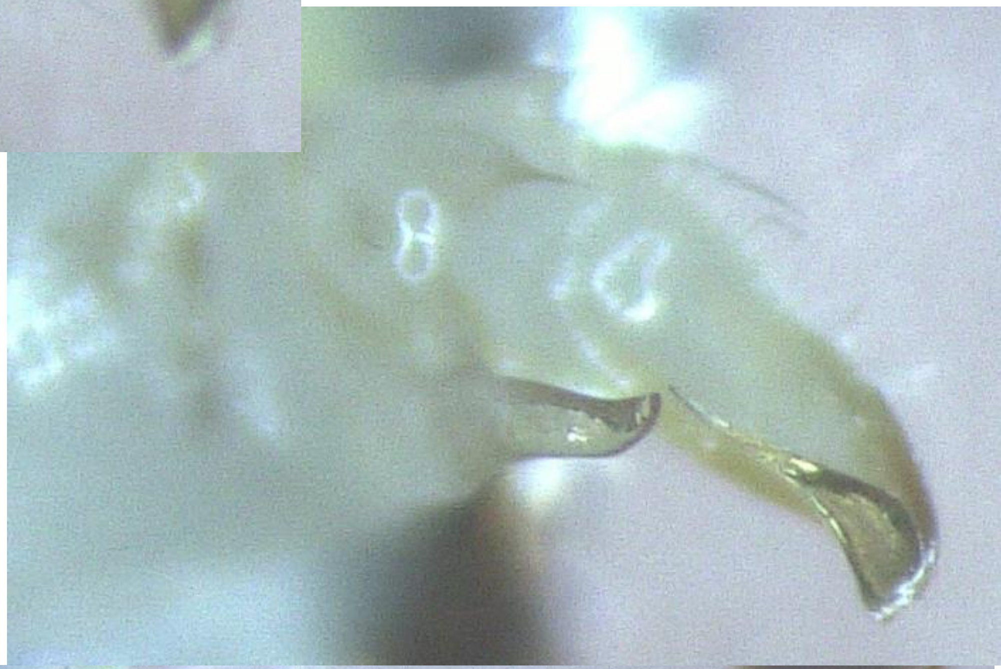

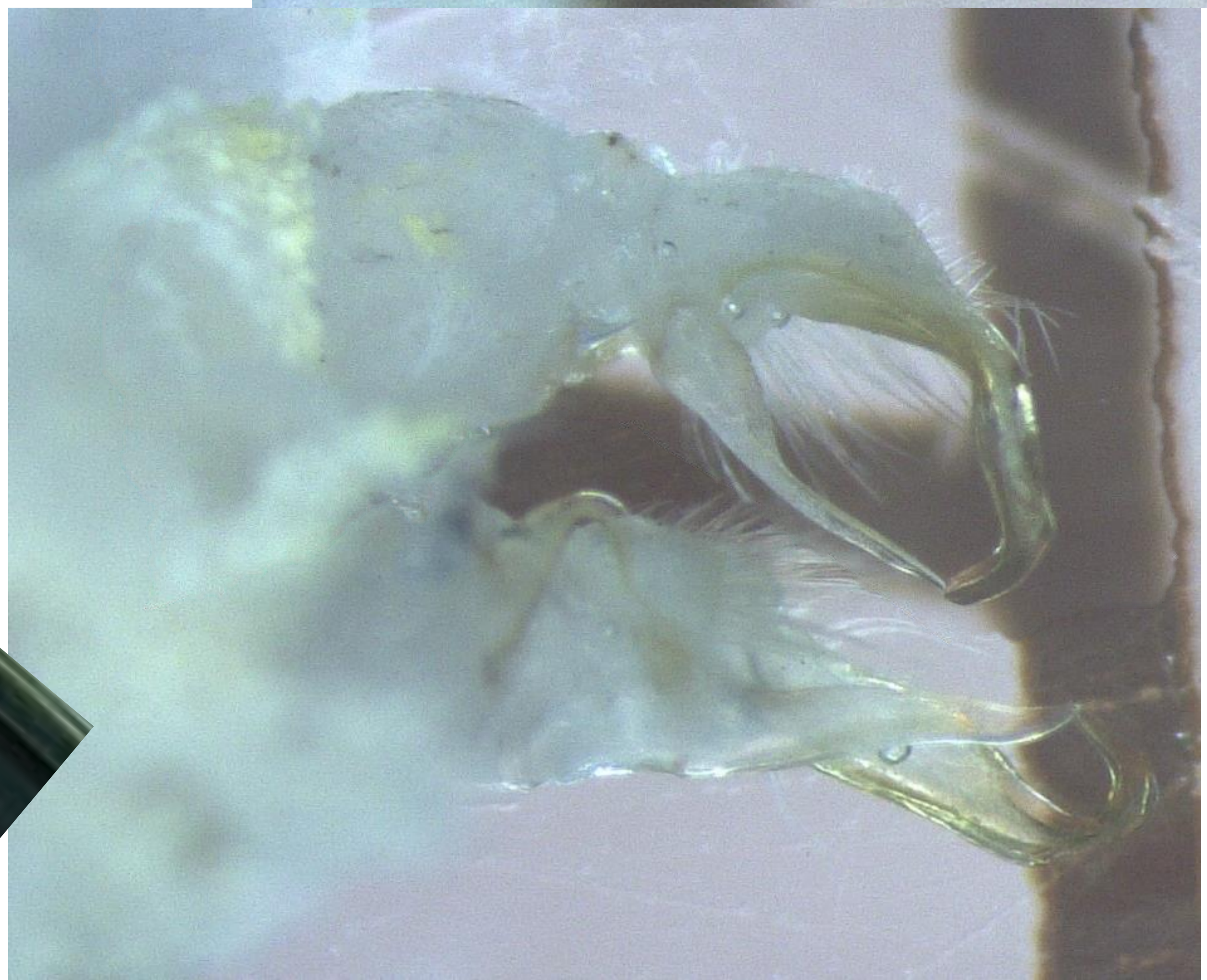

Supplement: Supplementary file 1 — Additional file 1: Figure S1. Photographs of xystodesmid millipedes studied in this experiment. [file 12862_2021_1851_MOESM1_ESM.pdf]
