# Supplementary material for: Symbiosis of the millipede parasitic nematodes Rhigonematoidea and Thelastomatoidea with evolutionary different origins
Source: BMC Ecol Evol. 2021 Jun 12;21:120. doi: 10.1186/s12862-021-01851-4 (PMC8199837; doi:10.1186/s12862-021-01851-4)
Supplement: Supplementary file 2 — Additional file 2: Figure S2. Average ± S.D. of host body size (mm) in infection condition (infected with only R. naylae or co-infected with R. naylae and T. claudiae) or in different seasonal conditions (spring or summer). [file 12862_2021_1851_MOESM2_ESM.docx]

**
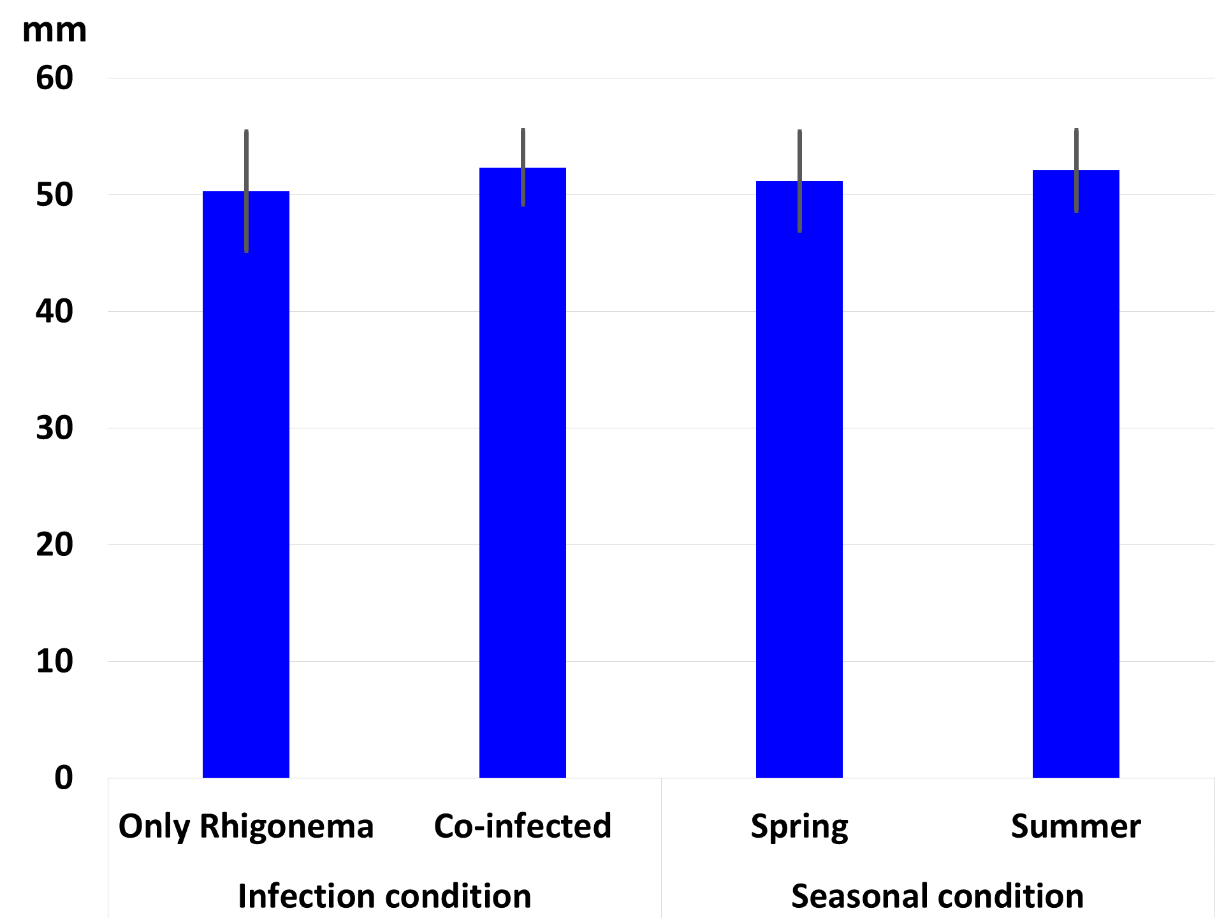
**

**Figure S2.** Average ± S.D. of host body size (mm) in infection condition (infected with only *R. naylae* or co-infected with *R. naylae* and *T. claudiae*) or in different seasonal condition (spring or summer).
