# Supplementary material for: Symbiosis of the millipede parasitic nematodes Rhigonematoidea and Thelastomatoidea with evolutionary different origins
Source: BMC Ecol Evol. 2021 Jun 12;21:120. doi: 10.1186/s12862-021-01851-4 (PMC8199837; doi:10.1186/s12862-021-01851-4)
Supplement: Supplementary file 3 — Additional file 3: Table S1. Pairwise differences % in the D2D3 sequence (730 bp) between 15 samples of Rhigonematoidea spp. [file 12862_2021_1851_MOESM3_ESM.docx]

**Table S1. Pairwise difference % in the D2D3 sequence (730 bp) between 15 samples of Rhigonematoidea spp.**

|  | 1 | 2 | 3 | 4 | 5 | 6 | 7 | 8 | 9 | 10 | 11 | 12 | 13 | 14 | 15 |
| --- | --- | --- | --- | --- | --- | --- | --- | --- | --- | --- | --- | --- | --- | --- | --- |
| 1. KX844643.1 *Rhigonema naylae* |  |  |  |  |  |  |  |  |  |  |  |  |  |  |  |
| **2. MT988354 *Rhigonema naylae*** | 0 |  |  |  |  |  |  |  |  |  |  |  |  |  |  |
| **3. MT988356 *Rhigonema naylae*** | 0 | 0 |  |  |  |  |  |  |  |  |  |  |  |  |  |
| **4. MT988361 *Rhigonema naylae*** | 0 | 0 | 0 |  |  |  |  |  |  |  |  |  |  |  |  |
| **5. MT988364 *Rhigonema naylae*** | 0 | 0 | 0 | 0 |  |  |  |  |  |  |  |  |  |  |  |
| **6. MT988366 *Rhigonema naylae*** | 0 | 0 | 0 | 0 | 0 |  |  |  |  |  |  |  |  |  |  |
| **7. MT988368 *Rhigonema naylae*** | 0 | 0 | 0 | 0 | 0 | 0 |  |  |  |  |  |  |  |  |  |
| **8. MT988371 *Rhigonema naylae*** | 0 | 0 | 0 | 0 | 0 | 0 | 0 |  |  |  |  |  |  |  |  |
| 9. JX131616.1 *Rhigonema ingens* | 2.7 | 2.7 | 2.7 | 2.7 | 2.7 | 2.7 | 2.7 | 2.7 |  |  |  |  |  |  |  |
| 10. JX155274.1 *Xystrognathus phrissus* | 10 | 10 | 10 | 10 | 10 | 10 | 10 | 10 | 11 |  |  |  |  |  |  |
| **11. MT988372 Rhigonematoidea sp. 1** | 11.1 | 11 | 11.1 | 11.1 | 11.1 | 11 | 11.1 | 11 | 12 | 15 |  |  |  |  |  |
| **12. MT988374 Rhigonematoidea sp. 1** | 11.1 | 11 | 11.1 | 11.1 | 11.1 | 11 | 11.1 | 11 | 12 | 15 | 0.5 |  |  |  |  |
| **13. MT988375 Rhigonematoidea sp. 1** | 11 | 11 | 11 | 11 | 11 | 11 | 11 | 11 | 12 | 15 | 0.7 | 0.1 |  |  |  |
| **14. MT988377 Rhigonematoidea sp. 1** | 11.1 | 11 | 11.1 | 11.1 | 11.1 | 11 | 11.1 | 11 | 12 | 15 | 0.5 | 0 | 0.1 |  |  |
| 15. JX155273.1 *Ichthyocephaloides sumbatus* | 11.2 | 11 | 11.2 | 11.2 | 11.2 | 11 | 11.2 | 11 | 12 | 16 | 13 | 12 | 12 | 12 |  |
