# Supplementary material for: Symbiosis of the millipede parasitic nematodes Rhigonematoidea and Thelastomatoidea with evolutionary different origins
Source: BMC Ecol Evol. 2021 Jun 12;21:120. doi: 10.1186/s12862-021-01851-4 (PMC8199837; doi:10.1186/s12862-021-01851-4)
Supplement: Supplementary file 4 — Additional file 4: Table S2. Pairwise differences % in the D2D3 sequence (704 bp) between 13 samples of Travassosinematidae spp. [file 12862_2021_1851_MOESM4_ESM.docx]

**Table S2. Pairwise difference % in the D2D3 sequence (704 bp) between 13 samples of Travassosinematidae spp.**

|  | 1 | 2 | 3 | 4 | 5 | 6 | 7 | 8 | 9 | 10 | 11 | 12 | 13 |
| --- | --- | --- | --- | --- | --- | --- | --- | --- | --- | --- | --- | --- | --- |
| 1. KX844645.1*Travassosinema claudiae* |  |  |  |  |  |  |  |  |  |  |  |  |  |
| **2. MT988321 *Travassosinema claudiae*** | 0 |  |  |  |  |  |  |  |  |  |  |  |  |
| **3. MT988324 *Travassosinema claudiae*** | 0 | 0 |  |  |  |  |  |  |  |  |  |  |  |
| **4. MT988327 *Travassosinema claudiae*** | 0 | 0 | 0 |  |  |  |  |  |  |  |  |  |  |
| **5. MT988330 *Travassosinema claudiae*** | 0 | 0 | 0 | 0 |  |  |  |  |  |  |  |  |  |
| **6. MT988332 *Travassosinema claudiae*** | 0 | 0 | 0 | 0 | 0 |  |  |  |  |  |  |  |  |
| **7. MT988334 *Travassosinema claudiae*** | 0 | 0 | 0 | 0 | 0 | 0 |  |  |  |  |  |  |  |
| **8. MT988337 *Travassosinema claudiae*** | 0 | 0 | 0 | 0 | 0 | 0 | 0 |  |  |  |  |  |  |
| **9. MT988339 *Travassosinema claudiae*** | 0.1 | 0.1 | 0.1 | 0.1 | 0.1 | 0.1 | 0.1 | 0.1 |  |  |  |  |  |
| **10. MT988342 *Travassosinema claudiae*** | 0.1 | 0.1 | 0.1 | 0.1 | 0.1 | 0.1 | 0.1 | 0.1 | 0 |  |  |  |  |
| **11. MT988345 *Travassosinema claudiae*** | 0.1 | 0.1 | 0.1 | 0.1 | 0.1 | 0.1 | 0.1 | 0.1 | 0 | 0 |  |  |  |
| **12. MT988348 *Travassosinema claudiae*** | 0.6 | 0.6 | 0.6 | 0.6 | 0.6 | 0.6 | 0.6 | 0.6 | 0.4 | 0.4 | 0.4 |  |  |
| 13. HM769761.1*Travassosinema dalei* | 3.7 | 3.7 | 3.7 | 3.7 | 3.7 | 3.7 | 3.7 | 3.6 | 3.6 | 3.6 | 3.6 | 3.8 |  |
