# Supplementary material for: Symbiosis of the millipede parasitic nematodes Rhigonematoidea and Thelastomatoidea with evolutionary different origins
Source: BMC Ecol Evol. 2021 Jun 12;21:120. doi: 10.1186/s12862-021-01851-4 (PMC8199837; doi:10.1186/s12862-021-01851-4)
Supplement: Supplementary file 5 — Additional file 5: Table S3. Population of the two parasitic nematodes in Parafontaria millipedes. [file 12862_2021_1851_MOESM5_ESM.docx]

**Table S3** **Population of the two parasitic nematodes in *Parafontaria* millipedes**

| Host | Parasitic nematodes | | | | | |
| --- | --- | --- | --- | --- | --- | --- |
|  |  | Male^1^ | Female^1^ | Juvenile^1^ | Total Prevanence^2^ | Co-infection Prevalence |
| *P. laminata*  Kinka  N = 35 | *R. naylae* | 2.92 (2.15-4.15) | 2.69 (1.85-3.69) | 12.33 (7.33-23.08) | 74 %  N = 26 | 14 %  N = 5 |
|  |  | 37 % (N = 13) | 37 % (N = 13) | 69 % (N = 24) |  |  |
|  | *T. claudiae* | - | 1.5 (1.00-2.00) | 1 | 14 %  N = 5 |  |
|  |  | 0 % (N = 0) | 11 % (N = 4) | 35 % (N = 1) |  |  |
| *P. tonominea*  species complex Kinka N = 43 | *R. naylae* | 9.92 (8.00-12.72) | 11.05 (9.03-13.46) | 6.69 (4.97 - 9.00) | 95 %  N = 41 | 47 %  N = 20 |
|  |  | 91 % (N = 39) | 91 % (N = 39) | 81 % (N = 35) |  |  |
|  | *T. claudiae* | - | 1.89 (1.44-2.33) | 5.00 (1.00-2.20) | 49 %  N = 21 |  |
|  |  | 0 % (N = 0) | 42 % (N = 18) | 12 % (N = 5) |  |  |
| *P. tonominea*  species complex Hyaku N = 22 | *R. naylae* | 11.45 (9.64-15.27) | 11,82 (9.5-14.00) | 13.77 (10.41-17.91) | 100 %  N = 22 | 46 %  N = 10 |
|  |  | 100 % (N = 22) | 100 % (N = 22) | 100 % (N = 22) |  |  |
|  | *T. claudiae* | 1 | 2.50 (1.38-4.63) | 1 | 46 %  N = 10 |  |
|  |  | 36 % (N = 8) | 4.5 % (N = 1) | 4.5 % (N = 1) |  |  |
| *P. tonominea*  species complex  Embara N = 20 | *R. naylae* | 7.17 (4.89-10.61) | 6.05 (4.47-7.58) | 2.93 (1.87-5.20) | 100 %  N = 20 | 25 %  N = 5 |
|  |  | 90 % (N = 18) | 95 % (N = 19) | 75 % (N = 15) |  |  |
|  | *T. claudiae* | - | 1.64 (1.18-2.27) | 1 | 55 %  N = 11 |  |
|  |  | 0 % (N = 0) | 55 % (N = 11) | 5 % (N = 1) |  |  |
| *P. longa*  Embara  N = 7 | *R. naylae* | 5.75 (4.00-6.50) | 7.60 (3.40-11.80) | 7.20 (3.20-13.60) | 71 %  N = 5 | 43 %  N = 3 |
|  |  | 57 % (N = 4) | 71 % (N = 5) | 71 % (N = 5) |  |  |
|  | *T. claudiae* | - | 7.67 (4.00-9.33)* | 2 | 43 %  N=3 |  |
|  |  | 0 % (N=0) | 43 % (N=3) | 14 % (N=1) |  |  |

^1^ Mean intensities and confidence intervals with 95% confidence limit (in brackets) were calculated by Bootstrap Confidence interval method.

^2^ % of the infected millipede among all millipede examined.

* Confidence intervals were not calculated if the sample size was too small.
