# Supplementary material for: Symbiosis of the millipede parasitic nematodes Rhigonematoidea and Thelastomatoidea with evolutionary different origins
Source: BMC Ecol Evol. 2021 Jun 12;21:120. doi: 10.1186/s12862-021-01851-4 (PMC8199837; doi:10.1186/s12862-021-01851-4)
Supplement: Supplementary file 6 — Additional file 6: Table S4. Pairwise differences % in the D2D3 sequence (676 bp) between eight samples of Thelastomatidae spp. [file 12862_2021_1851_MOESM6_ESM.docx]

**Table S4. Pairwise difference % in the D2D3 sequence (676 bp) between eight samples of Thelastomatidae spp.**

|  | 1 | 2 | 3 | 4 | 5 | 6 | 7 | 8 |
| --- | --- | --- | --- | --- | --- | --- | --- | --- |
| 1. KP172224.1 *Thelastoma bulhoesi* |  |  |  |  |  |  |  |  |
| 2. **MT988315 Thelastomatidae sp. 2** | 7.8 |  |  |  |  |  |  |  |
| 3. **MT988316 Thelastomatidae sp. 2** | 7.8 | 0.0 |  |  |  |  |  |  |
| 4. **MT988319 Thelastomatidae sp. 2** | 8.0 | 0.1 | 0.1 |  |  |  |  |  |
| 5. **MT988313 Thelastomatidae sp. 1** | 10.5 | 9.5 | 9.5 | 9.6 |  |  |  |  |
| 6. HM016661.1 *Stauratostoma shelleyi* | 11.4 | 11.2 | 11.2 | 11.2 | 12.3 |  |  |  |
| 7. HN190721.1 *Aorurus agile* | 14.1 | 13.6 | 13.6 | 13.6 | 12.6 | 13.9 |  |  |
| 8. HN191235.1 *Aoruroides chubudaigaku* | 17.2 | 17.5 | 17.5 | 17.6 | 16.1 | 17.6 | 17.6 |  |
