# Supplementary material for: Symbiosis of the millipede parasitic nematodes Rhigonematoidea and Thelastomatoidea with evolutionary different origins
Source: BMC Ecol Evol. 2021 Jun 12;21:120. doi: 10.1186/s12862-021-01851-4 (PMC8199837; doi:10.1186/s12862-021-01851-4)
Supplement: Supplementary file 7 — Additional file 7: Table S5. Population of the parasitic nematodes in Riukiaria millipedes. [file 12862_2021_1851_MOESM7_ESM.docx]

**Table S5 Population of the parasitic nematodes in *Riukiaria* millipedes**

| Host | Parasitic nematodes | | | | | |
| --- | --- | --- | --- | --- | --- | --- |
|  |  | Male intensities^1^  and prevalences^2^ | Female intensities^1^  and prevalences^2^ | Juvenile intensities^1^  and prevalences^2^ | Total Prevanence^2^ | Co-infection Prevalences^3^ |
| *R. cornuta*  Yamaga  N = 31 | Rhigonematoidea sp. 1 | 18.35 (15.16-22.03) | 26.61 (21.81-32.26) | 12.10 (9.13-16.06) | 100 %  N = 31 | R&Tc: 84 %  R&The: 87 %  Tc&The: 77 %  Triple: 77 % |
|  |  | 100 % (N = 31) | 100 % (N = 31) | 100 % (N = 31) |  |  |
|  | *T. claudiae* | 1.00 | 5.23 (3.96-6.73) | 12.00 | 84 %  N = 26 |  |
|  |  | 13 % (N = 4) | 84 % (N = 26) | 3 % (N = 1) |  |  |
|  | Thelastomatidae sp. 1 | 1.00 | 3.37 (2.67-4.41) | - | 87 %  N = 27 |  |
|  |  | 3 % (N = 1) | 87 % (N = 27) | 0 % (N = 0) |  |  |
| *R. anachoreta*  Miyanoura  N = 20 | Rhigonematoidea sp. 1 | 8.63 (6.53-11.11) | 10.26 (8.21-13.42) | 2.75 (1.88-3.69) | 95 %  N = 19 | R&Tc: 55 %  R&The: 65 %  Tc&The: 35 %  Triple: 35 % |
|  |  | 95 % (N = 19) | 95 % (N = 19) | 80 % (N = 16) |  |  |
|  | *T. claudiae* | - | 4.00 (2.25-6.33) | - | 60 %  N = 12 |  |
|  |  | 0 % (N = 0) | 60 % (N = 12) | 0 % (N = 0) |  |  |
|  | Thelastomatidae sp. 2 | - | 2.69 (1.69-5.00) | - | 65 %  N = 13 |  |
|  |  | 0 % (N = 0) | 65 % (N = 13) | 0 % (N = 0) |  |  |
| *R. semicircularis*  Miyanoura  N = 9 | Rhigonematoidea sp. 1 | 5.67 (3.78-9.00) | 7.22 (4.56-10.22) | 5.33 (1.50-12.17) | 100 %  N = 9 | R&Tc: 44 %  R&The: 44 %  Tc&The: 11 %  Triple: 11 % |
|  |  | 100 % (N = 9) | 100 % (N = 9) | 67 % (N = 6) |  |  |
|  | *T. claudiae* | - | 2.00 | - | 44 %  N = 4 |  |
|  |  | 0 % (N = 0) | 44 % (N = 4) | 0 % (N = 0) |  |  |
|  | Thelastomatidae sp. 2 | - | 1.50 (1.00-2.00) | - | 44 %  N = 9 |  |
|  |  | 0 % (N = 0) | 44 % (N = 4) | 0 % (N = 0) |  |  |
| *R. semicircularis*  Shiroyama  N = 38 | Rhigonematoidea sp. 1 | 5.46 (4.46-6.54) | 6.92 (5.86-8.25) | 3.74 (3.06 - 4.35) | 97 %  N = 37 | R&Tc: 58 %  R&The: 45 %  Tc&The: 21%  Triple: 21% |
|  |  | 95 % (N = 36) | 97 % (N = 37) | 82 % (N = 31) |  |  |
|  | *T. claudiae* | - | 2.09 (1.68-2.45) | 1.00 | 61 %  N = 23 |  |
|  |  | 0 % (N = 0) | 58 % (N = 22) | 5 % (N = 2) |  |  |
|  | Thelastomatidae sp. 2 | 1.00 | 1.44 (1.13-1.81) | 1.50 | 45 %  N = 17 |  |
|  |  | 8 % (N = 3) | 42 % (N = 16) | 5 % (N = 2) |  |  |

^1^ Mean intensities and confidence intervals with 95% confidence limit (in brackets) were calculated by Bootstrap Confidence interval method.

^2^ % of the infected millipede among all millipede examined.

3 R&Tc, co-infected with Rhigonematoidea and *T. claudiae*; R&The, co-infected with Rhigonematoidea and Thelastomatidae; Tc&The, co-infected with *T. claudiae* and Thelastomatidae; Triple, co-infected with Rhigonematoidea, *T. claudiae*, and Thelastomatidae.

* Confidence intervals were not calculated if the sample size was too small.
