# Supplementary material for: Symbiosis of the millipede parasitic nematodes Rhigonematoidea and Thelastomatoidea with evolutionary different origins
Source: BMC Ecol Evol. 2021 Jun 12;21:120. doi: 10.1186/s12862-021-01851-4 (PMC8199837; doi:10.1186/s12862-021-01851-4)
Supplement: Supplementary file 8 — Additional file 8: Table S6. List of DNA sequence used for molecular phylogenetic analysis. [file 12862_2021_1851_MOESM8_ESM.docx]

**Table S6. List of DNA sequence used for molecular phylogenetic analysis**

**OXYURIDOMORPHA**

Oxyuroidea

MF102080 *Parapharyngodon bainae*

JF829241 *Parapharyngodon echinatus*

MH459227 *Parapharyngodon micipsae*

KJ778089 *Thelandros tinerfensis*

MH459217 *Thelandros filiformis*

MT341274 *Batracholandros salamandrae*

**Thelastomatoidea**

Thelastomatidae

KX752430 *Hammerschmidtiella keeneyi*

EU365628 *Hammerschmidtiella diesingi*

GQ368460 *Malaspinanema* sp.

GQ368461 *Blattophila* sp.

KX752428 *Blattophila peregrinate*

MN744378 *Leidynemella shahi*

KC540759 *Leidynema appendiculata*

GQ401114 *Leidynema portentosae*

MN191235 *Aoruroides chubudaigaku*

MN190721 *Aorurus agile*

MH016661 *Stauratostoma shelleyi*

KP172224 *Thelastoma bulhoesi*

MT988315 *Thelastomatidae* sp. 2

MT988316 *Thelastomatidae* sp. 2

MT988319 *Thelastomatidae* sp. 2

MT988313 *Thelastomatidae* sp. 1

LC214837 *Thelastoma* sp.

MG189597 *Thelastoma krausi*

*Blatticola*

GQ368472 *Blatticola blattae*

Travassosinematidae

MT988321 *Travassosinema claudiae*

MT988324 *Travassosinema claudiae*

MT988327 *Travassosinema claudiae*

MT988330 *Travassosinema claudiae*

MT988332 *Travassosinema claudiae*

MT988334 *Travassosinema claudiae*

MT988337 *Travassosinema claudiae*

MT988339 *Travassosinema claudiae*

MT988342 *Travassosinema claudiae*

MT988345 *Travassosinema claudiae*

MT988348 *Travassosinema claudiae*

GQ368471 *Travassosinema* sp.

HM769761 *Travassosinema dalei*

*Cephalobellus*

GQ368470 *Cameronia multiovata*

MF668725 *Cephalobellus brevicaudatus*

MT988351 *Cephalobellus* sp.1

Hystrignathidae

MH244508 *Coynema poeyi*

MH411129 *Hystrignathus rigidus*

MH569782 *Lepidonema magnum*

MH569781 *Xyo pseudohystrix*

KX427531 *Longior similis*

Pseudonymidae

MN401735 *Zonothrix columbianus*

KF771649 *Pseudonymus spirotheca*

KF771648 *Pseudonymus islamabadi*

Protrelloididae

MN401734 *Protrellus browni*

KU674961 *Protrellus* sp.

**ASCARIDOMORPHA**

Seuratoidea

KX752091 *Cucullanus opisthoporus*

KX752093 *Cucullanus grandistomis*

KY857891 *Truttaedacnitis truttae*

MN526257 *Paraquimperia* sp.

KY476350 *Ichtyobronema hamulatum*

**RHIGONEMATOMORPHA**

Ransomnematoidea

KY857884 *Heth tuxtlensis*

KY857886 *Heth xarochae*

KY857880 *Heth gordae*

JX946704 *Heth taybaci*

KM226162 *Heth impalutiensis*

KY857884 *Heth tuxtlensis*

KY985472 *Heth initiensis*

KY985470 *Heth konoplevi*

KT957944 *Carnoya philippinensis*

KT957945 *Carnoya philippinensis*

JX946703 *Carnoya filipjevi*

KT236088 *Carnoya mexicana*

KT236086 *Carnoya cepacapitatus*

JX999732 *Brumptaemilius justini*

JX436471 *Insulanema longispiculum*

JX436470 *Cattiena fansipanis*

JX419378 *Cattiena trachelomegali*

Cosmocercoidea

LC018444 *Cosmocercoides pulcher*

AB908160 *Cosmocercoides tonkinensis*

MH878688 *Probstmayria* sp.

Rhigonematoidea

MT988354 *Rhigonema naylae*

MT988356 *Rhigonema naylae*

MT988361 *Rhigonema naylae*

MT988364 *Rhigonema naylae*

MT988366 *Rhigonema naylae*

MT988368 *Rhigonema naylae*

MT988371 *Rhigonema naylae*

MT988372 Rhigonematoidea sp. 1

MT988374 Rhigonematoidea sp. 1

MT988375 Rhigonematoidea sp. 1

MT988377 Rhigonematoidea sp. 1

JX155273 *Ichthyocephaloides sumbatus*

JX155274 *Xystrognathus phrissus*

KU561100 *Obainia* sp.

KX844643 *Rhigonema naylae*

JX131616 *Rhigonema ingens*

**GNATHOSTOMATOMORPHA**

Gnathostomatidae

MN526258 *Tanqua* sp.

MW550279 *Spiroxys ankarafantsika*

Anguillicolidae

FJ748546 *Anguillicoloides papernai*

FJ748545 *Anguillicola globiceps*

FJ748547 *Anguillicoloides crassus*

**SPIRUROMORPHA**

Filarioidea

XR_002251420 *Loa loa*

MN432520 *Mansonella perstans*

KY990015 *Dirofilaria immitis*

Physalopteroidea

MG808041 *Physaloptera* sp.

KY990020 *Turgida torresi*

Spiruroidea

MG818763 *Mastophorus muris*

KM434335 *Cylicospirura petrowi*

LR215834 *Gongylonema pulchrum*

Thelazioidea

AY751500 *Spirocerca lupi*

**Camallanoidea**

Camallanidae

MG947389 *Camallanus xenopodis*

MG947390 *Procamallanus slomei*

MN525305 *Batrachocamallanus xenopodis*

MT901636 *Spirocamallanus inopinatus*

**Dracunculoidea**

Phirometridae

MH791052 *Philometra rischta*

MH791048 *Philometroides moraveci*

Skriabillanidae

MH791057 *Skrjabillanus tincae*

MH791055 *Skrjabillanus scardinii*

MH791056 *Kalmanmolnaria intestinalis*

**(The part shown in red is the added DNA sequence)**

**Correction history (DNA Sequence that are no longer used)**

**Out group**

MN519140 *Caenorhabditis elegans*

**OXYURIDOMORPHA**

Oxyuroidea

KX550056 *Skrjabinodon* sp.

KY990018 *Passalurus ambiguous*

KY990021 *Oxyuris equi*

KJ632667 *Ozolaimus linstowi*

KY990019 *Skrjabinema ovis*

Coronostomatoidea

MH244505 *Coronostoma claireae*

Thelastomatoidea

EU365629 *Hammerschmidtiella cristata*

KX024651 *Suifunema* sp.

EU365631 *Severianoia blapticola*

EU365632 *Cranifera cranifera*

FJ936558.1 *Aoruroides* sp.

**ASCARIDOMORPHA**

Seuratoidea

MG182630 *Dichelyne mexicanus*

Ascaridoidea

AB558480 *Raphidascaris gigi*

MK558800 *Hysterothylacium reliquens*

KX859078 *Raphidascaris lanfrediae*

FJ418790 *Toxocara vitulorum*

KY826440 *Anisakis physeteris*

JQ673267 *Pseudoterranova decipiens*

AY210806 *Ascaris lumbricoides*

GU245691 *Krefftascaris sharpiloi*

GU245688 *Krefftascaris parmenteri*

Heterakoidea

LC133188 *Strongyluris calotis*

LC185992 *Meteterakis occidentalis*

KY990014 *Ascaridia galli*

**SPIRUROMORPHA**

Spiruroidea

LC278392 *Gongylonema nepalensis*

Filarioidea

MN432519 *Mansonella ozzardi*

**Dracunculoidea**

Dracunculidae

KY990016 *Dracunculus* sp.
